# Two-Photon–Near Infrared-II Antimicrobial Graphene-Nanoagent for Ultraviolet–Near Infrared Imaging and Photoinactivation

**DOI:** 10.3390/ijms23063230

**Published:** 2022-03-17

**Authors:** Wen-Shuo Kuo, Yen-Sung Lin, Ping-Ching Wu, Chia-Yuan Chang, Jiu-Yao Wang, Pei-Chi Chen, Miao-Hsi Hsieh, Hui-Fang Kao, Sheng-Han Lin, Chan-Chi Chang

**Affiliations:** 1 School of Chemistry and Materials Science, Nanjing University of Information Science and Technology, Nanjing 210044, China; wskuo88@gmail.com; 2 State Key Laboratory for Chemistry and Molecular Engineering of Medicinal Resources, Guangxi Normal University, Guilin 541004, China; 3 Center for Allergy, Immunology and Microbiome (AIM), China Medical University Children’s Hospital, China Medical University, Taichung 404, Taiwan; a122@mail.ncku.edu.tw (J.-Y.W.); simple48686@gmail.com (P.-C.C.); karinadrift@gmail.com (M.-H.H.); 4 Division of Pulmonary and Critical Care Medicine, An Nan Hospital, China Medical University, Tainan 709, Taiwan; chestlin@gmail.com; 5 Department of Nursing, Chung Hwa University of Medical Technology, Tainan 717, Taiwan; 6 Department of Biomedical Engineering, National Cheng Kung University, Tainan 701, Taiwan; wbcxyz@bme.ncku.edu.tw; 7 Department of Mechanical Engineering, National Cheng Kung University, Tainan 701, Taiwan; cychang0829@gs.ncku.edu.tw; 8 Department of Nursing, National Tainan Junior College of Nursing, Tainan 700, Taiwan; kaohuif@gmail.com; 9 Department of Anesthesiology, E-Da Hospital, Kaohsiung 824, Taiwan; 10 Department of Otolaryngology, National Cheng Kung University Hospital, College of Medicine, National Cheng Kung University, Tainan 70456, Taiwan

**Keywords:** sorted-graphene quantum dot, excitation-wavelength-independent photoluminescence, two-photon photoinactivation, near-infrared-II two-photon bioimaging, multi-drug resistant microbe

## Abstract

Nitrogen doping and amino group functionalization through chemical modification lead to strong electron donation. Applying these processes to a large *π*-conjugated system of graphene quantum dot (GQD)-based materials as electron donors increases the charge transfer efficiency of nitrogen-doped amino acid-functionalized GQDs (amino-N-GQDs), resulting in enhanced two-photon absorption, post-two-photon excitation (TPE) stability, TPE cross-sections, and two-photon luminescence through the radiative pathway when the lifetime decreases and the quantum yield increases. Additionally, it leads to the generation of reactive oxygen species through two-photon photodynamic therapy (PDT). The sorted amino-N-GQDs prepared in this study exhibited excitation-wavelength-independent two-photon luminescence in the near-infrared region through TPE in the near-infrared-II region. The increase in size resulted in size-dependent photochemical and electrochemical efficacy, increased photoluminescence quantum yield, and efficient two-photon PDT. Therefore, the sorted amino-N-GQDs can be applicable as two-photon contrast probes to track and localize analytes in in-depth two-photon imaging executed in a biological environment along with two-photon PDT to eliminate infectious or multidrug-resistant microbes.

## 1. Introduction

Graphene quantum dot (GQD)-based materials with *π*–*π* configurations and surface groups have a high surface area, large diameter, and excellent surface grafting. These materials may cause intrinsic–state and defect-state emission to achieve photoluminescence (PL). Intrinsic–state emission is induced by the quantum size effect, zigzag edge sites, or the recombination of localized electron–hole pairs; by contrast, defect-state emission originates from defect effects (energy traps) [1,2]. The PL emission of a material determines its suitability for imaging and photochemistry [3]. GQDs can be bonded with nitrogen atoms (N-GQDs) to alter their chemical composition and modulate their band gap, enhancing their photochemical properties and facilitating the fabrication of tunable luminescence in bioimaging and photodynamic (or photoinactivation) applications [4,5]. In addition, primary amine molecules (also known as amino group–functionalized molecules) can be chemically modified to cause strong electron donation and significantly alter the electronic properties of nitrogen–doped amino acid–functionalized GQD (amino-N-GQD) materials, increasing electrochemical and photochemical activities [6].

Combining multiphoton and near-infrared (NIR) excitation is an effective approach for investigating photoexcitation. This approach involves less absorption and a shorter photoexcitation period than other types of excitation. Moreover, this approach involves ultra-low energy consumption. These attributes enable the deep penetration of biological specimens and effective observation [7]. This study used a novel inverted optical microscopy system with a femtosecond Ti-sapphire laser with a repetition rate of 80 MHz and optical parametric oscillators (Mai Tai Spectra-Physics, Santa Clara, CA, USA; Scheme S1). The derived amino-N-GQDs with quantum confinement in the sp^2^ domain and intrinsic–state and defect–state emissions can cause an excitation wavelength–dependent PL phenomenon. Therefore, the amino-N-GQDs were sieved through membranes with pores of various sizes to ensure that they exhibited homogeneous atom doping functionalities, which enabled the investigation of electronic and intrinsic properties related to optical behavior under quantum confinement effects [8,9]. This phenomenon results in excitation wavelength-independent two-photon luminescence (EWI-TPL) emission at a two-photon excitation (TPE) wavelength of 960 nm in the NIR-II region [7,10]. X-ray photoelectron spectroscopy (XPS) revealed the enlargement of the sorted amino-N-GQDs, which increased the number of C–N groups and pyridinic-, amino-, and pyrrolic-N functionalities. This increase could induce a radiative recombination of localized electron-hole pairs, resulting in significant two-photon properties, including favorable two-photon absorption (TPA), high TPL emission, excellent absolute TPE cross-sections, a short lifetime, a high ratio of radiative to nonradiative decay rates, and high post-TPE stability. In addition, the mean lateral size increased, which resulted in a high PL quantum yield (QY) and high efficiency in photodynamic therapy (PDT, or photoinactivation) under TPE [excitation wavelength (Ex): 960 nm; ultralow energy: 222.7 nJ pixel^−1^; photoexcitation period: 100–170 scans; total effective exposure time: 0.65–1.11 s]. The results indicated that the sorted amino-N-GQDs are promising as two-photon contrast probes for tracking and localizing analytes in detail in two-photon imaging (TPI) of a biological environment in two-photon PDT; they can be used to eliminate infectious microbes effectively.

## 2. Results

Amino-N-GQDs were synthesized from graphene oxide sheets through ultrasonic shearing according to the modified Hummers’ method (Figure S1, Table S1, and Scheme S2a) [11]. XPS indicated that the as-prepared amino-N-GQDs with homogeneous O and N distributors exhibiting high crystallinity and uniformity were sieved through membranes with pores of various sizes (Figures S2a–d, S3 and S4). Low-magnification and high-resolution transmission electron microscopy (HR-TEM; inset images in Figure S2a–d) were used to characterize the amino-N-GQDs. The mean lateral sizes of the sorted dots were set to 9.1 ± 0.2 nm (amino-N-GQD 9.1), 9.9 ± 0.2 nm (amino-N-GQD 9.9), 11.1 ± 0.3 nm (amino-N-GQD 11.1), and 12.0 ± 0.3 nm (amino-N-GQD 12.0). Further characterization results indicated that the sorted amino-N-GQDs were successfully prepared (Figures S3–S5).

**Figure 1:**
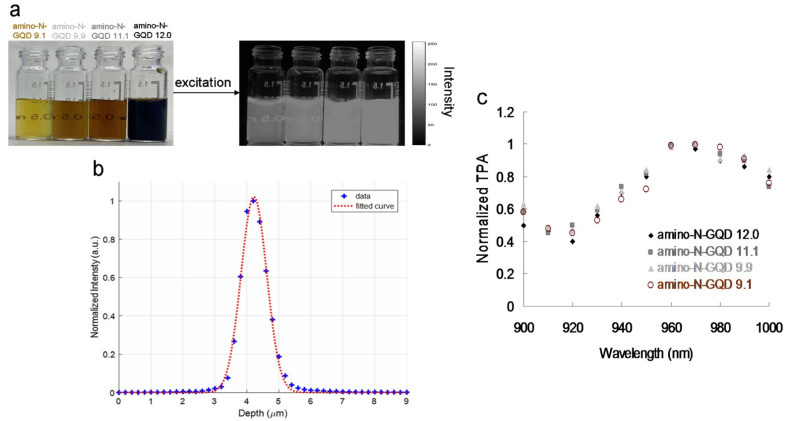
(**a**) Photographs of materials without and with 630 nm (gray-level) light excitation. (**b**) *z*-axis scan of thin gold film for measuring the second harmonic generation signal at various positions. The laser system’s *z*-axis resolution (full width at half maximum, FWHM) was 0.90 μm (fit using the Gaussian function). (**c**) Relative TPA spectra of the sorted amino-N-GQDs. TPE signals were obtained at 900–1000 nm and at 127.3 nJ pixel^−1^. Delivered dose: 0.75 μg mL^−1^ material.

**Figure 2:**
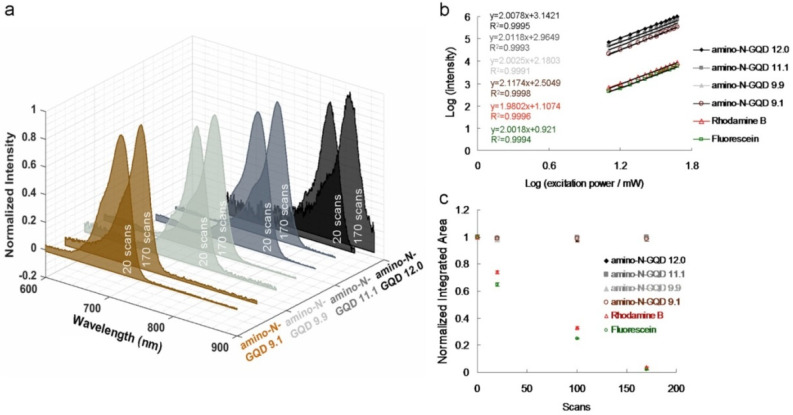
(**a**) Relative TPL spectra of materials at a TPE power of 222.7 nJ pixel^−1^ [20 and 170 scans (total effective exposure times: ~0.13 and 1.11 s), respectively; cut off = 900 nm, determined using cascading filters]. (**b**) TPL intensity dependence on the excitation power (logarithm) of materials and fluorophores; the slope is approximately 2.00 ± 0.02. TPE power = 1272.8–5091.2 nJ pixel^−1^; *R*^2^ > 0.999. (**c**) Two-photon stability of the amino-N-GQDs, rhodamine B, and fluorescein at a TPE power of 222.7 nJ pixel^−1^ with 20, 100, and 170 scans. The normalized integrated area was calculated by dividing the emission intensities of the integrated area after photoexcitation by those of the newly prepared material without photoexcitation. Delivered dose: 0.75 μg mL^−1^ material. Data are presented as means ± standard deviations (*n* = 6).

Zigzag edge sites, localized electron–hole pairs, and quantum effects have been used to induce intrinsic-state emission in GQD-based materials; however, defect effects (energy traps) have been used to trigger defect-state emission [2,6,7]. To demonstrate such effects, Figure 1a displays the sorted amino-N-GQD dispersions, various levels of PL emission (gray-level images), dots with slight variations in size, and wavelengths at 630 nm encompassing the NIR-I window. The *x*–*y* focal point and *z*-axis resolution (full width at half maximum, FWHM) of the laser system were set to approximately 0.45 and 0.90 μm, respectively (Figure 1b). Satisfactory TPA in the NIR-II window was measured in subsequent experiments using the custom femtosecond Ti-sapphire laser optical system displayed in Scheme S1 (for details on the system, please refer to the Materials and Methods section), with an extension of approximately 960 nm (Figure 1c). Applying the most effective excitation wavelength can significantly enhance the two-photon properties of the materials used for bioimaging with TPEs [12]. Figure 2a shows the TPL spectra of the increase in the size of the sorted amino-N-GQDs, indicating red-shifted peaks of amino-N-GQD 9.1, amino-N-GQD 9.9, amino-N-GQD 11.1, and amino-N-GQD 12.0 at approximately 719, 772, 810, and 862 nm, respectively, in the NIR region under TPE (power: 222.7 nJ pixel^−1^; scans: 20 or 170; total effective exposure time: approximately 0.13 or 1.11 s; Ex: 960 nm). The emission peaks determined via PL spectrophotometry for amino-N-GQD 9.1, amino-N-GQD 9.9, amino-N-GQD 11.1, and amino-N-GQD 12.0 were observed at approximately 719, 772, 810, and 862 nm, respectively, and they exhibited EWI-PL features (Figure S6). XPS revealed that the electron redistribution increased as the number of carbonyl groups increased (Figure S3), which decreased the energy gaps, resulting in TPL red shifts [13]. The quadratic dependence of the TPL increases with TPE power during this process [14]. Figure 2b demonstrates the existence of a two-photon process with an exponent of 2.00 ± 0.02 for sorted dots and conventional fluorophore (e.g., rhodamine B and fluorescein; Figure 2b).

**Figure 3:**
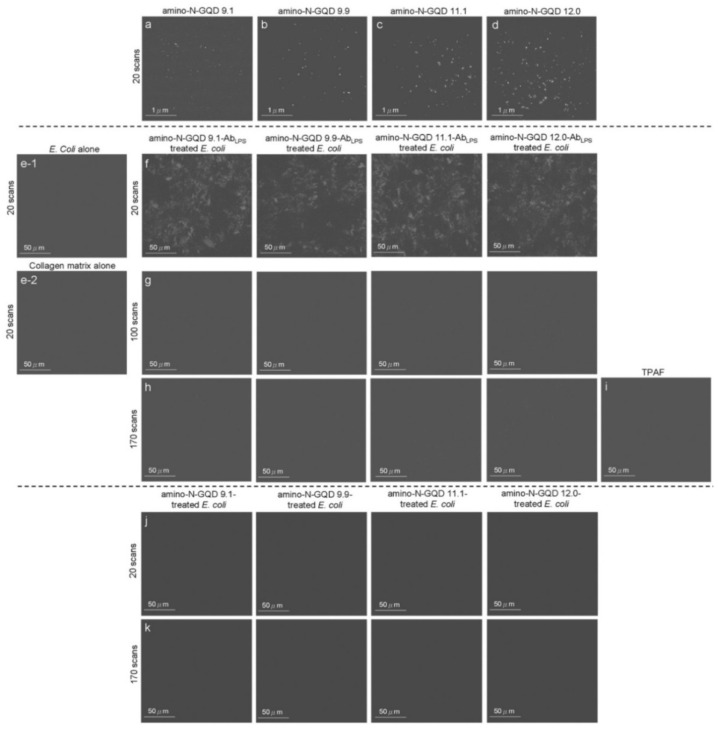
TPL images (gray-level) of the (**a**) amino-N-GQD 9.1, (**b**) amino-N-GQD 9.9, (**c**) amino-N-GQD 11.1, and (**d**) amino-N-GQD 12.0 at a TPE power of 222.7 nJ pixel^−1^ with 20 scans. (**e-1**) *E.* *coli* alone, (**e-2**) collagen matrix alone and bacteria subjected to sorted amino-N-GQD–Ab_LPS_ treatment at a 180 μm depth (222.7 nJ pixel^−1^) with (**f**) 20 scans and (**g**) 100 scans through TPE. (**h**) Images acquired after an additional 170 scans. (**i**) TPAF image of the unlabeled bacteria. TPL images of bacteria treated without the antibody-coated materials with (**j**) 20 scans and (**k**) 170 scans through photoexcitation under the same conditions. All images were acquired after 3 h of additional incubation to make the PDT action effectively. TPE wavelength: 960 nm. Delivered dose (OD_600_): approximately 0.05 of *E. coli* or 0.75 μg mL^−1^ material–Ab_LPS_.

The sorted amino-N-GQDs with homogeneous O and N functionalities can be used to investigate the intrinsic electronic properties related to optical behavior with quantum confinement, leading to EWI-TPL under TPE. The sorted amino-N-GQDs exhibited two-photon stability, which could be attributed to the limited photobleaching due to the post-TPE TPL intensity of the dots (Figure 2c). Rhodamine B and fluorescein’s fluorescence demonstrated low robustness against photobleaching upon TPE exposure (power: 222.7 nJ pixel^−1^; scans: 20, 100, or 170; total effective exposure time: approximately 0.13, 0.65, or 1.11 s). Ultraviolet photoelectron spectroscopy revealed *n*–state levels that were maintained at approximately the same energetic positions (6.6–6.8 eV; Figure S7), regardless of the size determined through TEM and Raman spectroscopy (Figures S2 and S5), confirming the highest occupied orbital level of the sorted dots. The quantum confinement resulting from the particle size regulates the wavelengths of radiative transitions. The EWI-TPL emissions from the sorted amino-N-GQDs indicate the absence of trap states between the *n*-state and π* energy levels. A change in particle size did not cause any disturbance at the *n*-state level. The EWI-TPL of the sorted dots could be attributed to π*→*n* recombination, which triggers electron transition and phonon scattering. Measurements revealed that the absolute fluorescence QY [15] of the materials ranged from 0.39 (for amino-N-GQD 9.1) to 0.48 (for amino-N-GQD 12.0); these values are higher compared to those documented in other studies [16,17]. Desirable yields were achieved because of the electron-donating species in the sorted amino-N-GQD structure. XPS revealed that many C–N configurations functioned as electron-donating species and increased the material QY through the inhibition of nonradiative transitions (Figure S3). However, the low QY was because of the large number of electron-withdrawing carbonyl functional groups acting as non-radiative trap centers (Figure S3). Characterization of the sorted amino-N-GQDs revealed that the successfully prepared GQDs exhibit EWI-TPL characteristics. However, a large cross-section is typically preferred for monitoring molecular actions. The sorted amino-N-GQDs exhibited a large absolute TPE cross-section, ranging from 55,946 to 60,728 Goeppert-Mayer (GM) units (1 GM = 10^−50^ cm^4^ s photon^−1^), which was more than 2900 times the magnitude of fluorescein (~19.2 GM; Table 1). The absolute TPE cross-sections of the amino-N-GQD 9.1, amino-N-GQD 9.9, amino-N-GQD11.1, and amino-N-GQD12.0 were approximately 55,946, 57,332, 59,051, and 60,728 GM, respectively (Table 2; for detailed calculations, please refer to the Materials and Methods section). This difference indicates a high ratio of energy absorption to energy input in the biospecimens. This phenomenon is highly favorable for TPI [18]. Moreover, the TPI emissions of the sorted dots (Figure 3a–d) occurred on a surface through the two-photon process, as shown in Figure 2b (power: 222.7 nJ pixel^−1^; scans: 20; total effective illumination: ~0.13 s; Ex: 960 nm; scan rate: 6.53 ms scan^−1^; scan area: 200 × 200 μm^2^; for details regarding the calculation, see the Materials and Methods).

**Figure 4:**
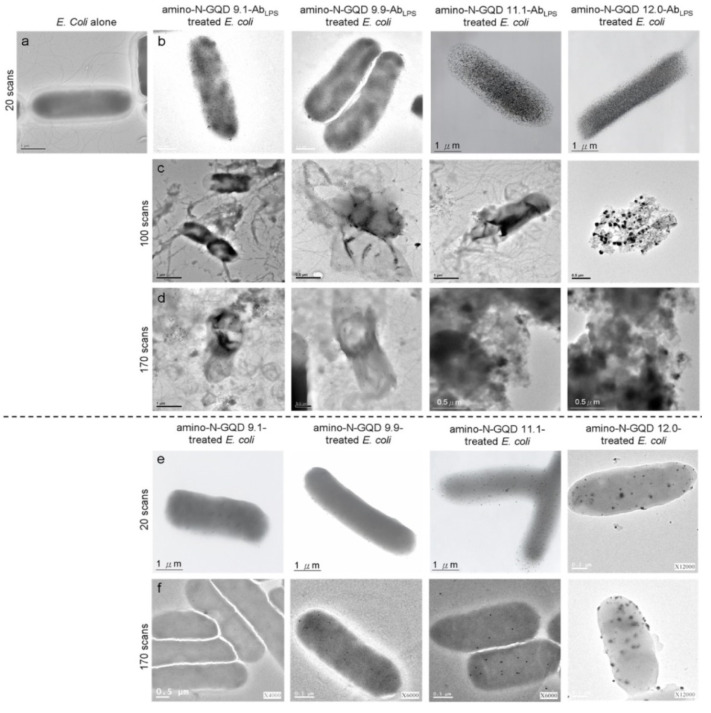
TEM images of the (**a**) bare *E.* *coli* (with 20 scans), (**b**–**d**) sorted amino-N-GQD–Ab_LPS_-treated *E. coli* (with 20, 100, and 170 scans), and (**e**,**f**) sorted amino-N-GQD-treated *E. coli* (with 20 and 170 scans) under TPE (222.7 nJ pixel^−1^). All images were acquired after 3 h of additional incubation. TPE wavelength: 960 nm. Delivered dose (OD_600_): approximately 0.05 of *E. coli* or 0.75 μg mL^−1^ material–Ab_LPS_.

Because the inverted optical microscopy system was not suitable for investigating in vivo assay processes, the biological environment was mimicked by embedding an *Escherichia coli* (*E. coli*; 3.98 ± 1.37 μm in length and 0.98 ± 0.34 μm in width, calculated from 400 counts of bacteria) strain in a collagen matrix [19]. The TPI action occurred at a specimen depth of 180 μm under TPE (power: 222.7 nJ pixel^−1^; scans: 20; total effective illumination: ~0.13 s; Ex: 960 nm; Figure 3e,f). Bacteria were observed under TEM (Figure 4a), but they were undetectable in the TPL images (Figure 3(e-1)), similar to the collagen matrix (Figure 3(e-2)). Lipopolysaccharides (LPSs) are major components of the outer membrane of *E. coli*. The physiologically stable and biocompatible sorted-amino-N-GQDs (Table S2 and Figure S8; a selected concentration of 0.75 μg mL^−1^ material was used in sequential experiments conducted in the dark) were coated with anti-LPS antibody (Ab_LPS_) through electrostatic interaction to increase efficiency and specificity (Scheme S2b). This resulted in the absorption of a substantial amount of sorted dot–Ab_LPS_ on the surface of the bacteria. No exceptional morphology (Figure 4b) was observed on the surface of the bacteria. In contrast, when the GQD size was increased using amino-N-GQD 9.1, amino-N-GQD 9.9, amino-N-GQD11.1, and amino-N-GQD12.0, a high fluorescence QY and large cross-section was detected in the TPL images (Figure 3f). However, the bacteria treated with the photoexcited material–Ab_LPS_ hybrid were severely damaged when the power was increased to 222.7 nJ pixel^−1^ with 100 or 170 scans (with a total effective illumination of ~0.65 or 1.11 s), which resulted in abnormal morphology, as observed through TEM (Figure 4c,d). TPL decreased after 100 scans (Figure 3g) and was undetectable after 170 scans (Figure 3h) at a depth of 180 μm. For unlabeled bacteria, two-photon autofluorescence (TPAF) was not observed for the intrinsic fluorophores under TPE at the same power (Figure 3i). By contrast, TEM images revealed limited attachment and nonspecific binding for the surface of the *E. coli* strain (without antibody coating) treated with the sorted dots (power: 222.7 nJ pixel^−1^; scans: 20; Ex: 960 nm; Figure 4e). Subsequently, TPI revealed almost no TPL emission at 180 μm (Figure 3j). Therefore, the *E. coli* strain treated with the photoexcited sorted dots exhibited a normal morphology even after photoexcitation (power: 222.7 nJ pixel^−1^; scans: 170; Ex: 960 nm; Figure 4f). Under the same conditions, a clear TPI without TPL emission was observed for bacteria without the antibody-coated materials (Figure 3k). However, the images captured at a depth of >180 μm contained spherical aberrations, which severely degraded the image quality. Such aberrations were caused by the mismatch between the refractive indices of the aqueous sample and the maximum *z*-depth of the optical laser system, as well as the influence of the set objective, detection efficiency, and maximum *z-depth* of the optical laser system [20]. Therefore, TPI was not detected at a depth of 200 μm for all the sorted dots (Figure S9). In this study, the maximum *z-depth* for the detection of TPL emission with the laser optical system was 180 μm. This can be attributed to the detection efficiency and set objective, which was set to the depth to obtain the optimal resolution for examining the amino-N-GQDs used as two-photon contrast probes, particularly the sorted amino-N-GQDs with a large lateral size.

The changes in bacterial cell walls and oxidation were examined. The deterioration of the surrounding biological surface substrates was attributed to the reactive oxygen species (ROS) observed through PDT under TPE. These changes could cause bacterial atrophy, morphological damage, and distortion (Figure 4c,d) because of amino-N-GQD desorption from the bacterial surface (Figure 3g,h). The LIVE/DEAD kit was used to investigate the green fluorescence of the living bacteria and an additional incubation time of 3 h was necessary to induce PDT action effectively, leading to the elimination of bacteria (amino-N-GQD 12.0 was used to conduct this experiment; viability > 99%; Figure S10a). The results indicated that the bacteria were almost completely undamaged by exposure to laser treatment (power: 222.7 nJ pixel^−1^; scans: 170; total effective illumination: ~1.11 s; Ex: 960 nm). The bacteria treated with the photoexcited amino-N-GQD 12.0–Ab_LPS_ hybrid without incubation were also nearly undamaged (viability > 99%; Figure S10b). After 3 h of additional incubation, the same panel was observed, and the results indicated that the dead bacteria were somewhat distinguishable (represented by red fluorescence in Figure S10c). Bacterial viability was then quantified for further antimicrobial testing, which revealed nearly complete elimination of the bacteria treated with the amino-N-GQD 12.0–Ab_LPS_ hybrid (elimination > 99%; Figure S10d, corresponding to Figure 3h and Figure 4d) and the strong antibacterial effect of the amino-N-GQDs in PDT. Thus, no other photochemical activity (e.g., photothermal effect) was observed after photoexcitation. In addition, the bacteria not treated with the antibody–coated materials exhibited almost no antimicrobial effect under similar conditions (Figure S10e–h, corresponding to Figure 3k and Figure 4f). 

**Figure 5:**
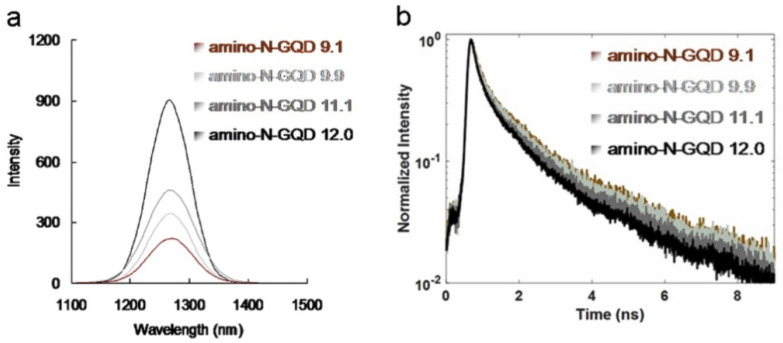
(**a**) Phosphorescence spectra of the sorted amino-N-GQDs (obtained at 1270 nm). (**b**) Decay profiles of the time-resolved room-temperature TPL material. Delivered dose: 0.75 μg mL^−1^ material.

ROS plays a crucial role in PDT by enabling the detection of superoxide anion radicals (O_2_^−^), hydrogen peroxide (H_2_O_2_), and singlet oxygen (^1^O_2_). In PDT, ROS are formed when molecular oxygen reacts with a photoexcited photosensitizer (PS) exposed to a suitable wavelength of light and energy. Photosensitized reactions involving oxygen are categorized as type I or II. A light-sensitized (excited) PS can directly react with a suitable substrate (unsaturated lipids, proteins, or nucleic acids) to produce unstable radicals through proton or electron transfer (type I reaction), leading to oxygenated products in the presence of oxygen, such as O_2_^−^, hydrogen peroxide (H_2_O_2_), or hydroxyl radicals (OH). Subsequently, it reacts with molecular oxygen to form ^1^O_2_ through energy transfer (type II reaction). ROS can induce DNA damage, inactivate enzymes, and oxidize amino acids, causing bacterial injury. However, a considerable amount of ^1^O_2_, O_2_^−^, and H_2_O_2_ was generated, and false-positive ROS signals were observed, which could be due to interactions among the sorted amino-N-GQDs, singlet oxygen sensor green (SOSG), trans-1-(2′-methoxyvinyl)pyrene (*t*-MVP), 2,3-bis (2-methoxy-4-nitro-5-sulfophenyl)-2H-tetrazolium-5-carboxanilide (XTT), glutathione (GSH), and 2,7-dichlorodihydrofluorescein diacetate (H_2_DCFDA), which might compromise the results (Tables S3 and S4). The ROS generated by the bacteria treated with the sorted amino-N-GQD–Ab were monitored (Tables S5 and S6), and their signals were consistent with the ^1^O_2_ phosphorescence signals emitted at 1270 nm from the sorted amino-N-GQDs (Figure 5a). The material without antibody coating (Tables S7 and S8) generated less ROS than the Ab_LPS_–coated material. Furthermore, ^1^O_2_ QY (Φ_Δ_) values for amino-N-GQD 9.1, amino-N-GQD 9.9, amino-N-GQD 11.1, and amino-N-GQD 12.0 [21] were approximately 0.26, 0.28, 0.31, and 0.34, respectively. This study demonstrates the antimicrobial potential of the developed materials against *E. coli* in PDT. The bactericidal capability of the dots was investigated at a low dose of 0.75 μg mL^−1^ in the dark (TPE energy: 222.7 nJ pixel^−1^; scans: 20; Ex: 960 nm). No significant difference in viability was observed between the panels (Figure S11a,b, corresponding to Figure 3f and Figure 4b, respectively). After 100 scans, TPE still exhibited no bactericidal effect on the bacteria alone, and without TPE, the material in the panel exhibited considerable biocompatibility with the bacteria treated with the sorted dot–Ab hybrid (Figure S11c,d). However, under TPE, the sorted amino-N-GQDs exhibited excellent bactericidal capability (~89%, 93%, 98%, and 100% elimination for amino-N-GQD 9.1–Ab_LPS_, amino-N-GQD 9.9–Ab_LPS_, amino-N-GQD 11.1–Ab_LPS_, and amino-N-GQD 12.0–Ab_LPS_, respectively, amounting to an approximate 0.90–7.82 log_10_ reduction; Figure S10c,d, corresponding to Figure 3g and Figure 4c). In contrast, the observed bacterial viability was higher for the materials without antibody coating (>98% viability) compared to the materials with the coating (Figure S11c,d). Although antimicrobial capabilities were still not apparent (~6%, 8%, 9%, and 11% elimination for amino-N-GQD 9.1, amino-N-GQD 9.9, amino-N-GQD 11.1, and amino-N-GQD 12.0 without the coating antibody, respectively), the sorted dots exhibited 100% antimicrobial efficacy against all *E. coli* strains treated with the sorted dot–Ab_LPS_ hybrid under TPE at 170 scans (Figure S11e,f, corresponding to Figure 3h and Figure 4d). The surface protein, protein A, on the cell wall of a multidrug-resistant (MDR) strain of gram-positive methicillin-resistant *Staphylococcus aureus* (MRSA) was considered. Thus, the material was coated with Ab_protein A_ to form a material–Ab_protein A_ hybrid that eliminated MRSA (Figures S12 and S13), demonstrating a trend similar to that shown in Figure S11. These results were attributed to the sorted amino-N-GQDs functioning as a two-photon PS to generate ROS involved in PDT. These results also demonstrated the effectiveness of the antibody coating in enhancing the functions of the materials. Additionally, the trend of ROS generation in MRSA treated with the sorted dot–Ab_protein A_ hybrid (Tables S9–S12) under TPE was similar to that of ROS generation in *E. coli* treated with the material–Ab_LPS_ hybrid (Tables S3–S8).

Amino-N-GQDs exhibited remarkable quantum confinement, and their edge effects could be altered to increase their electrochemical, electrocatalytic, and photochemical activities [6,9]. Strong electron donation and large *π*-conjugated systems increased the charge transfer efficiency of the amino-N-GQDs [22], which resulted in favorable TPA, post-TPE stability, TPE cross-sections, and TPL. Additionally, they increased the ratio of radiative to non-radiative decay rates (amino-N-GQD 9.1: 0.64; amino-N-GQD 9.9: 0.69; amino-N-GQD 11.1: 0.82; and amino-N-GQD 12.0: 0.92; please refer to the Materials and Methods section for the calculation; Table 2). The results indicated that the material passed mainly through the radiative pathway as the fluorescence QY increased (amino-N-GQD 9.1: 0.39; amino-N-GQD 9.9: 0.41; amino-N-GQD 11.1: 0.45; and amino-N-GQD 12.0: 0.48) and the lifetime decreased (from 1.13 to 0.93 ns; Figure 5b, Table 2 and Table 3). Radiative electron–hole pair recombination was observed and it was induced by N dopants and amino groups on the surface of the GQD-based material, which increased the intrinsic-state emission. However, for N dopants and amino groups, the presence of edge amine groups can increase the maximum occupied molecular orbital energy of the graphene flakes [23]. Thus, the narrowing of the orbital band gap, which increased the PL QY, could be attributed to the resonance between the delocalized *π* orbitals and the molecular orbital of the primary amine. XPS revealed that the C–O, C=O, and amide groups, which induced the nonradiative recombination of localized electron–hole pairs and prevented intrinsic–state emission [24], were favorable for small materials (Figures S3 and S4). The PL QY increased with increasing particle size. In addition, chemical modifications strongly affect the electronic properties of the amino-N-GQDs, enabling strong electron donation in primary amine molecules, which is also known as amino group functionalization. Singlet-triplet splitting of the amino-N-GQDs resulted in intersystem crossing and a high triplet-state yield. This splitting process was efficient, and it could compete with the process of internal conversion between multiple identical states, resulting in the creation of ROS for involvement in PDT [9,22]. As the number of edge sites increased, the number of C–N, pyridinic-, amino-, and pyrrolic-N groups increased (Figures S3 and S4). Similarly, as the size of the amino-N-GQDs (Figures S3 and S4) increased (Figures S2 and S5), their antibacterial ability and the number of ROS generated increased (Figure 5a and Tables S3–S8), leading to a highly efficient PDT process.

## 3. Materials and Methods

### 3.1. TEM Observation of the Negatively Stained Bacteria

Bacteria were picked from colonies and suspended in a 1% aqueous sodium phosphotungstate solution (Sigma Aldrich Co., St Louis, MO, USA) at pH 7.0. Droplets of the suspensions were allowed to dry on grids coated with the Formvar. Thereafter, the samples were subjected to TEM.

### 3.2. Molecular Weight of the Sorted-Amino-N-GQDs

The theoretical diameter of benzene is 0.243 nm with a molecular weight of 72 (ignoring the H atoms). According to Figure S2, the mean lateral sizes of the sorted-amino-N-GQDs were approximately 9.1 ± 0.2 nm (amino-N-GQD 9.1), 9.9 ± 0.2 nm (amino-N-GQD 9.9), 11.1 ± 0.3 nm (amino-N-GQD 11.1), and 12.0 ± 0.3 nm (amino-N-GQD 12.0). For the sorted-amino-N-GQDs, assuming there was no leakage from a layer of material and ignoring the exposed functional groups, the benzene number and molecular weight could be approximately 1027 and 26,217 g mol^−1^, 1261 and 32,027 g mol^−1^, 1519 and 38,418 g mol^−1^, 1801 and 45,390 g mol^−1^ (Table S14). The following measurement for the cross-section of TPE was performed using the estimated molecular weights.

### 3.3. Femtosecond Laser Optical System for the Measurements of TPA and TPL

A novel inverted optical microscopy system with a femtosecond Ti-sapphire laser [repetition rate: 80 MHz; Mai Tai with optical parametric oscillators, Spectra-Physics, Santa Clara, CA, USA] optical system: an inverted optical microscope (Zeiss, Oberkochen, Germany); an x–y galvanometer scanner (Cambridge, MA, USA); a triple-axis sample-positioning stage (Prior Scientific Instruments Ltd., London, UK); a *z*–axis piezoelectric nano-positioning stage (Mad City Labs, Madison, WI, USA); photomultiplier tubes (Hamamatsu, Shizuoka, Japan); a data acquisition card with a field-programmable gate array module (National Instruments, Austin, TX, USA) (Scheme S1).

All the Materials and Methods used in this study can be found in the Supplementary Materials.

## 4. Conclusions

Nitrogen doping and amino group functionalization, which result in strong electron donation, can be achieved through chemical modifications. Large *π*-conjugated systems of GQD-based materials acting as electron donors can be chemically manipulated with a low TPE energy in a short photoexcitation period to increase the charge transfer efficiency of the sorted amino-N-GQDs. This study used a novel femtosecond Ti-sapphire laser optical system (power: 222.7 nJ pixel^−1^; scans: 100–170; total effective exposure time: ~0.65–1.11 s; excitation wavelength: 960 nm in the NIR-II region) for chemical modification. The sorted amino-N-GQDs exhibited increased TPA, post-TPE stability, TPE cross-sections, and TPL through the radiative pathway. The lifetime and quantum yield of the sorted amino-N-GQDs decreased and increased, respectively. Additionally, the sorted amino-N-GQDs exhibited EWI-PL in the NIR region and generated ROS after the TPE. Increasing the mean lateral size increased the number of C–N, pyridinic–N, amino–N, and pyrrolic–N functionalities, which induced the radiative recombination of localized electron–hole pairs and provided greater PL QY and efficient PDT action through TPE, enabling the sorted amino-N-GQDs to be applied in contrast probes to track and localize analytes in two-photon PDT.

## Figures and Tables

**Table 1:** TPE cross-section of materials at an excitation wavelength of 960 nm. Delivered dose: 0.75 μg mL^−1^ material.

Reference	Integrated Emission Intensity (Counts)		Action Cross-Section (ησ)
Rhodamine B ^a^	53.9		13.4
**Analyte**	**Integrated Emission Intensity (Counts)**	**Absolute Quantum Yield (η)**	**Absolute Cross-Section (σ)**
amino-N-GQD 9.1	87,754	0.39	55,946
amino-N-GQD 9.9	94,550	0.41	57,332
amino-N-GQD 11.1	106,887	0.45	59,051
amino-N-GQD 12.0	117,250	0.48	60,728
Fluorescein	61.1	0.79 ^b^	19.2

^a^ Rhodamine B was selected as a reference to determine the TPE cross-section. The relevant calculations are shown in the Materials and Methods section; ^b^ Forster, L.S.; Livingston, R. The absolute quantum yields of the fluorescence of chlorophyll solutions. *J. Chem. Phys.* **1952**, *20*, 1315–1320.

**Table 2:** Two-photon properties of the sorted amino-N-GQDs. Delivered dose: 0.75 μg mL^−1^ material.

	Absolute QY	Absolute Cross Section of TPE (GM)	Lifetime (ns)	Radiative Decay Rate (×10^8^ s^−1^)	Nonradiative Decay Rate (×10^8^ s^−1^)	Ratio of Radiative to Nonradiative Decay Rates
amino-N-GQD 9.1	0.39	55,946	1.13	3.45	5.40	0.64
amino-N-GQD 9.9	0.41	57,332	1.09	5.41	5.30	0.69
amino-N-GQD 11.1	0.45	59,051	1.04	4.33	5.29	0.82
amino-N-GQD 12.0	0.48	60,728	0.93	5.16	5.59	0.92

**Table 3:** Lifetime data and parameter generated using a time-correlated single-photon counting technique involving a triple-exponential fitting function while monitoring the emission under TPE. Delivered dose: 0.75 μg mL^−1^ material.

	3 Exp Fitting Model: (a0 × exp(a1x) + a2 × exp(a3x) + a4 × exp(a5x) + a6)							Lifetime 1	Lifetime 2	Lifetime 3	Average Lifetime (ns)
	a0	a1	a2	a3	a4	a5	a6				
amino-N-GQD 9.1	564.38	5.77	701.92	−0.97	187.90	−0.23	−0.53	0.17	1.03	4.39	1.13
amino-N-GQD 9.9	583.76	5.76	770.12	1.08	256.81	0.27	4.23	0.17	0.92	3.69	1.09
amino-N-GQD 11.1	668.75	6.00	821.54	1.07	255.08	0.27	3.85	0.17	0.94	3.64	1.04
amino-N-GQD 12.0	1747.39	6.20	1948.88	1.12	512.34	0.27	4.73	0.16	0.89	3.71	0.93

## Data Availability

Not applicable.

## Supplementary Materials

The following supporting information can be downloaded at: https://www.mdpi.com/article/10.3390/ijms23063230/s1.

## References

[1] Gong P., Sun L., Wang F., Liu X., Yan Z., Wang M., Zhang L., Tian Z., Liu Z., You L. (2019). Highly fluorescent N-doped carbon dots with two-photon emission for ultrasensitive detection of tumor marker and visual monitor anticancer drug loading and delivery. Chem. Eng. J..

[2] Sun Z., Fang S., Hu Y.H. (2020). 3D graphene materials: From understanding to design and synthesis control. Chem. Rev..

[3] Lee J., Wong D., Velasco J., Rodriguez-Nieva J.F., Kahn S., Tsai H.Z., Taniguchi T., Watanabe K., Zettl A., Wang F. (2016). Imaging electrostatically confined Dirac fermions in graphene quantum dots. Nat. Phys..

[4] Sun L., Luo Y., Li M., Hu G., Xu Y., Tang T., Wen J., Li X., Wang L. (2017). Role of pyridinic-N for nitrogen-doped graphene quantum dots in oxygen reaction reduction. J. Colloid Interface Sci..

[5] Li M., Wu W., Ren W., Cheng H.M., Tang N., Zhong W., Du Y. (2012). Synthesis and upconversion luminescence of N-doped graphene quantum dots. Appl. Phys. Lett..

[6] Torres T. (2012). Graphene chemistry. Chem. Soc. Rev..

[7] Zhao G., Li X., Huang M., Zhen Z., Zhong Y., Chen Q., Zhao X., He Y., Hu R., Yang T. (2017). The physics and chemistry of graphene-on-surfaces. Chem. Soc. Rev..

[8] Yildirim M., Sugihara H., So P.T.C., Sur M. (2019). Functional imaging of visual cortical payers and subplate in awake mice with optimized three-photon microscopy. Nat. Commun..

[9] Wang X., Li X., Zhang L., Yoon Y., Weber P.K., Wang H., Guo J., Dai H. (2009). N-doping of graphene through electrothermal reactions with ammonia. Science.

[10] Kim L., Kim S., Jha P.K., Brar V.W., Atwater H.A. (2021). Mid-infrared radiative emission from bright not plasmons in graphene. Nat. Mater..

[11] Hummers W.S., Offeman R.E. (1958). Preparation of graphitic oxide. J. Am. Chem. Soc..

[12] Zhao W., Li Y., Yang S., Chen Y., Zheng J., Liu C., Qing Z., Li J., Yang R. (2016). Two-photon excitation/red emission, ratiometric fluorescent nanoprobe for intracellular pH imaging. Anal. Chem..

[13] Liu J., Li D., Zhang K., Yang M., Sun H., Yang B. (2018). One-step hydrothermal synthesis of nitrogen-doped conjugated carbonized polymer dots with 31% efficient red emission for in vivo imaging. Small.

[14] Horton N.G., Wang K., Kobat D., Clark C.G., Wise F.W., Schaffer C.B., Xu C. (2013). In vivo three-photon microscopy of subcortical structures within an intact mouse brain. Nat. Photonics.

[15] Würth C., Grabolle M., Pauli J., Spieles M., Resch-Genger U. (2013). Relative and absolute determination of fluorescence quantum yields of transparent samples. Nat. Protoc..

[16] Li B., Wu C., Wang M., Charan K., Xu C. (2020). An adaptive excitation source for high-speed multiphoton microscopy. Nat. Methods.

[17] Zhang F., Liu F., Wang C., Xin X., Liu J., Guo S., Zhang J. (2016). Effect of lateral size of graphene quantum dots on their properties and application. ACS Appl. Mater. Interfaces.

[18] We J., Qiu J., Ren L., Zhang K., Wang S., Weeks B. (2014). Size sorted multicolor fluorescence graphene oxide quantum dots obtained by differential velocity centrifugation. Sci. Adv. Mater..

[19] Roy B., Yuan L., Lee Y., Bharti A., Mitra A., Shivashankar G.V. (2020). Fibroblast rejuvenation by mechanical reprogramming and redifferentiation. Proc. Natl. Acad. Sci. USA.

[20] Richardson D.S., Lichtman J.W. (2015). Clarifying tissue clearing. Cell.

[21] Shi L., Hernandez B., Selke M. (2006). Singlet oxygen generation from water-soluble quantum dot-organic dye nanocomposites. J. Am. Chem. Soc..

[22] Son D.T., Kwon B.K., Park D.H., Seo W.S., Yi Y., Angadi B., Lee C.L., Choi K.W. (2012). Emissive ZnO-graphene quantum dots for white-light-emitting diodes. Nat. Nanotechnol..

[23] Tetsuka H., Asahi R., Nagoya A., Okamoto K., Tajima I., Ohta R., Okamoto A. (2012). Optically tunable amino-functionalized graphene quantum dots. Adv. Mater..

[24] Bao L., Zhang Z.L., Tian Z.Q., Zhang L., Liu C., Lin Y., Qi B., Pang D.W. (2011). Electrochemical tuning of luminescent carbon nanodots: From preparation to luminescence mechanism. Adv. Mater..

