# Supplementary material for: Two-Photon–Near Infrared-II Antimicrobial Graphene-Nanoagent for Ultraviolet–Near Infrared Imaging and Photoinactivation
Source: Int J Mol Sci. 2022 Mar 17;23(6):3230. doi: 10.3390/ijms23063230 (PMC8949782; doi:10.3390/ijms23063230)
Supplement: Supplementary file 1 [file ijms-23-03230-s001.zip › ijms-1579492 supplementary material.pdf]

Supplementary Material

Two-Photon–Near Infrared-II Antimicrobial Graphene–Nanoagent for Ultraviolet–Near Infrared Imaging and Photoinactivation

Wen-Shuo Kuo [1,2,3,†], Yen-Sung Lin[4,5,†], Ping-Ching Wu [6], Chia-Yuan Chang [7], Jiu-Yao Wang [3], Pei-Chi Chen [3], Miao-Hsi Hsieh [3], Hui-Fang Kao [8], Sheng-Han Lin [9,*] and Chan-Chi Chang [10,*]

[1] School of Chemistry and Materials Science, Nanjing University of Information Science and Technology, Nanjing 210044, Jiangsu, China; wskuo88@gmail.com
[2] State Key Laboratory for Chemistry and Molecular Engineering of Medicinal Resources, Guangxi Normal University, Guilin 541004, Guangxi, China
[3] Center for Allergy, Immunology and Microbiome (AIM), China Medical University Children's Hospital, China Medical University Hospital, China Medical University, Taichung 404, Taiwan; a122@mail.ncku.edu.tw (J.-Y.W.); simple48686@gmail.com (P.-C.C.); karinadrift@gmail.com (M.-H.H.)
[4] Division of Pulmonary and Critical Care Medicine, An Nan Hospital, China Medical University, Tainan 709, Taiwan; chestlin@gmail.com
[5] Department of Nursing, Chung Hwa University of Medical Technology, Tainan 717, Taiwan
[6] Department of Biomedical Engineering, National Cheng Kung University, Tainan 701, Taiwan; wbcxyz@bme.ncku.edu.tw
[7] Department of Mechanical Engineering, National Cheng Kung University, Tainan 701, Taiwan; cychang0829@gs.ncku.edu.tw
[8] Department of Nursing, National Tainan Junior College of Nursing, Tainan 700, Taiwan; kaohuif@gmail.com
[9] Department of Anesthesiology, E-Da Hospital, Kaohsiung 824, Taiwan
[10] Department of Otolaryngology, National Cheng Kung University Hospital, College of Medicine, National Cheng Kung University, Tainan 70456, Taiwan
* Correspondence: ed111667@edah.org.tw (S.-H.L.); 109a0015@gs.ncku.edu.tw (C.-C.C.)
† The authors contributed equally to this work.

Materials and Methods

Preparation of graphene quantum dots (GQDs) doped with nitrogen and functionalized with an amino group (amino-N-GQDs) and sorted amino-N-GQDs **[1]**

Amino-N-GQDs: graphene oxide was prepared using a natural graphite powder (Bay carbon Inc., Bay City, MI, USA) through a modified Hummers' method. Graphite (8.5 M) and $NaNO_3$ (0.6 M) (Merck & Co., Kenilworth, NJ, USA) were mixed with H_2SO_4 (18M; Sigma Aldrich Co., St Louis, USA). $KMnO_4$ (2.0 M; FUJIFILM Wako Chemicals USA Inc., Richmond, VA, USA) was slowly added with continual stirring at 35 °C overnight. Subsequently, ddH_2O was gradually added and continually stirred. H_2O_2 (35 %; Sigma Aldrich Co., St Louis, MO, USA) was added to terminate the reaction. Washing and centrifugation with ddH_2O were performed several times, and the graphene oxide was collected. The as-prepared graphene oxide was placed in a tube furnace and heated to 400–600 °C in the presence of $NH_{3(g)}$ for 4–6 h; it was subsequently introduced to concentrated HNO_3 (16.0 M; Sigma Aldrich Co., St Louis, MO, USA) and stirred for 2 days. The mixture was placed in a water bath sonicator for 2 days and maintained the temperature of water was approximately 45 °C; subsequently placed in an oven at 160 °C for 1 day to vaporize all the liquid. Washing and centrifugation (83000 rpm, > 10 min; Optima TLX Ultracentrifuge, Optima TLX Ultracentrifuge, Beckman Coulter Inc., Brea, CA, USA) with ddH_2O were conducted several times. The supernatant was filtered through a 0.22 μm microporous membrane. The pH (7.4) of the resulting black suspension was tuned with NaOH (1M; Sigma Aldrich Co., St Louis, MO, USA), and it was stayed in a dialysis bag (retained molecular weight: 300 kDa) >12 h to obtain the N-GQDs specimens. The as-prepared N-GQDs were mixed with $NH_{3(aq)}$ (28 %; Sigma Aldrich Co., St Louis, MO, USA), stored in a Teflon-lined stainless steel autoclave, and reacted at 180 °C for 5 h. The resulting mixture was washed with ddH_2O, centrifuged several times, and subsequently dried in an oven at 50 °C overnight to obtain the amino-N-GQDs specimens (**Scheme S2a**).

Sorted-amino-N-GQDs: the amino-N-GODs were sieved using centrifugation tubes equipped with polypropylene membranes filters with cut-off molecular weights of 300, 100, and 50 kD (Sartorius, Göttingen, Germany). The amino-N-GOD suspension was flowed through membranes arranged in a sequence of decreasing pore size and collected at serial stages to form the sorted amino-N-GQDs of varying sizes, yielding mean lateral sizes of 9.1±0.2 nm (amino-N-GQD 9.1), 9.9±0.2 nm (amino-N-GQD 9.9), 11.1±0.3 nm (amino-N-GQD 11.1), and 12.0±0.4 nm (amino-N-GQD 12.0) (**Figure S2**).

A 0.75 mg mL^{-1} or 7.5 mg mL^{-1} stock solution of materials was prepared for the following experiments.

Characterization

Droplets of materials were allowed to dry on grids coated with Formvar. Materials were subject to transmission electron microscope (TEM, JEOL, Akishima, Tokyo, Japan) observation. The height profile diagram, thickness and size of samples were determined by Atomic force microscopy (AFM, multimode 8, Bruker, Billerica, MA, USA). The crystalline structures of samples were identified using X-ray diffraction (XRD, Bruker, Billerica, MA, USA) with Cuκα radiation at 30 kv and 30 mA. Fourier transform infrared (FTIR), ultraviolet-visible (UV-vis), zeta potential and dynamic light scattering (DLS) spectra of samples

were recorded by the spectrometers: PerkinElmer RX1 (PerkinElmer, Waltham, MA, USA), U-4100 Hitachi (Chiyoda-ku, Tokyo, Japan) and Manern Nano-ZS90 (Worcestershire, West Midlands, UK), respectively. Raman spectroscopy (Thermo Fisher Scientific, Waltham, MA, USA) was used to examine the crystallinity of samples with 532 nm laser. X-ray photoelectron spectroscopy (XPS) (VersaProbe, Chanhassen, MN, USA) was employed to examine the surface chemistry of the materials, the O(1s)/C(1s) and N(1s)/C(1s) atomic ratios of materials. The photoluminescence (PL) signal was recorded by the spectrophotometer (F-7000, Hitachi, Chiyoda-ku, Tokyo, Japan).

Bacterial cultures

a) For *Escherichia coli* (*E. coli*, ATCC 25922; obtained from our own laboratory):

LB agar plates: Add 10 g of tryptone, 5 g of yeast extract, 15 g of agar (BD, Franklin Lakes, NJ, USA) and 8 g of NaCl (Sigma Aldrich Co., St Louis, MO, USA) to ddH_2O in a 2L flask. Add a stir bar to the flask and then place the flask on a stir plate to mix. Bring volume up to 1L using ddH_2O. Loosely cover the top of the flask with aluminum foil and then autoclave the media on liquid cycle. Once sterilized, place the flask on a stir plate until the media has cooled to 65 °C. Allow agar to solidify at room temperature for at least 1 day then store the plates, top down, in plastic bags at 4 °C.

LB broth: Add 10 g of tryptone, 5 g of yeast extract (BD, Franklin Lakes, NJ, USA) and 8 g of NaCl (Sigma Aldrich Co., St Louis, MO, USA) to ddH_2O in a 2L flask. Add a stir bar to the bottle and then place the bottle on a stir plate to mix. Bring volume up to 1L using ddH_2O. Once the powder is completely dissolved, remove the stir bar, loosely cap the bottle and autoclave the media on liquid cycle. Once sterilized, the broth may be stored at room temperature or 4 °C.

Thawing frozen *Escherichia coli* (*E. coli*): For frozen cultures, thaw *E. coli* using gentle agitation in a water bath that is set to 25-30°C. Thawing will be rapid; ~2 mins or until all ice crystals have melted. For anaerobic cultures, thaw the vial only in an anaerobic environment; the viability of the cell decrease rapidly if the vial is thawed in an oxygenic environment. Once the vial is thawed, decontaminate the outer surface using 70% ethanol (Sigma Aldrich Co., St Louis, MO, USA). The entire contents of the vial can then be aseptically transferred to a test tube containing 5–6 mL of LB broth. Incubate the culture at 37 °C. Additional test tubes can be inoculated by transferring 0.5 mL of the culture of the primary culture to additional secondary cultures.

Growth of *E. coli* on LB agar plates: Using a sterile applicator streak out a small amount of *E. coli* from the frozen stock onto a small section of a LB agar plate using aseptic technique. With a new sterile applicator, streak out a quadrant *E. coli* by passing through the initial quadrant several times. Repeat this process 1-2 more times, passing a new applicator through most recently streaked quadrant. Incubate the plates for 16-24 h at 37 °C.

Growth of *E. coli* in LB broth: *E. coli* grows rapidly in broth culture at 37 °C with aeration. As such, overnight cultures of *E. coli* are typically started at the end of the day just prior to leaving the LAB using either an isolated colony from an agar plate or a frozen stock. Using septic technique and transfer a single colony of *E. coli* from the streak plate into the aliquoted broth by tilting the culture tube and rubbing the

inoculating loop against the side of the tube at the liquid-air interface. Grow cultures overnight (~16–18 h) at 37 °C with shaking.

Preparation of *E. coli* frozen stocks: Long-term storage of *E. coli* should occur at -80 °C to prevent accumulation of mutations. To prepare a frozen stock of *E. coli* add an aliquot (850 μL) of an overnight culture to sterile dimethyl sulfoxide (DMSO, 150 μL, Sigma Aldrich Co., St Louis, MO, USA) to one sterile cryotube using aseptic technique and vortex / or invert briefly to mix. The addition of DMSO prevents complete freezing of the bacteria and thus limits bacterial damage as result of the transition to -80 °C.

b) For methicillin-resistant *Staphylococcus aureus* (MRSA, ATCC 27659; purchased from Food Industry Research and Development Institute, Taiwan):

Brain Heart Infusion (BHI) agar plates: Add 52 g of BHI agar (Catalog number 241830, DIFCO, BD, Franklin Lakes, NJ, USA) to ddH_2O in a 2L flask. Add a stir bar to the flask and then place the flask on a stir plate to mix. Bring volume up to 1L using ddH_2O. Loosely cover the top of the flask with aluminum foil and then autoclave the media on liquid cycle. Once sterilized, place the flask on a stir plate until the media has cooled to 65 °C. Allow agar to solidify at room temperature for at least 1 d then store the plates, top down, in plastic bags at 4 °C.

BHI broth: Add 37 g of BHI (Catalog number 237500, BACTO, BD, Franklin Lakes, NJ, USA) to ddH_2O in a 2L flask. Add a stir bar to the bottle and then place the bottle on a stir plate to mix. Bring volume up to 1L using ddH_2O. Once the powder is completely dissolved, remove the stir bar, loosely cap the bottle and autoclave the media on liquid cycle. Once sterilized, the broth may be stored at room temperature or 4 °C.

Reviving freeze-dried Reviving freeze-dried MRSA: For freeze dried cultures, using a single tube of the BHI broth (5-6 mL) and withdraw 0.5-1.0 mL with pipette. Use this to rehydrate the entire pipette and transfer the entire suspension back into the broth tube and mix well. The last few drops of this suspension may also be transferred to an agar slant. The anaerobic cultures must be rehydrated only in an anaerobic environment; the viability of the bacteria decreases rapidly if the vial is rehydrated in an oxygenic environment. Incubate cultures at 37 °C and most freeze-dried cultures will grow out in a few days.

Thawing frozen MRSA: For frozen cultures, thaw MRSA using gentle agitation in a water bath that is set to 25-30°C. Thawing will be rapid; ~2 mins or until all ice crystals have melted. For anaerobic cultures, thaw the vial only in an anaerobic environment; the viability of the cell decrease rapidly if the vial is thawed in an oxygenic environment. Once the vial is thawed, decontaminate the outer surface using 70% ethanol (Sigma Aldrich Co., USA). The entire contents of the vial can then be aseptically transferred to a test tube containing 5-6 mL of BHI broth. Incubate the culture at 37 °C. Additional test tubes can be inoculated by transferring 0.5 mL of the culture of the primary culture to additional secondary cultures.

Growth of MRSA on BHI agar plates: Using a sterile applicator streak out a small amount of MRSA from the frozen stock onto a small section of a BHI agar plate using aseptic technique. With a new sterile applicator, streak out a quadrant MRSA by passing through the initial quadrant several times. Repeat this process 1-2 more times, passing a new applicator through most recently streaked quadrant. Incubate the plates for 16-24 h at 37 °C.

Growth of MRSA in BHI broth: MRSA grows rapidly in broth culture at 37 °C with aeration. As such,

overnight cultures of MRSA are typically started at the end of the day just prior to leaving the LAB using either an isolated colony from an agar plate or a frozen stock. Using septic technique and transfer a single colony of MRSA from the streak plate into the aliquoted broth by tilting the culture tube and rubbing the inoculating loop against the side of the tube at the liquid-air interface. Grow cultures overnight (~16-18 h) at 37 °C with shaking.

Preparation of MRSA frozen stocks: Long-term storage of MRSA should occur at -80 °C to prevent accumulation of mutations. To prepare a frozen stock of MRSA add an aliquot (850 μL) of an overnight culture to sterile dimethyl sulfoxide (DMSO, 150 μL, Sigma Aldrich Co., St Louis, MO, USA) to one sterile cryotube using aseptic technique and vortex/ or invert briefly to mix. The addition of DMSO prevents complete freezing of the bacteria and thus limits bacterial damage as result of the transition to -80 °C.

Antibody (Ab) conjugation **[2,3]**

The absorbance of a quantity of antibody [anti-lipopolysaccharide (LPS) antibody (Ab_{LPS}) or protein A antibody ($Ab_{protein\ A}$) (abcam, Cambridge, UK)] was recorded via UV-vis spectroscopy (Abs: approximately from 200 nm to 240 nm). By the electrostatic interaction, the materials were mixed with the same quantity antibody for 30 min of incubation at 4 °C in the dark and centrifuged (83000 rpm, >10 min) to remove excess antibody; the material–Ab_{LPS} or –$Ab_{protein\ A}$ was then prepared. On the other hand, the supernatant was retained and its absorbance measured. The difference in absorbance between the collected supernatant and the original antibody was estimated. Consequentially, the quantity of the antibody absorbed on the nanomaterials was calculated by Lambert-Beer's law. In the working solution of 1×PBS buffer, approximately 9.9 μg of Ab_{LPS} conjugated on 100 μg of amino-N-GQD 9.1. This implies that the conjugation efficiency of 1×PBS buffer was approximately 9.9% (zeta potential: 5.4 mV), and it was 10.6% (zeta potential: 6.9 mV), 12.3% (zeta potential: 7.7 mV), and 13.8% (zeta potential: 8.5 mV) for amino-N-GQD 9.9–Ab_{LPS}, amino-N-GQD 11.1–Ab_{LPS}, and amino-N-GQD 12.0–Ab_{LPS}, respectively. The above results had also proven the successful conjugation of Ab on the surface of materials. In the *E. coli* LB broth, the conjugation efficiency was approximately 10.3% (zeta potential: 6.2 mV), 11.0% (zeta potential: 7.9 mV), 12.1% (zeta potential: 9.0 mV), and 14.3% (zeta potential: 9.6 mV) for amino-N-GQD 9.1–Ab_{LPS}, amino-N-GQD 9.9–Ab_{LPS}, amino-N-GQD 11.1–Ab_{LPS}, and amino-N-GQD 12.0–Ab_{LPS}, respectively. On the other hand, the absorption efficiency was approximately 9.0% (zeta potential: 5.9 mV), 10.1% (zeta potential: 7.2 mV), 11.5% (zeta potential: 8.0 mV) and 13.0% (zeta potential: 8.3 mV) in 1×PBS buffer solution and 11.0% (zeta potential: 7.1 mV), 11.9% (zeta potential: 8.4 mV), 13.1% (zeta potential: 9.6 mV) and 15.2% (zeta potential: 10.5 mV) in MRSA BHI broth for amino-N-GQD 9.1–Ab_{LPS}, amino-N-GQD 9.9–Ab_{LPS}, amino-N-GQD 11.1–Ab_{LPS}, and amino-N-GQD 12.0–Ab_{LPS}, respectively. As there is not much difference between the zeta potential of material–Ab in the 1×PBS buffer solution, *E. coli* LB broth and MRSA BHI broth, it meant that the biomolecules would be absorbed on neither material and Ab nor material–Ab. In other words, the interaction between Ab, material–Ab and bacteria would not be influenced by biomolecules in the *E. coli* or MRSA culture media, thus leading to no subsequently effect in the specific binding between them. Moreover, the positively charged material–Ab was favorable for absorbance or

internalization by the negatively charged bacterial surface (**Scheme S2b**). The aforementioned results had also proven the successful conjugation of Ab on the surface of materials (**Table S13**).

TEM observation of negatively stained bacteria

Bacteria were picked up from colonies and suspended in a 1% aqueous sodium phosphotungstate solution (Sigma Aldrich Co., USA) at pH 7.0. Droplets of the suspensions were allowed to dry on grids coated with the Formvar. Thereafter, the samples were then subjected to TEM observation.

Viability assay of bacteria with colony forming unit (CFU) counting method

E. coli or MRSA (OD_{600} ~0.05) was mixed with material–Ab_{LPS} or –$Ab_{protein\ A}$ (0-1.5 μg mL^{-1}), at 37 °C in the dark, respectively. After incubation, the mixture was centrifuged (900 rpm for 20 min). In this step, the material was absorbed by bacteria or the material-treated bacteria can be was centrifuged and pulled down as pellets. Before plating, dilution should be carried out in 10 folds steps using the maximum recovery dilution medium (*Note: In preparing the dilutions 1 mL is transferred from the* 10^0 *suspension into 9 mL of dilute to give a dilution of* 10^{-1}. *This procedure is repeated for further dilutions using a fresh sterile pipette for each decimal dilution. The purpose of the dilutions is to ensure that the colony count per plate is in the range of 30-300 CFU for most accurate counting.*). After plating on the agar plates, the plates were placed overnight in an incubator at 37 °C in darkness. According to our definition in this study, each panel was labeled as 0.75 μg mL^{-1} and 1.5 μg mL^{-1} of concentration, respectively. The number of surviving bacteria was determined and expressed as a percentage (%) that corresponds to the unit of CFU mL^{-1} after incubation. Data are means±SD (n=6).

Singlet oxygen quantum yield (Φ_Δ) measurement **[4]**

From previous studies, Φ_Δ can be obtained. Φ_Δ measurements were conducted in D_2O at 355 nm by using *meso*-tetra(4-sulfonatophenyl)porphine dihydrochloride (Sigma Aldrich Co., St Louis, MO, USA) as a reference (Φ_Δ= 0.64).

Molecular weight of the sorted-amino-N-GQDs

The theoretical diameter of benzene is 0.243 nm with a molecular weight of 72 (ignoring the H atoms). According to **Figure S2**, the mean lateral sizes of the sorted-amino-N-GQDs were approximately 9.1±0.2 nm (amino-N-GQD 9.1), 9.9±0.2 nm (amino-N-GQD 9.9), 11.1±0.3 nm (amino-N-GQD 11.1), and 12.0±0.3 nm (amino-N-GQD 12.0), respectively. For the sorted-amino-N-GQDs, assuming there was no leakage from a layer of material and ignoring the exposed functional groups, and then the benzene number and molecular weight could be approximately 1027 and 26,217 g mol^{-1}, 1261 and 32,027 g mol^{-1}, 1519 and 38,418 g mol^{-1}, 1801 and 45,390 g mol^{-1}, respectively (**Table S14**). The following measurement for the cross section of TPE was performed using the estimated molecular weights.

***Femtosecond laser optical system for the measurements of two-photon absorption (TPA) and two-photon luminescence (TPL)* [5,6]**

The self-made femtosecond Ti-sapphire laser [a repetition rate of 80 MHz; Mai Tai with the optical parametric oscillators (OPO), Spectra-Physics, Santa Clara, CA, USA] optical system: an inverted optical microscope (Zeiss, Oberkochen, Germany), an *x–y* galvanometer scanner (Cambridge, MA, USA); a triple-axis sample-positioning stage (Prior Scientific Instruments Ltd., London, UK); a *z*-axis piezoelectric nano-positioning stage (Mad City Labs, Madison, WI, USA); photomultiplier tubes (Hamamatsu, Shinzuoka, Japan); and a data acquisition card with a field-programmable gate array module (National Instruments, Austin, TX, USA) (**Scheme S1**).

TPA measurement. A femtosecond laser was used to excite TPA signals. A time-average luminescence photon count (F) is proportional to the cross section (δ) of TPA and can be given as

$$F \approx \frac{1}{2}\delta\eta_2\phi C\frac{g_p}{f\tau}\frac{8nP^2}{\pi\lambda},$$

(Equation S1)

where C the concentration of the photoinitiator (concentration: the aqueous material was put in oven overnight to vaporize the whole liquid and weighed it. Sequentially, the dry materials can be dissolved in any applicable amount of working solution), η_2 is the quantum efficiency of photoluminescence, ϕ the luminescence collection efficiency of the system, P the average incident power, g_p the dimensionless quantity for degree of the second-order temporal coherence, f the pulse repetition rate, n the refractive index of the measurement medium, λ the excitation wavelength and τ the excitation pulse width by full-width at half maximum. After the SF-10 prism pair compensation, the parameters which are the collection system, the pulse repetition rate, the concentration of the materials and the excitation power can be maintained the same at different excitation wavelengths with their corresponding excitation pulse widths. On the basis of the measured excitation pulse width, the measured fluorescence photon count and the excitation wavelength, the TPA was derived as $\delta \times \eta_2$. *Via* the SPC module, the photomultiplier tubes were used to collect the PL photon The TPA can be given as

$$\delta\eta_2 \propto \lambda\tau F.$$

(Equation S2)

With 2 m ms^{-1} speed of the galvanometer scanner, the excitation spectrum was measured as 900–1000 nm with an excitation power of 2.0 mW [this is the power before objective; the power after objective (or on sample) is approximately 1.273 mW or 127.3 nJ $pixel^{-1}$)]. Therefore, the relative TPA spectrum as function of excitation wavelength for the material was measured.

Measurement of TPL spectrum. The material was exposed to two-photon excitation (TPE) from the femtosecond laser at an excitation wavelength of 960 nm, a power of 2.227 mW (222.7 nJ $pixel^{-1}$), a

scanning area of 200 μm × 200 μm, a frequency of 10 kHz, an exposure time of 1.638 s/(scan, pixel)= 100 μs, 128 × 128 pixels $scan^{-1}$, and a pixel area of 1,562.5 nm × 1,562.5 nm. The focal spot area was calculated as $\pi d^2/4$, where d = 0.61 λ/numerical aperture (*NA*) is the full width at half maximum of the beam waist. For instance, at the *x*–*y* axis focal spot with 960 nm excitation and a 40× oil-immersion objective with an *NA* of 1.3, *d*= 0.61 × 960 nm/1.3= 450.46 nm= 0.45046 μm (approximately 0.45 μm), and the *z*–axis resolution (full width at half maximum, FWHM) was measured to be approximately 0.90 μm (Figure 1b). For 960 nm excitation, the exposure time per scan for an individual material is expressed as (focal spot area/pixel area) × 100 = 6.53 ms, and the total exposure time *t*= 6.53 ms × number of scans. A 40× oil-immersion objective (*NA* 1.3) was used to collect the signals, and the detection range of the spectrum photometer was 500–900 nm in wavelength.

The calculations of laser power (mW or nJ $pixel^{-1}$) used on the sample were as follows. For the 40× oil-immersion objective (*NA* 1.3), the transmission rate at 960 nm in wavelength is approximately 74% in this optical system, and the laser power went from the output to the objective with 86% of the original output power due to the loss of power. As a result, the calculated energy after the objective (on sample) is P_{output} (mW)*86%*74%= 0.6364*P_{output} (mW). For instance, P_{output}= 3.5 mW, the calculated energy after the objective (on the sample) is 3.5 mW*86%*74%= approximately 2.227 mW. With 10 kHz of scan rate (each pulse stays 0.1 ms $pixel^{-1}$), the calculated energy on the sample (J $pixel^{-1}$) was around P_{output} (mW)* 86%*74%*0.1 ms= 0.06364*P_{output} (J $pixel^{-1}$). For instance, P_{output}= 3.5 mW, the energy (J $pixel^{-1}$) on sample= 3.5 mW*86%*74%*0.1 ms= 0.2227 μJ $pixel^{-1}$= 222.7 nJ $pixel^{-1}$. The power after the objective (on the sample) was used and marked throughput this manuscript.

Measurement of TPE absolute cross section **[5,6]**

The absolute cross section of TPE was measured on the basis of luminescence signals by using the self-made femtosecond Ti-sapphire laser optical system, as described above. A 40× oil-immersion objective (*NA* 1.3) was overfilled by expansion of the laser beams. For the multiphoton excitation, the diffraction-limited illumination of the sample was approximately achieved and analyzed. Under TPE and for the thick sample limit, the relation between time-averaged luminescence photon flux $\langle F^{(n)}(t)\rangle$ and the incident power *P(t)* can be obtained. The formula can be given as

$$\langle F^{(n)}(t)\rangle = \frac{1}{n}\frac{g_p^{(n)}}{(f\tau)^{n-1}}\phi\eta\sigma_n C n_0 \frac{a_n(\mathrm{NA})^{2n-4}\langle P(t)\rangle^n}{8\pi^{3-n}\lambda^{2n-3}}$$

(Equation S3)

, where C is the concentration of the fluorophore (concentration: the aqueous material was put in oven overnight to vaporize the whole liquid and weighed it. Sequentially, the dry materials can be dissolved in any applicable amount of working solution), n is the number of photons absorbed (n = 2 for the TPE), ϕ is the system collection efficiency, τ is the laser pulse width, f is the laser repetition rate, η is the luminescence quantum efficiency (or quantum yield, QY), and λ is the excitation wavelength in vacuum, σ_n is the n-photon

excitation cross section, and $a_2 = 64$ for TPE. $g_p^{(n)}$ is the nth-order temporal coherence of the excitation source. Due to the limitation of resource we currently have, $\langle F^{(n)}(t) \rangle$ is temporarily not able to be calculated and the values could be replaced by the integrated TPL intensity with the symbol "counts". As a result, the equation for action cross section ($\eta\sigma_2$) is turned into

$$\eta\sigma_2 = \frac{\text{counts}}{\frac{1}{2}\frac{g_p^{(2)}}{f_\tau} n_0 \phi C \frac{8\langle P(t)\rangle^2}{\pi\lambda}}$$

(Equation S4)

If it was with the same 2nd-order temporal coherence of the excitation source, the laser pulse width, laser repetition rate, incident power, system collection efficiency, wavelength and working concentration, the action cross section of TPE ($\eta\sigma_2$) for a fluorophore as the reference compound is determined relative to the known action cross section, then the Equation S4 is simplified as

$$(\eta\sigma_2)_2 = \frac{\text{counts}_2}{\text{counts}_1}(\eta\sigma_2)_1$$

(Equation S5)

, where Sample 1 means the reference compound, and sample 2 means the fluorophore.

The absolute cross section of TPE was measured the luminescence signal *via* femtosecond laser optical system according to previous studies. The TPL of Fluorescein and Rhodamine B (Sigma Aldrich Co., St Louis, MO, USA) had to be verified. The results are shown in **Figure 2b** and were obtained by measuring the dependence of the emission intensity with an excitation power range of 1,272.8 nJ pixel^{-1} (12.728 mW) to 5,091.2 nJ pixel^{-1} (50.912 mW). Quadratic dependence with the exponents of approximately 2.00 for Fluorescein and approximately 1.98 for Rhodamine B was measured for increasing the excitation power to determine the luminescence from TPE. According to the previous studies, the action cross sections of TPE for Fluorescein and Rhodamine B are 15.5 and 13.0 Göeppert–Mayer units (GM; 1 GM = 10^{-50} cm^{4}s photon^{-1}), respectively, for 960 nm excitation. We also referred to the free website http://www.drbio.cornell.edu/cross_sections.html, kindly provided by Prof. Chris Xu (Cornell University, NY, USA). The TPE action cross sections for Fluorescein and Rhodamine B were calculated to be 15.2 and 13.4 GM (**Table S15**), respectively, which indicated an error of less than 5% compared with those from Prof. Xu's laboratory. In this study, Rhodamine B was chosen as the standard reference for determining the cross section, and the calculated absolute cross sections of TPE for the amino-N-GQD 9.1, amino-N-GQD 9.9, amino-N-GQD 11.1, and amino-NGQD 12.0 were approximately 55,946 GM, 57,332 GM, 59,051 GM, and 60,728 GM, respectively. The measured parameters for calculating the TPE absolute cross sections of samples are shown in **Tables 1 and 2**. Additionally, the calculated absolute cross sections of TPE for the sorted dots were also calculated by using Fluorescein as the standard reference. Results show that the values of cross section (amino-N-GQD 9.1: ~56,510 GM; amino-N-GQD 9.9: ~57,993 GM; amino-N-GQD 11.1: ~59,841 GM; amino-N-GQD 12.0: ~61,449 GM) was less than only 1.4% of difference between chosen

Rhodamine B and Fluorescein (**Table S16**). No batch-to-batch variation was observed for the material in two-photon properties, two-photon photodynamic ability, and two-photon contrast agents.

Femtosecond laser optical system (for fluorescence lifetime imaging microscopy, FLIM) **[5,6]**

The self-made femtosecond Ti-sapphire laser optical system mentioned above was used. For FLIM, the time-correlated single photon counting (TCSPC) module (PicoQuant, Berlin, Germany) is integrated into the main control platform based on LabVIEW programming, which triggers the synchronic signal through the FPGA module, collects the fluorescence time-to-digital data via a USB interface, and then constructs the fluorescence lifetime image by using LabVIEW. The time-to-digital data from different pixels are separated by inserting a marker signal from scanning synchronic trigger. To facilitate a three-dimensional lifetime image and data analysis, the LabVIEW program also records scanning parameters corresponding to the time-to-digital data. The timer overflow signal of the TCSPC is removed, allowing the accumulated time-to-digital data of each pixel to form a histogram. A nonlinear least square algorithm is used to fit the fluorescence lifetime decay curve for each pixel. In accordance with the fitting lifetime data of each pixel and the pixel scanning information, the FLIM image can be displayed with a resolution of 0.1 ns under the main control platform. The lifetime data and the parameter generated using the triple-exponential equation fitting [3 exp fitting model: (a0 * exp(a1x) + a2 * exp(a3x) + a4 * exp(a5x) + a6)] while monitoring the emission under TPE. According to the iterative fitting algorithm to the fluorescence decay curve, we can estimate the 3-exponential model would have the best fitting results and it indicates the relaxation involves 3 different fluorescence emission pathways. However, in order to reduce the analysis complexity, we take all of the fluorescence emission phenomena as the average fluorescence lifetime (**Tables 2-3**).

$$t_{average} = \frac{1}{a_0 + a_1 + a_4}(a_0 \cdot t_1 + a_2 \cdot t_2 + a_3 \cdot t_3)$$

(Equation S6)

$t_{average}$: average lifetime, t_1: lifetime1, t_2: lifetime2, t_3: lifetime3

Calculation of radiative and non-radiative decay rates **[5,7]**

Photoluminescence (PL) QY and lifetime are both major parameters when investigating the emission characteristics of fluorescent dyes in diverse environments. The quantum yield Q can be expressed as follows:

$$Q = \frac{\Gamma}{\Gamma + k}$$

(Equation S7)

, where Γ is the radiative decay rate and *k* is the non-radiative decay rate.

Fluorescence lifetime is usually defined as the average time required for an electron in the excited state to decay to the ground state. The TPL lifetime τ can also be relative to the decay rates and is described as follows:

$$\tau = \frac{1}{\Gamma + k}$$

(Equation S8)

Following Equations (S7–S8), the radiative and non-radiative decay rates can be calculated.

Determination for bacteria viability rates after laser exposure **[6]**

CFU counting method: *E. coli* or MRSA (OD_{600} ~0.05) was mixed with material (0.75 μg mL^{-1}) at 37 °C in darkness. After incubation, the mixture was centrifuged (900 rpm for 20 min) and exposed to TPE [exposed to a TPE power of 222.7 nJ $pixel^{-1}$ with 20, 100 and 170 scans (approximately 0.13–1.11 s of total effective exposure time), Ex: 960 nm]. The pellets were with 3h of additional incubation time to process the PDT action. Before plating, dilution should be carried out in 10 folds steps using the maximum recovery dilution medium. After plating on the agar plates, the plates were placed overnight in an incubator at 37 °C in darkness. The number of surviving bacteria was determined and expressed as a percentage (%) that corresponds to the unit of CFU mL^{-1} after incubation. Data are means±SD (n=6).

LIVE/DEAD kit: *E. coli* (OD_{600} ~0.05) was mixed with material (0.75 μg mL^{-1}) at 37 °C in darkness. After incubation, the mixture was centrifuged (900 rpm for 20 min) and exposed to TPE [exposed to a TPE power of 222.7 nJ $pixel^{-1}$ with 20, 100 and 170 scans (approximately 0.13–1.11 s of total effective exposure time), Ex: 960 nm]. The pellets were with/without 3h of additional incubation time to process the PDT action. Then, the pellets were using a LIVE (SYTO 9, as displayed with green fluorescence) / DEAD (*propidium iodide,* PI, as displayed with red fluorescence) kit (Thermo Fisher Scientific, Waltham, MA, USA) according to the instruction. The fluorescence images were obtained using the IXplore Standard microscopy coupled with the DP23 camera and analyzed with the cellSens Entry software (Olympus, Shinjuku City, Tokyo, Japan). The viability of bacteria was quantified for antimicrobial tests, which showed nearly all nanomaterial-treated bacteria to be dead after treatment. Data are means±SD (n=6).

ROS detection **[5,8-13]**

Singlet oxygen ($^1O_2$): (a) Material (0.75 μg mL^{-1}) was treated with *E. coli* or MRSA (OD_{600} ~0.05). Then, the material-treated-*E. coli* or -MRSA was subjected to incubate at 37 °C in darkness. Subsequently, the mixture was mixed with Singlet Oxygen Sensor Green (SOSG) reagent (1 μM; Thermo Fisher Scientific, Waltham, MA, USA) (Ex/Em: 488/525 nm) and then exposed to TPE photoexcitation [222.7 nJ $pixel^{-1}$ with 20, 100 and 170 scans (approximately 0.13–1.11 s of total effective exposure time), Ex: 960 nm]. A spectrophotometer was employed for measurements. For ROS neutralization, the mixture was mixed with 30 ppm of antioxidant α-tocopherol/methyl linoleate (Sigma Aldrich Co., St Louis, MO, USA) in darkness and exposed to TPE photoexcitation with the same treatment. (b) Material (0.75 μg mL^{-1}) was treated with *E. coli* or MRSA (OD_{600} ~0.05). Then, the material-treated-*E. coli* or -MRSA was subjected to incubate at 37 °C in darkness. Subsequently, the mixture was mixed with 10 μM of trans-1-(2′-methoxyvinyl)pyrene (*t*-MVP, Thermo Fisher Scientific, USA)/0.10 M sodium dodecyl sulfate (SDS, Sigma Aldrich Co., St Louis, MO, USA) (Ex/Em: 352/465 nm) and then exposed to TPE photoexcitation [222.7 nJ $pixel^{-1}$ with 20, 100 and 170 scans (approximately 0.13–1.11 s of total effective exposure time), Ex: 960 nm]. For ROS

neutralization, the mixture was mixed with 30 ppm of antioxidant α-tocopherol/methyl linoleate (Sigma Aldrich Co., St Louis, MO, USA) in darkness. Reaction of *t*-MVP with $^1O_2$ yields a dioxetane intermediate that fluoresces while it decomposes into 1-pyrenecarboxaldehyde. Furthermore, this highly selective fluorescent probe does not react with other activated oxygen species such as hydroxyl radicals, superoxide, or hydrogen peroxide. A spectrophotometer was employed for measurements. ROS neutralization was conducted with the same as previously described treatment.

Superoxide radical anion ($O_2^{\cdot-}$): (a) Material (0.75 μg mL^{-1}) was treated with *E. coli* or MRSA (OD_{600} ~0.05). Then, the material-treated-*E. coli* or -MRSA was subjected to incubate at 37 °C in darkness. Subsequently, the mixture was mixed with 2,3-bis (2-methoxy-4-nitro-5-sulfophenyl)-2H-tetrazolium-5-carboxanilide (XTT, 0.45 mM; Sigma Aldrich Co., St Louis, MO, USA) and then exposed to TPE photoexcitation [222.7 nJ pixel^{-1} with 20, 100 and 170 scans (approximately 0.13-1.11 s of total effective exposure time), Ex: 960 nm]. The purpose of XTT was that it interacted with $O_2^{\cdot-}$ and produced XTT-formazan, resulting in strong absorption (470 nm in wavelength). UV-vis spectrometer was employed to monitor this absorption. For ROS neutralization, the mixture was mixed with 30 ppm of antioxidant α-tocopherol/methyl linoleate (Sigma Aldrich Co., St Louis, MO, USA) in darkness and exposed to TPE photoexcitation with the same treatment. (b) Material (0.75 μg mL^{-1}) was treated with *E. coli* or MRSA (OD_{600} ~0.05). Then, the material-treated-*E. coli* or –MRSA was subjected to incubate at 37 °C in darkness. Subsequently, the mixture was mixed with 50 mM bicarbonate buffer (pH 8.60) and glutathione (γ-L-glutamyl-L-cysteinyl-glycine, GSH; Sigma Aldrich Co., St Louis, MO, USA)/0.80 mM bicarbonate buffer (the Ellman's assay for $O_2^{\cdot-}$ detection) and then exposed to TPE photoexcitation [222.7 nJ pixel^{-1} with 20, 100 and 170 scans (approximately 0.13-1.11 s of total effective exposure time), Ex: 960 nm]. Subsequently, the following experiments were conducted according to the procedure in a previous study. Loss of GSH (%) was calculated as the difference in absorbance between the sample and negative control divided by the absorbance of the negative control. The signal of the generated $O_2^{\cdot-}$ was obtained as described in the previous calculation. Data are means±SD (n = 6). For ROS neutralization, the mixture was mixed with 30 ppm of antioxidant α-tocopherol/methyl linoleate (Sigma Aldrich Co., St Louis, MO, USA) in darkness and exposed to TPE photoexcitation with the same treatment.

Hydroxyl peroxide (H_2O_2), hydroxyl radical ($OH^{\cdot}$) and peroxynitrite ($ONOO^-$): (a) Material (0.75 μg mL^{-1}) was treated with *E. coli* or MRSA (OD_{600} ~0.05). Then, the material-treated-*E. coli* or -MRSA was subjected to incubate at 37 °C in darkness. Subsequently, the mixture was mixed with diluted 2′,7′-dichlorodihydrofluorescein diacetate (H_2DCFDA) solution [H_2DCFDA 2.5 μg (Sigma Aldrich Co., St Louis, MO, USA)+99.5% EtOH (Sigma Aldrich Co., St Louis, MO, USA) 500 μL, and diluted 1000-fold with 1×PBS to a final volume of 500 mL] and then exposed to TPE photoexcitation [222.7 nJ pixel^{-1} with 20, 100 and 170 scans (approximately 0.13–1.11 s of total effective exposure time), Ex: 960 nm]. The colorless H_2DCFDA passes through cell membranes and converts itself into 2′,7′-dichlorodihydrofluorescin (DCFH). In the presenceof H_2O_2, OH· and $ONOO^-$, DCFH is oxidized to dichlorodihydrofluorescein (DCF), which emits green fluorescence (Ex/Em: 498/522 nm). A spectrophotometer was employed for measurements. For ROS neutralization, the mixture was mixed with 30 ppm of antioxidant

α-tocopherol/methyl linoleate (Sigma Aldrich Co., St Louis, MO, USA) in darkness and exposed to TPE photoexcitation with the same treatment.

Two-photon imaging (TPI)

Type I collagen (BD, Franklin Lakes, NJ, USA) with original concentration of 4 mg mL^{-1} was diluted with 10X PBS and water to achieve final concentration of 1X PBS and 2 mg mL^{-1} collagen. NaOH was added to neutralize collagen solution before mixing with *E coli*. Bacteria, materials–Ab or materials–Ab-bacteria in LB were mixed with collagen solution, and the mixture was added to chambered coverglass (Thermo Fisher Scientific, Waltham, MA, USA). Collagen was polymerized at 20°C for 1 h, and then at 37°C for 20 mins. Full medium was applied after polymerization. Medium was daily changed after collagen polymerization for both collagen-only and bacteria-collagen matrix to ensure minimum variation caused by evaporation. Matrices were kept at 37°C, 5% CO_2 incubator. Images were taken at 20°C after collagen polymerization.

Statistical Analysis

Statistical analysis was conducted by SPSS. *p* values were obtained using the 2-sample *t* test, with $p^* < 0.05$ considered to be statistically significant difference and $^{**}p < 0.01$, $^{***}p < 0.001$, $^{****}p < 0.0001$ considered to be markedly significant difference, respectively.

And there is no batch to batch variation for the sorted materials in terms of two-photo properties, and two-photon contrast agents. Different optical system has different detection depth. Due to the detection efficiency and the objective we used, the maximal *z* depth we can be observed by this laser optical system is around 180 μm. However, less than 180 μm in the work can show the optimal resolution in the mimic biological specimens.

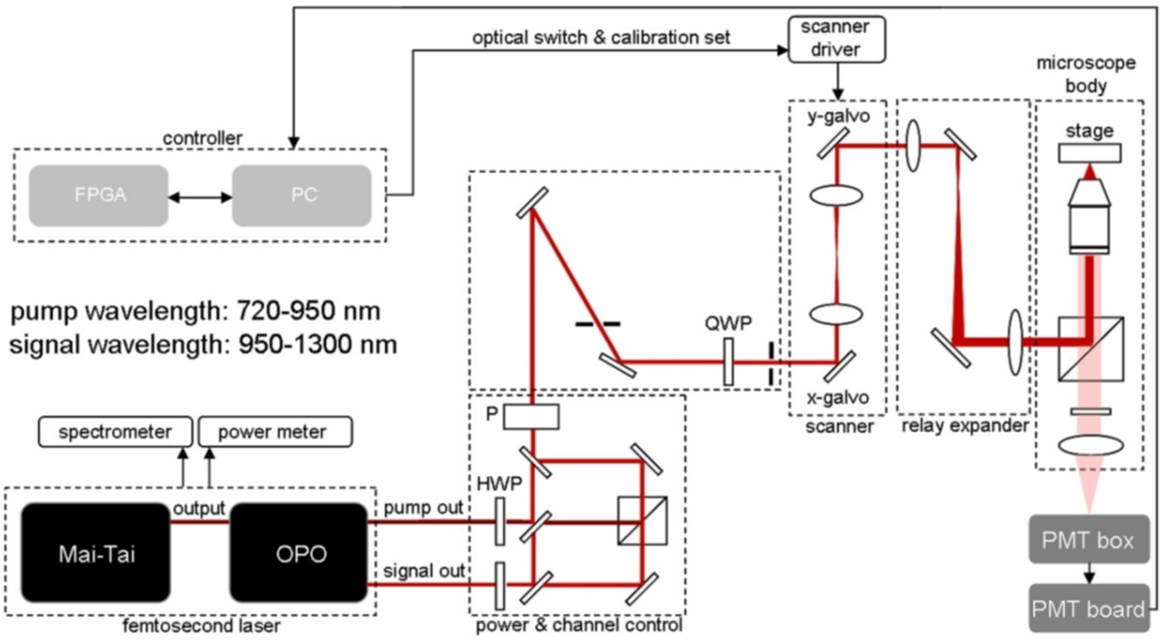

Scheme S1. The optical setup and the femtosecond laser optical imaging system.

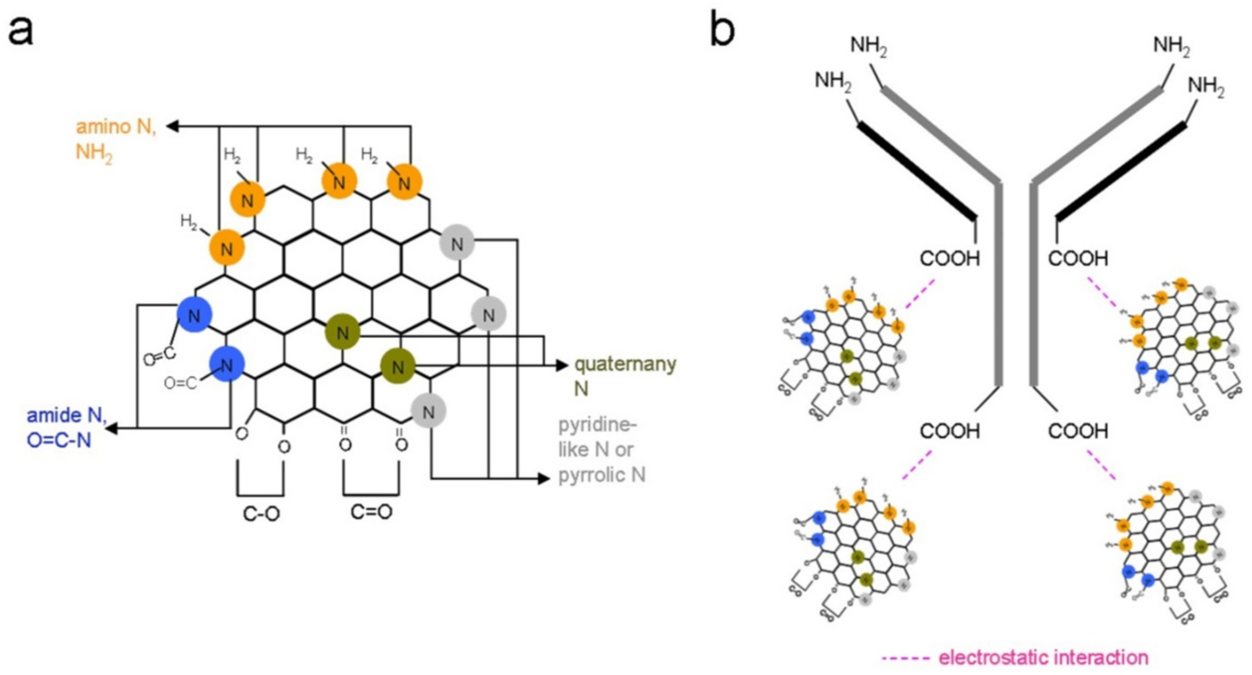

Scheme S2. The conceptual schemes of (a) the amino-N-GQD, and (b) amino-N-GQD-Ab.

Amino-N-GQDs, the estimated mean lateral size of the derived N-GQDs was prepared by an ultrasonic shearing reaction from graphene oxide sheets. The mean lateral size of the amino-N-GQD was approximately 10.1±0.7 nm (**Table S1**), as determined using low-magnification (**Figure S1a**) and high-resolution TEM (HR-TEM) images (inset images of **Figure S1a**), this indicated that the interlayer spacing of the as-prepared amino-N-GQDs was 0.213 nm; we determined this spacing to correspond to the *d*-spacing of the $\{1\bar{1}00\}$ lattice planes of graphene. The height profiles of the materials were determined through atomic force microscopy (AFM), and the results indicated a single 1.25±0.09-nm-thick layer of amino-N-GQDs (**Figure S1b**) [14,15]. For the UV-vis absorption spectrum of the amino-N-GQDs, the peaks can be observed. The amino-N-GQDs showed peaks about 226 nm ($\pi-\pi^*$ transition of aromatic C=C bonds), and 310 nm ($n-\pi^*$ transition of C=O shoulder and C−N) (**Figure S1c**). FTIR was used to analyze the exposed functional groups of the amino-N-GQDs (**Figure S1d**). The results showed characteristic bands of amino-N-GQDs for N−H vibration about 904 cm^{-1} (band 1), C−O stretching about 1082 cm^{-1} (band 2), C−N stretching about 1136 cm^{-1} (band 3), N−C=O stretching about 1205 cm^{-1} (band 4), tertiary alcoholic C−OH bending about 1284 cm^{-1} (band 5), C=C ring about 1632 cm^{-1} (band 6), N−H bending about 1695 cm^{-1} (band 7), amide about 1689 cm^{-1} (band 8), C=O stretching about 1831 cm^{-1} (band 9), N−H stretching about 2388 cm^{-1} (band 10), C−H stretching about 2850 cm^{-1} (band 11) and N−H vibration about 3336 cm^{-1} (band 12). The results indicated the functional groups of epoxy, hydroxyl, carbonyl, and nitrogen functionalities groups were exposed from amino-N-GQDs. Raman spectroscopy was also used to examine the crystallinity of amino-N-GQDs (**Figure S1e**). The major feature bands of amino-N-GQDs are the so-called G bands (~1607 cm^{-1}), which result from the in-plane vibration in a two-dimensional hexagonal lattice of sp^2 hybridized C-C bonds; the D band resulted from the defect, disorder, and sp^3-hybridized carbon in graphene layers by breaking the translational symmetry of the lattice occurring at about 1383 cm^{-1}. The results showed that the integrated intensity ratio of the D and G bands (I_D/I_G ratio), which represents the extent of disorder, was 0.91, which is much higher than that of graphite (0.105), clearly indicating higher distortion of the amino-N-GQDs. Besides, the initial graphene film has a I_D/I_G of 1.1, which was reduced to 0.91 after the oxidation reaction. This result indirectly indicates that the defect lies within the graphene film having been preferentially attacked for the oxidation to break the starting reduced graphene oxide film into tiny pieces of fewer defects, hence the observed reduced I_D/I_G, indicating the successful conversion from graphite, graphene oxide to amino-N-GQDs. XRD, which was performed to analyze crystallinity, showed the diffraction angle of the small size of amino-N-GQDs peaked broadly at about 24.3°, which means an interlayer distance of 0.360 nm (**Figure S1f**). The XRD results suggested that there was no significant basal plane functionalization, which is consistent with our understanding that the graphenes have the much more active edges than the in-plane carbons, as well as the existence of functional groups at the edges of the amino-N-GQDs. The value for one amino-N-GQD is in agreement with values, 0.340–0.403 nm [5], reported for the amino-N-GQD prepared with other methods and the as-prepared amino-N-GQDs were confirmed to have a lamellar, well-ordered structure. The accommodation of various oxygen species, such as epoxy, hydroxyl, carbonyl groups, and changes in the carbon hexahedron grid plane that increase the spacing of the graphene layers result in the increased basal spacing of the amino-N-GQDs. The surface chemistry of amino-N-GQD was determined by XPS (**Figure S1g,h**). The deconvoluted carbon C(1s) spectra of amino-N-GQD were from C−C/C=C (285.6 eV), C−N (286.3 eV), C−O (286.9 eV) and C=O (288.1 eV), respectively; the deconvoluted N(1s) spectra of amino-N-GQD was pyridinic N (398.2 eV), amino N (398.9 eV), pyrrolic N (399.9 eV), quaternary N (400.4 eV), and amide N (401.2 eV), respectively. The C(1s) and N(1s) spectrum of N-GQD showed the O(1s)/C(1s) and N(1s)/C(1s) ratios to be about 35.0% and 5.2%. The table in **Figure S1** summarizes the bonding composition and atomic ratio of the amino-N-GQDs. The characterization results indicate that preparation of the amino-N-GQDs was successful.

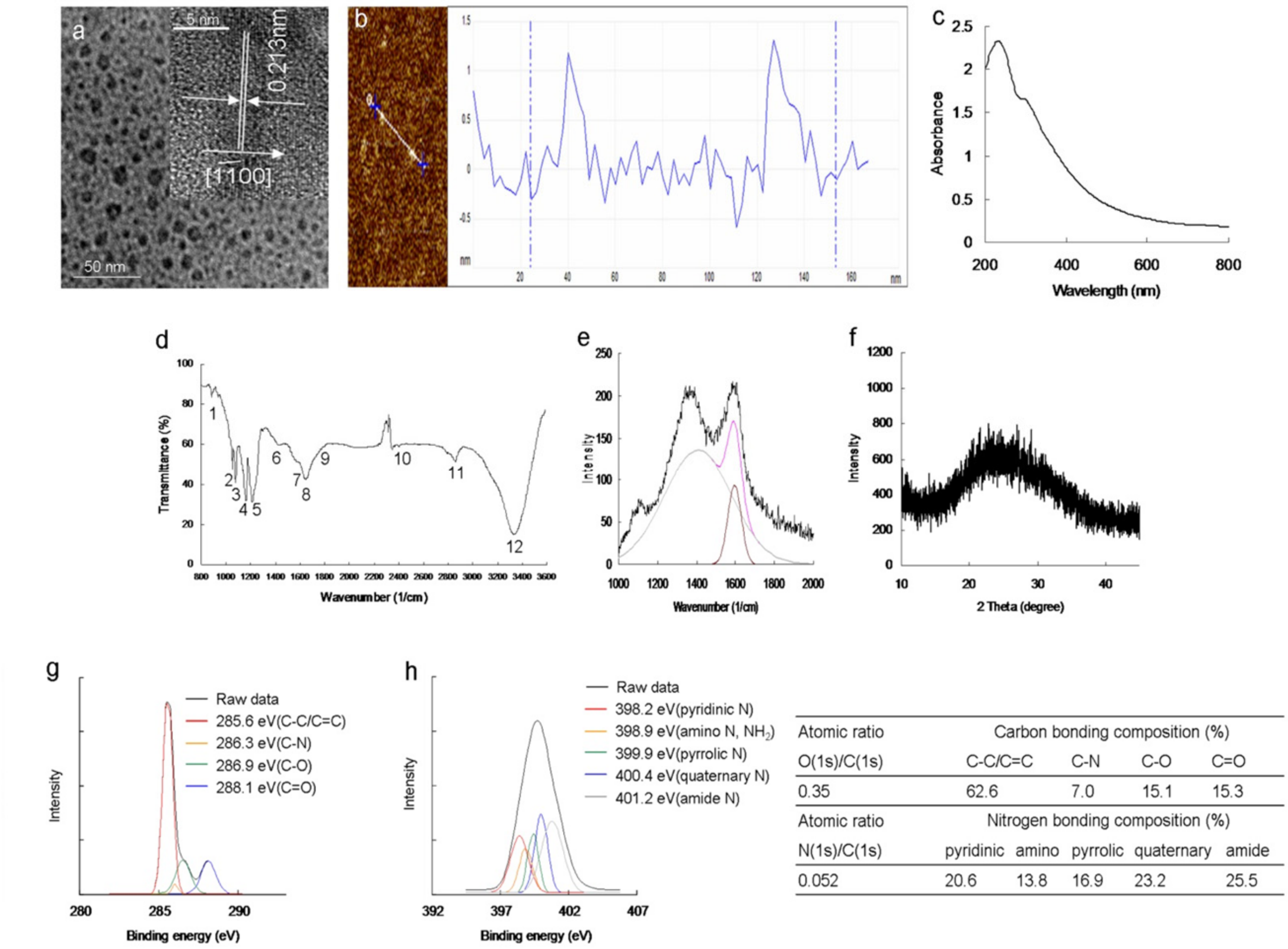

Atomic ratio	Carbon bonding composition (%)				
O(1s)/C(1s)	C-C/C=C	C-N	C-O	C=O	
0.35	62.6	7.0	15.1	15.3	
Atomic ratio	Nitrogen bonding composition (%)				
N(1s)/C(1s)	pyridinic	amino	pyrrolic	quaternary	amide
0.052	20.6	13.8	16.9	23.2	25.5

Figure S1. Characterization of material. (a) Low-magnified TEM image and (a, inset) HR-TEM image captured for a single amino-N-GQD (mean lateral size= ~10.1±0.7 nm; interlayer spacing = 0.213 nm); the size distribution of its histogram was determined using dynamic light scattering (DLS, **Table S1**). (b) AFM image of amino-N-GQDs on mica. The height difference between two arrows (the amino-N-GQDs and mica) was 1.25±0.09 nm. (c) UV-vis and (d) FTIR spectra of amino-N-GQDs. (e) Raman spectrum of amino-N-GQDs, with the gray and brown lines representing the spectrum that was decomposed and fitted to D- and G-band peaks (at ~1383 and 1607 cm^{-1}, respectively; black line: raw data; pink line: decomposed spectrum). (f) XRD pattern of amino-N-GQDs. (g,h) XPS spectrum of amino-N-GQDs, obtained to determine chemical state changes. In the deconvoluted C(1s) spectrum, the peaks fit using the Gaussian function correspond to C–C/C=C, C–N, C–O, and C=O. In the deconvoluted N(1s) spectrum, the peaks fit using the Gaussian function correspond to pyridinic N, amino N, pyrrolic N, quaternary N, and amide N. The table summarizes the bonding composition and atomic ratio of the amino-N-GQDs. The O(1s)/C(1s) and N(1s)/C(1s) atomic ratios were 35.0% and 5.2%, respectively. Delivered dose: from 0.75 μg mL^{-1} to 7.5 mg mL^{-1} material.

Table S1. Quantitative size distribution was determined by DLS. Delivered dose: 7.5 mg mL^{-1} material.

Size (nm)	6.5	7.5	8.5	9.5	10.5	11.5	12.5	13.5
Fraction (%)	0	7	14	18	30	29	2	0

Raman spectroscopy was used to monitor the inelastic phonon scattering caused by the vibration of chemical bonds which enables to calculate the size of the sp^2 domain in the amino-N-GQDs. If the mean size of the amino-N-GQDs >3 nm, follow the Equation S9 [16,17],

$$L_a \text{ (nm)} = (2.4 \times 10^{-10})\ \lambda_{laser}^4\ (I_D/I_G)^{-1}$$

(Equation S9)

where L_a (nm) is the mean size of the sp^2 domain; λ_{Laser} is the excitation wavelength (nm); I means the intensity for the D band and G band, respectively.

However, if the mean size of the amino-N-GQDs <3 nm, follow the Equation S10 [18],

$$L_D = 0.54\ E_L^4\ (I_D/I_G)$$

(Equation S10)

where is that in small-size graphene sheets with point-like defects containing sp^3-bonded carbon atoms, the mean distance between defects, L_D (nm), is generally used to represent the size of the sp^2 domains $L_{D,}$ (nm); E_{Laser} is the excitation laser energy (eV) used in Raman experiments.

The Raman spectra (**Figure S1e**) were obtained with a 532 nm laser and were decomposed into the D band and G band by the Lorentzian function. According to the calculations based on the Equations S9–S10, the diameter of the amino-N-GQDs was approximately 10.2 nm (compared to the amino-N-GQDs with a diameter of 10.1±0.7 nm in **Figure S1a**). Results showed that the calculated values were almost consistent with that shown in **Figure S1a and Table S1**.

Accordingly, size-based sorting was performed for the prepared amino-N-GQDs. Specifically, the amino-N-GQDs were sieved through polypropylene membranes of different pore sizes (e.g., 50, 100 and 300 kD). The sorted N-GQDs were then subjected to low-magnification-TEM (**Figures S2a-d**) and HR-TEM (inset images of **Figures S2a-d**), yielding mean lateral sizes of 9.1±0.2 nm (amino-N-GQD 9.1), 9.9±0.2 nm (amino-N-GQD 9.9), 11.1±0.3 nm (amino-N-GQD 11.1), 12.0±0.3 nm (amino-N-GQD 12.0) (**Figure S2**). Moreover, the calculated interlayer spacing in the materials was 0.213 nm, corresponding to the *d*-spacing of the $\{1\bar{1}00\}$ graphene lattice plane. Furthermore, XPS was performed to analyze the sorted N-GQDs' surface chemistry. The N:C and O:C atomic ratios were 0.050–0.059 and 0.30–0.39, respectively, indicating that the sorted amino-N-GQDs had similar degrees of surface oxidation (**Figures S3-S4**). As particle size increased, C–C/C=C and C–N were favorable for large materials. As amino-N-GQD size decreased, the quantities of C–O, C=O, and quaternary or amide–N functionalities increased, and this was probably because of the increased edge sites, indicating that the quantity of O– and N–based groups influenced O and N content. However, the concentration of pyrrolic-, amino- or pyrrolic-N increased with amino-N-GQD particle size probably because the oxidized interaction mainly occurred in five-membered aromatic heterocycles as amino-N-GQD size increased.

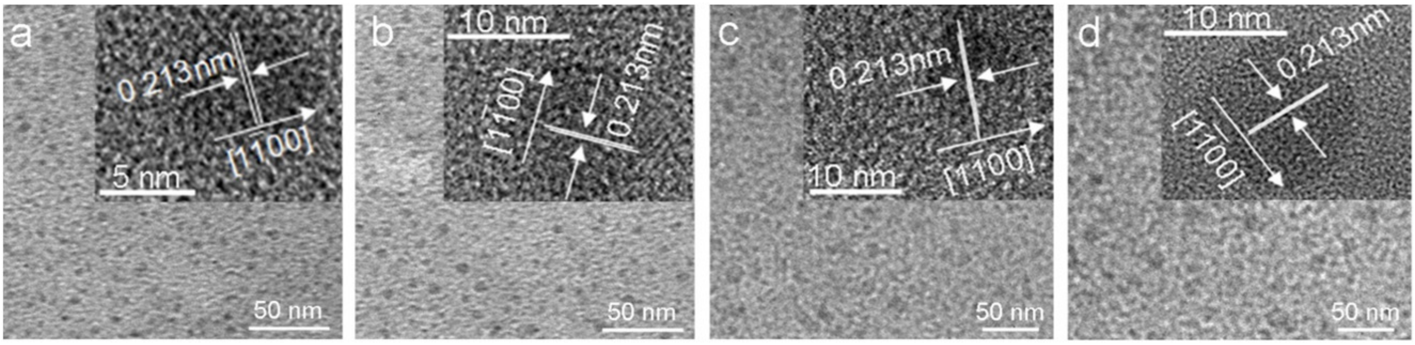

Figure S2. TEM images. Low-magnified TEM images of (a) amino-N-GQD 9.1, (b) amino-N-GQD 9.9, (c) amino-N-GQD 11.1, and (d) amino-N-GQD 12.0; HR-TEM images of (a, inset) amino-N-GQD 9.1, (b, inset) amino-N-GQD 9.9, (c, inset) amino-N-GQD 11.1, and (d, inset) amino-N-GQD 12.0 with diameters of 9.1±0.2 nm, 9.9±0.2 nm, 11.1±0.3 nm, and 12.0±0.4 nm, respectively. The images depict graphene $\{1\bar{1}00\}$ lattice planes with a 0.213-nm *d*-spacing for the sorted amino-N-GQDs. Delivered dose: 0.75 μg mL^{-1} material.

The deconvoluted C(1s) spectra of the sorted N-GQDs were from a C−C/C=C, C−N, C−O and C=O, respectively (**Figure S3**). The C(1s) spectra of the sorted amino-N-GQDs showed the O(1s)/C(1s) ratios to be about from 30% to 39% (inset table of **Figure S3**).

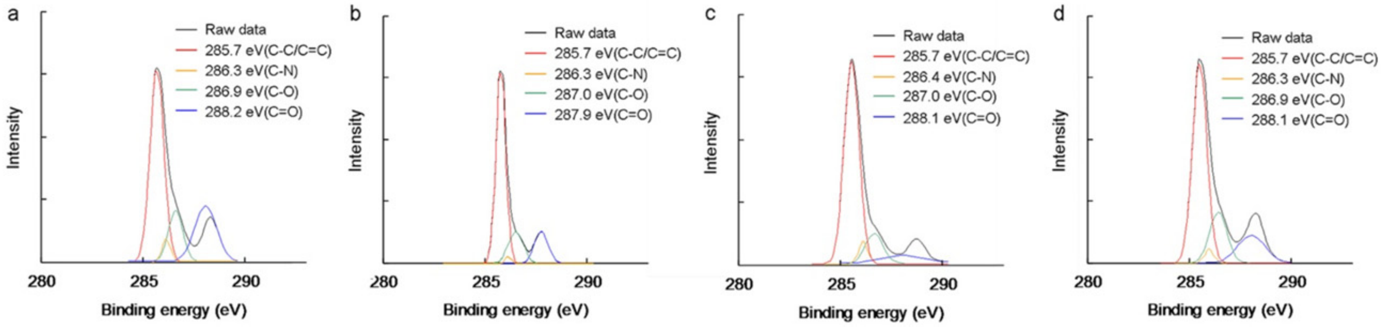

	Atomic ratio	Carbon bonding composition (%)			
	O(1s)/ C(1s)	C-C/ C=C	C-N	C-O	C=O
amino-N-GQD 9.1	0.30	60.7	6.5	14.6	18.2
amino-N-GQD 9.9	0.34	63.1	7.7	14.4	14.8
amino-N-GQD 11.1	0.36	64.0	8.2	13.6	14.2
amino-N-GQD 12.0	0.39	64.9	8.8	12.8	13.5

Figure S3. XPS. C(1s) XPS spectra of the sorted amino-N-GQD: (a) amino-N-GQD 9.1, (b) amino-N-GQD 9.9, (c) amino-N-GQD 11.1, and (d) amino-N-GQD 12.0, respectively. The deconvoluted C(1s) XPS spectra and fitted peaks by using Gaussian function. The atomic ratios and bonding compositions for amino-N-GQDs are summarized in the table. O(1s)/C(1s) atomic ratios are from 0.030 to 0.39. Delivered dose: 0.75 μg mL^{-1} material.

The deconvoluted N(1s) spectra of the sorted amino-N-GQDs were from a pyridine-N, amino-N, pyrrolic-N, quaternary-N, and amide N, respectively (**Figure S4**). The N(1s) spectra of the sorted amino-N-GQDs showed the N(1s)/C(1s) ratios to be approximately from 5.0% to 5.9% (inset table of **Figure S4**).

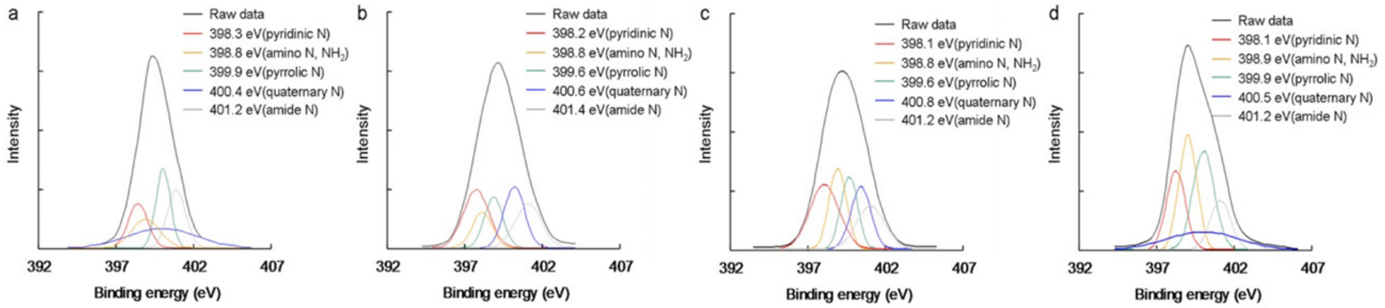

	Atomic ratio	Nitrogen bonding composition (%)				
	N(1s)/ C(1s)	Pyridine	Amino	Pyrrolic	Quaternary	Amide
amino-N-GQD 9.1	0.050	14.1	11.8	19.4	36.2	18.5
amino-N-GQD 9.9.	0.052	23.5	16.0	20.5	22.4	17.6
amino-N-GQD 11.1	0.055	25.4	20.1	21.5	17.9	15.1
amino-N-GQD 12.0	0.059	26.3	27.7	26.9	12.3	6.8

Figure S4. XPS. N(1s) XPS spectra of the sorted amino-N-GQD: (a) amino-N-GQD 9.1, (b) amino-N-GQD 9.9, (c) amino-N-GQD 11.1, and (d) amino-N-GQD 12.0, respectively. The deconvoluted N(1s) XPS spectra and fitted peaks by using Gaussian function. The atomic ratios and bonding compositions for amino-N-GQDs are summarized in the table. N(1s)/C(1s) atomic ratios are from 0.050 to 0.059. Delivered dose: 0.75 μg mL^{-1} material.

Raman spectra (**Figure S5**) were obtained with a 532 nm laser and were decomposed into the D band and G band by the Lorentzian function. According to the calculations based on the Equations S9-S10 [18-21], the diameter of the sorted amino-N-GQD was approximately 9.0 nm (amino-N-GQD 9.1), 9.9 nm (amino-N-GQD 9.9), 11.2 nm (amino-N-GQD 11.1), and 11.9 nm (amino-N-GQD 12.0), respectively. The results were consistent with those in **Figure S2**.

The position of the D band and G band for amino-N-GQD 9.1 (**Figure S5a**) was 1385 cm^{-1} and from 1606 cm^{-1}, respectively; the position of the D band and G band for amino-N-GQD 9.9 (**Figure S5b**) was 1385 cm^{-1} and 1607 cm^{-1}, respectively; the position of the D band and G band for amino-N-GQD 11.1 (**Figure S5c**) was 1386 cm^{-1} and 1606 cm^{-1}, respectively; the position of the D band and G band for amino-N-GQD 12.0 (**Figure S5d**) was 1386 cm^{-1} and 1607 cm^{-1}, respectively.

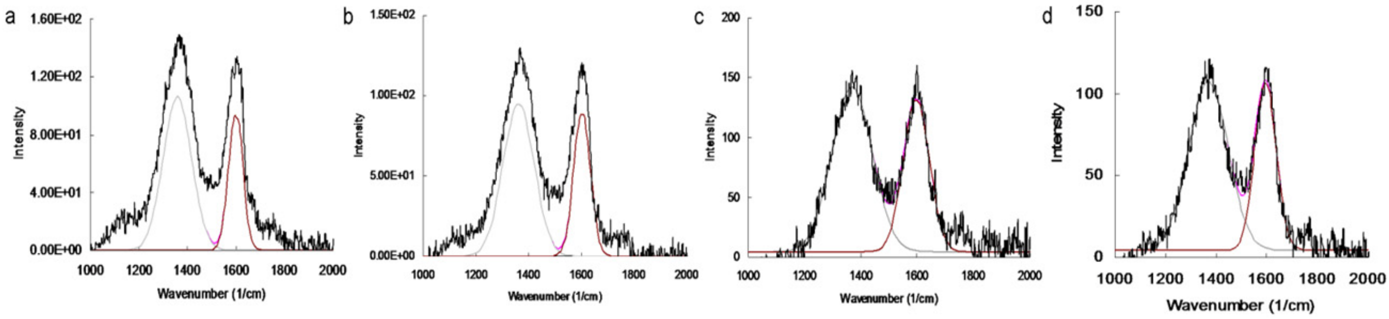

Figure S5. Raman. Raman spectra of (a) amino-N-GQD 9.1, (b) amino-N-GQD 9.9, (c) amino-N-GQD 11.1, and (d) amino-N-GQD 12.0, respectively. Excitation laser wavelength: 532nm. The deconvoluted Raman spectra and fitted peaks by using Gaussian function. The Raman spectra were decomposed into the D band and G band (black line: raw data; pink line: decomposed spectrum; gray line: D band; G band: brown line). Delivered dose: 0.75 μg mL^{-1} material.

The sorted amino-N-GQDs emitted peak were observed at approximately 719 nm (amino-N-GQD 9.1), 772 nm (amino-N-GQD 9.9), 810 nm (amino-N-GQD 11.1), and 862 nm (amino-N-GQD12.0), respectively, with the excitation wavelength encompassed from UV to NIR window. The sorted amino-N-GQDs showed the phenomenon of EWI-PL emission (**Figure S6**).

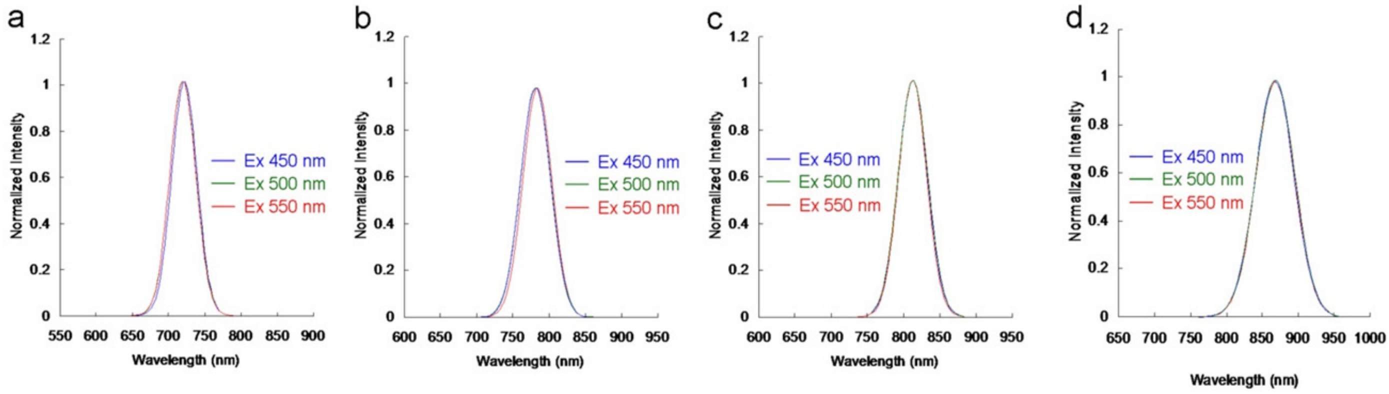

Figure S6. Spectra with EWI-PL feature of the sorted amino-N-GQDs: (a) amino-N-GQD 9.1, (b) amino-N-GQD 9.9, (c) amino-N-GQD 11.1, and (d) amino-N-GQD 12.0 respectively.

To identify the valence band edges (e.g., the *n*-state, denoted as E_v) of the sorted amino-N-GQDs, we respectively deposited them on the silicon substrate and determined the E_v by using ultraviolet photoelectron spectroscopy (UPS) with He I light (21.2 eV) irradiation. The following Equation S11 was used for UPS analysis:

$$E_B + E_k + \phi = 21.2 \text{ eV} \quad \text{(Equation S11)}$$

where E_B is the binding energy measured from the Fermi level, E_k is the kinetic energy of electrons, φ is the work function of the sorted N-GQDs, and 21.2 eV is the energy of the He I light. The Ev can be calculated using the following Equation S12:

$$E_v = 21.2 \text{ eV} - (E_{B2} - E_{B1}) \quad \text{(Equation S12)}$$

where E_{B2} is the secondary cutoff binding energy in the UPS spectra, in which the E_k of the excited electrons is equal to 0; E_{B1} represents the difference between the Fermi edges and the valence band edges. **Figure S7** shows the UPS spectra of the sorted amino-N-GQDs. The E_{B1} can be determined using the intercepts of the extrapolated straight lines on the abscissa at low binding energy. The E_{B2} can be estimated using the secondary cutoff values (E_k = 0 eV) in the UPS spectra, obtained from the intercepts of the extrapolated straight lines on the abscissa at high binding energy. The UPS widths are the difference between E_{B2} and E_{B1}. The E_v can be calculated by subtracting these UPS widths from the excitation energy (21.2 eV).

UV-vis absorption spectrum of the amino-N-GQDs, the peaks can be observed. The amino-N-GQDs showed peaks about 226 nm (*π*—*π** transition of aromatic C=C bonds), and 310 nm (*n*—*π** transition of C=O shoulder and C—N) (**Figure S1c**). The PL spectra of the sorted N-GQDs exhibited a UV- to- NIR shift, as revealed by a PL spectrometer. Emission peaks determined for amino-N-GQD 9.1, amino-N-GQD 9.9, amino-N-GQD 11.1, and amino-N-GQD 12.0 were noted at nearly 719, 772, 810, and 862 nm, respectively, and exhibited the EWI-PL feature (**Figure S11**). Furthermore, the sorted amino-N-GQDs exhibited different PL emissions in wavelengths encompassing the UV to NIR windows at 630 nm (**Figure 1a**); these emissions were short-wavelength peak attributed to the n → σ* transition. Furthermore, UV photoelectron spectroscopy was performed to determine the highest occupied orbital levels of the sorted N-GQDs. The n-state levels were located at nearly the same energetic positions (6.6-6.8 eV), regardless of size (**Figure S7**). The quantum confinement effect due to particle size regulated the wavelengths of the radiative transitions. In addition, the sorted amino-N-GQDs emitted EWI-PL, implying no trap states between the n-state and π* energy levels. Changing the particle size did not cause disturbances in the n–state level. The EWI-PL of the sorted N-GQDs could be attributed to the π* → n recombination that entailed an electron transition and phonon scattering. For short wavelength-excitation-induced PL emissions, the electrons must relax from the σ* to the π* orbital. Furthermore, the absolute QYs of amino-N-GQD 9.1, amino-N-GQD 9.9, amino-N-GQD 11.1, and amino-N-GQD 12.0 were 0.39, 0.41, 0.45, and 0.48 respectively. The characterization processes confirmed the successful preparation of sorted amino-N-GQDs with EWI-PL characteristics.

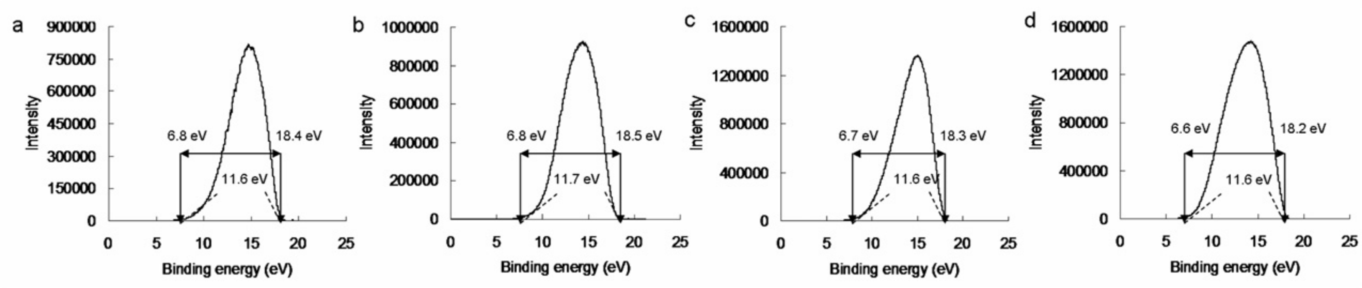

Figure S7. UPS. UPS spectra of (a) amino-N-GQD 9.1, (b) amino-N-GQD 9.9, (c) amino-N-GQD 11.1, and (d) amino-N-GQD 12.0, respectively. The valence band levels with respect to the Fermi levels were determined from the intercepts of the extrapolated straight lines (dashed line) on the abscissa at low binding energy. The intersections of the tangent (dashed line) with the abscissa at high binding energy give the secondary onset binding energy. The UPS widths can be determined from these two binding energies, and the valence band edges can be calculated by subtracting these widths from the excitation energy (21.2 eV here). Delivered dose: 0.75 μg mL^{-1} material.

Table S2. Stability of newly prepared and as-prepared sorted-amino-N-GQD over 3 months in physiological environment of *E. coli* LB broth determined by Raman (Equations S9–S10, please refer to the Materials and methods) and zeta potential spectroscopy. [Cell type for zeta potential: DTS1060C; Measurement duration: 30 number of runs, 20 run duration (sec)]. Delivered dose: 7.5 mg mL^{-1} material.

Mean lateral size (nm)/ Zeta potential (mV)	Newly prepared	As-prepare for 3 months
amino-N-GQD 9.1-Ab_{LPS}	9.0 nm/ 6.2 mV	9.1 nm/ 6.4 mV
amino-N-GQD 9.9-Ab_{LPS}	9.9 nm/ 7.9 mV	10.0 nm/ 8.0 mV
amino-N-GQD 11.1-Ab_{LPS}	11.2 nm/ 9.0 mV	11.2 nm/ 9.1 mV
Amino-N-GQD 12.0-Ab_{LPS}	11.9 nm/ 9.6 mV	12.0 nm/ 9.8 mV

This study employed the experimental template of gram-negative *E. coli*. The surface protein, namely antilipopolysaccharide (LPS), on the cell wall of *E. coli* was considered; thus, the material was coated with an anti-LPS antibody (Ab_{LPS}) to form a material–Ab_{LPS} hybrid, leading to enhance specificity, selectivity, and efficiency. A CFU assay was performed to examine material biocompatibility. The number of bacterial cells was calculated as $\log_{10}$ CFU per milliliter ($\log_{10}$ CFU mL^{-1}) and is expressed herein as a percentage (**Figure S8**). The executed CFU assay revealed the sorted amino-N-GQDs to be highly biocompatible. Additionally, the toxicity of the material was discovered to contribute to the elimination of bacteria in PDT.

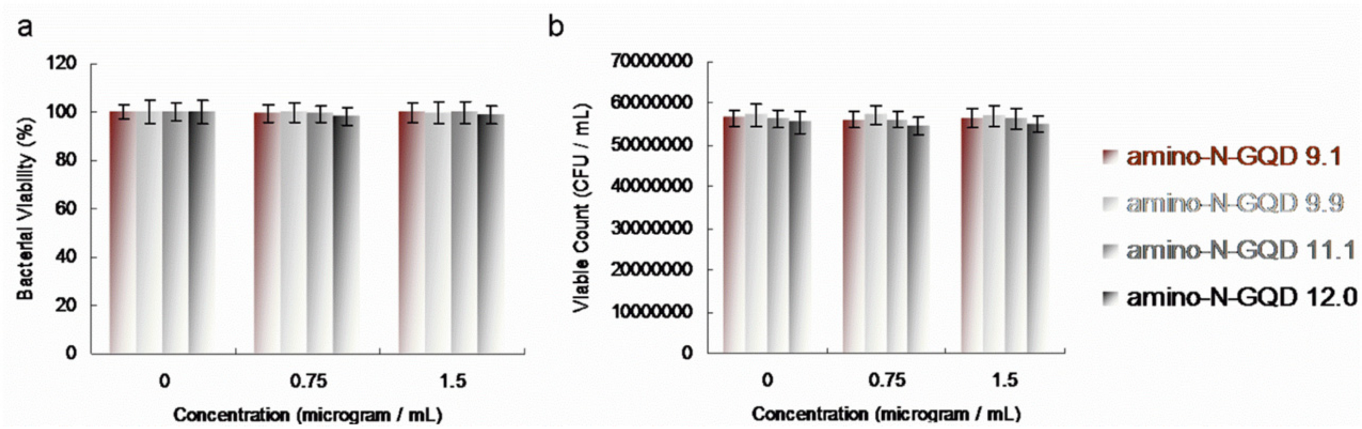

Figure S8. CFU counting assay. Number of surviving (a,b) the sorted amino-N-GQD–Ab_{LPS} -treated *E. coli* cells (unit: CFU/mL), as determined using a CFU counting assay, and *E. coli* cells (expressed as a percentage). Delivered dose: OD_{600} = ~0.05 for bacteria and 0–1.5 μg mL^{-1} for material–Ab_{LPS}. Data are presented as mean ± SD (*n*=6).

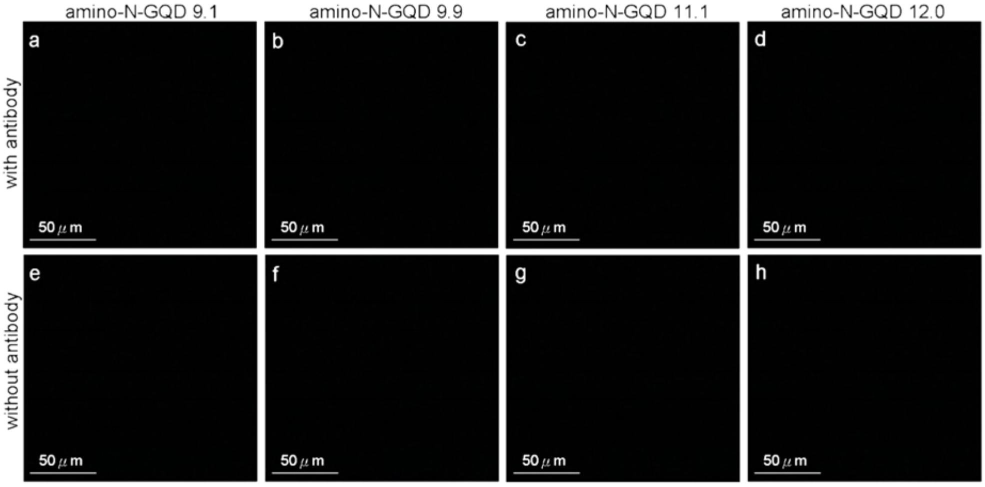

Figure S9. TPL images. TPL images (gray-level) of *E. coli* subjected to (a,e) amino-N-GQD 9.1, (b,f) amino-N-GQD 9.9, (c,g) amino-N-GQD 11.1, and (d,h) amino-N-GQD 12.0 (a–d) treatments with or (e–h) without antibody coating and incubated for 3 h, as observed at a 200-μm depth (222.7 nJ pixel^{-1} with 170 scans, approximately 1.11 s of total exposure time) under TPE (Ex: 960 nm). Delivered dose (OD_{600}): approximately 0.05 of *E. coli*; 0.75 μg mL^{-1} material or 0.75 μg mL^{-1} material–Ab_{LPS}..

The generated ROS to involve photodynamic therapy (PDT) action would lead to induce DNA damage, inactivate enzymes, and oxidize amino acids, which all cause bacterial injury. But it may have an additional incubation time to effectively process the PDT action. The surviving viabilities of bacteria were also indicated by fluorescence and quantification (**Figure S10**). Amino-N-GQD 12.0 was selected to conduct this experiment. For the photoexcited bacteria alone with 3h of additional incubation (viability >99%; **Figures S10a,d**) and the photoexcited, amino-N-GQD 12.0–Ab$_{LPS}$ treated bacteria without incubation time (viability >99%; **Figures S10b,d**), results showed nearly no damage, as evidenced by the green fluorescence, indicating live bacteria. Indicates that not only indicates that it took time to proceed the PDT but also no photothermal heating effect which was generated from materials and would immediately cause destruction of cells was determined. Further, the dead bacteria from the amino-N-GQD 12.0–Ab$_{LPS}$ treatments and laser exposure were distinguishable to a particular degree with 3h of additional incubation, as indicated by the red fluorescence (**Figures S10c,d**). The viability of bacteria was quantified for additional antimicrobial tests, which showed nearly amino-N-GQD 12.0–Ab$_{LPS}$ treated bacteria to be dead after treatment (elimination >99%; **Figures S10c,d**). Besides, bacteria treated without antibody-coated materials exhibited almost no antimicrobial effect under the same condition (viability >99%; **Figures S10e-h**).

The results of alive bacteria with no further incubation after photoexcitation showed that i) the additional incubation time was necessary to effectively make the PDT action, leading to eliminate bacteria; ii) there was no other effect (e.g., photothermal effect) to kill bacteria; iii) there was no generated ROS or other effect, bacteria alone without material treatment were still alive even after the additional incubation; iv) these results also confirmed that the materials were successfully coated with the antibody. In other words, the ROS can be generated immediately after photoexcitation, but the PDT action would need several hours to make the generated ROS to induce DNA damage, inactivate enzymes, and oxidize amino acids, eventually leading to destroy or eliminate bacteria.

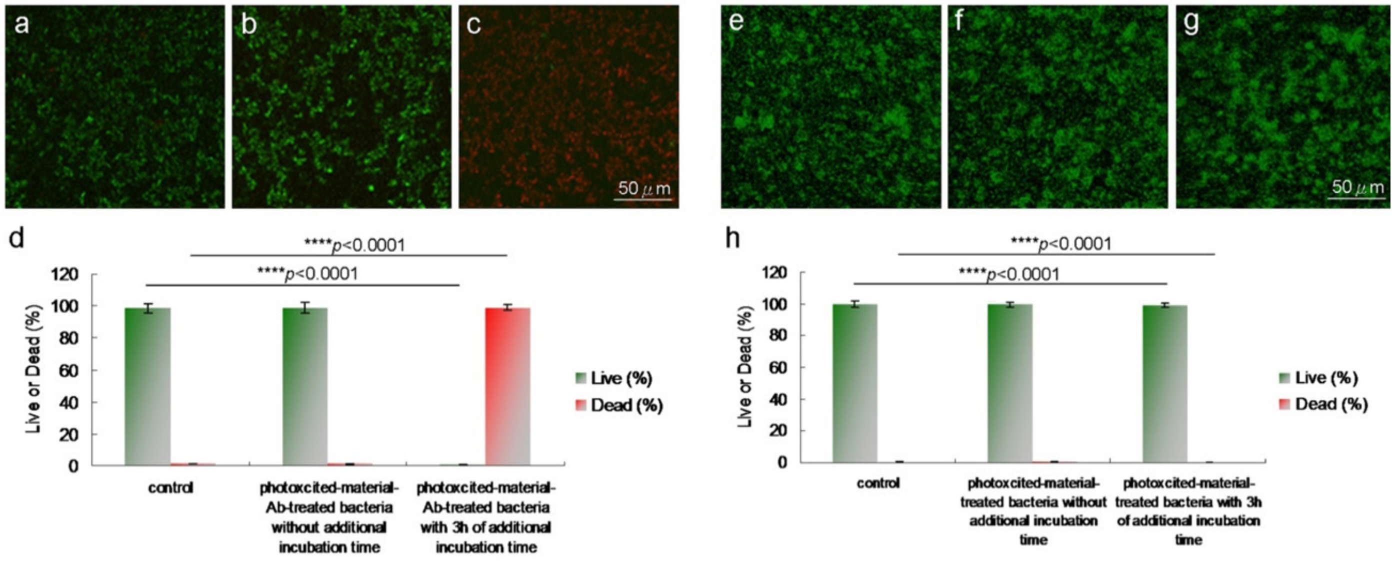

Figure S10. Images obtained after laser photoexcitation exposure (222.7 nJ pixel^{-1} with 170 scans; with a

total effective illumination of ~1.11 s; Ex: 960 nm) of (a,e) bacteria alone with 3h of additional incubation; (b) amino-N-GQD 12.0–Ab_{LPS}-treated bacteria or (f) amino-N-GQD 12.0-treated bacteria without incubation time and (c,g) with 3h of additional incubation. The Live/Dead kit was used to stain bacteria before images were obtained. (d,h) Viability (%) determination results. Delivered dose: OD_{600}:~0.05 of *E. coli* or 0.75 μg mL^{-1} materials. For the percentages alive and dead, ****$p< 0.0001$. *p value obtained using Student's t test (*$p < 0.05$, **$p< 0.01$, ***$p< 0.001$, ****$p< 0.0001$). Data are presented as mean ± SD (n=6).

Table S3. Amount of ROS generated by conducting TPE (222.7 nJ pixel^{-1} with 20, 100 and 170 scans; Ex: 960 nm) to amino-N-GQD 9.1 and amino-N-GQD 9.9 was monitored, respectively [5,8-13]. Delivered dose: 0.75 μg mL^{-1} for material. Data are means±SD (*n*=6).

			$^1O_2$ (by SOSG)[c]				
	Negative control[ac]	ROS neutralization[abc]	Positive control[cd]	amino-N-GQD 9.1	ROS neutralization[bc]	amino-N-GQD 9.9	ROS neutralization[bc]
20 scans	230±8	231±9	1866±74	335±21	232±10	370±26	233±10
100 scans	231±6	232±10	2102±82	919±40	233±9	985±45	232±10
170 scans	233±8	232±9	2254±87	1120±52	233±12	1214±61	233±13

			$^1O_2$ (by *t*-MVP)[e]				
	Negative control[ae]	ROS neutralization[abe]	Positive control[de]	amino-N-GQD 9.1	ROS neutralization[be]	amino-N-GQD 9.9	ROS neutralization[be]
20 scans	318±16	317±16	6002±128	452±26	317±15	498±33	317±13
100 scans	317±19	318±18	6185±153	3310±98	316±14	3942±109	316±15
170 scans	318±18	319±20	8077±166	4536±123	318±15	5104±142	316±14-

			$O_2 \bullet^-$ (by XTT)[f]				
	Negative control[af]	ROS neutralization[abf]	Positive Control[df]	amino-N-GQD 9.1	ROS neutralization[bf]	amino-N-GQD 9.9	ROS neutralization[bf]
20 scans	0	0	1.60±0.08	0.05±0.02	0.02±0.01	0.08.±0.02	0.02±0.01
100 scans	0	0	1.92±0.12	0.51±0.05	0.02±0.01	0.75.±0.05	0.03±0.01
170 scans	0	0	2.30±0.16	0.80±0.07	0.02±0.01	1.09±0.08	0.02±0.01

			$O_2 \bullet^-$ (by GSH)[g]				
	Negative control[ag]	ROS neutralization[abg]	Positive Control[dg]	amino-N-GQD 9.1	ROS neutralization[bg]	amino-N-GQD 9.9	ROS neutralization[bg]
20 scans	0	0	84.8±3.9%	4.0±0.9%	0.2±0.1%	5.0±1.2%	0.2±0.1%
100 scans	0	0	90.2±4.3%	56.5±2.0%	0.1±0.1%	65.3±3.3%	0.2±0.1%
170 scans	0	0	96.8±4.6%	70.1±3.3%	0.1±0.1%	77.9±3.9%	0.2±0.1%

			H_2O_2, OH^-, $ONOO^-$ (by H_2DCFDA)[h]				
	Negative Control[ah]	ROS neutralization[abh]	Positive Control[dh)]	amino-N-GQD 9.1	ROS Neutralization[bh]	amino-N-GQD 9.9	ROS Neutralization[bh]
20 scans	75±2	74±3	3209±66	383±24	75±3	470±26	72±2
100 scans	72±3	72±5	3443±71	559±31	74±5	613±34	73±4
170 scans	75±6	75±3	3790±82	804±39	75±4	862±39	73±4

[a]Negative control: only treat using reagent and laser radiation without using any material (0 μg mL^{-1}).

[b]ROS neutralization: with the treatments of nanomaterial, the laser irradiation and 30 ppm of antioxidant α-Tocopherol/methyl linoleate.

[c]SOSG reagent (Ex/Em: 488/525 nm) has a specific reactivity to generate fluorescence recorded by a PL spectrometer.

[d]Positive control: the treatment of 50 μM *tert*-butyl hydroperoxide (TBHP) and laser irradiation.

[e]*t*-MVP (Ex/Em: 352/465 nm) can react with $^1O_2$, forming a dioxetane intermediate that generates fluorescence upon decomposition to 1-pyrenecarboxaldehyde, and monitored by a PL spectrometer.

[f]XTT would interact with $O_2^{\cdot-}$ and produce the XTT-formazan generating strong absorption (470 nm in wavelength).

[g]GSH containing a thiol-tripeptide can prevent damages to cellular or bacterial components caused by stress of oxidation. Thiol group from GSH can be oxidized to disulfide bond converting GSH to glutathione disulfide. GSH oxidation was used to determine the generated $O_2^{\cdot -}$. Loss of GSH (%) = (absorbance difference between of sample and negative control / absorbance of negative control) × 100 %.
[h]H_2DCFDA passes through cell membranes and converts itself into 2′,7′-dichlorodihydrofluorescin (DCFH). In the presenceof H_2O_2, OH· and $ONOO^-$, DCFH is oxidized to dichlorodihydrofluorescein (DCF), which emits green fluorescence (Ex/Em: 498/522 nm). A spectrophotometer was employed for measurements.

Table S4. Amount of ROS generated by conducting TPE (222.7 nJ pixel^{-1} with 20, 100 and 170 scans; Ex: 960 nm) to amino-N-GQD 11.1 and amino-N-GQD 12.0 was monitored, respectively [5,8-13]. Delivered dose: 0.75 μg mL^{-1} for material. Data are means±SD (*n*=6).

			$^1O_2$ (by SOSG)[c]				
	Negative control[ac]	ROS neutralization[abc]	Positive control[cd]	amino-N-GQD 11.1	ROS neutralization[bc]	amino-N-GQD 12.0	ROS neutralization[bc]
20 scans	232±9	232±10	1890±78	405±24	233±9	449±28	234±10
100 scans	230±10	231±9	2122±85	1013±45	232±10	1062±47	233±9
170 scans	231±9	233±10	2330±88	1207±58	234±10	1298±67	233±11

			$^1O_2$ (by *t*-MVP)[e]				
	Negative control[ae]	ROS neutralization[abe]	Positive control[de]	amino-N-GQD 11.1	ROS neutralization[be]	amino-N-GQD 12.0	ROS neutralization[be]
20 scans	320±15	320±17	6030±132	547±29	320±14	602±37	319±15
100 scans	319±20	319±19	7028±150	3940±108	319±16	4633±118	318±16
170 scans	319±19	321±20	8015±173	5052±135	320±16	5486±145	320±15-

			$O_2 \bullet^-$ (by XTT)[f]				
	Negative control[af]	ROS neutralization[abf]	Positive Control[df]	amino-N-GQD 11.1	ROS neutralization[bf]	amino-N-GQD 12.0	ROS neutralization[bf]
20 scans	0	0	1.65±0.09	0.15±0.04	0.02±0.01	0.21.±0.05	0.02±0.01
100 scans	0	0	1.96±0.14	0.60±0.06	0.02±0.01	0.83.±0.06	0.03±0.01
170 scans	0	0	2.43±0.17	0.97±0.09	0.03±0.01	1.20±0.10	0.03±0.01

			$O_2 \bullet^-$ (by GSH)[g]				
	Negative control[ag]	ROS neutralization[abg]	Positive Control[dg]	amino-N-GQD 11.1	ROS neutralization[bg]	amino-N-GQD 12.0	ROS neutralization[bg]
20 scans	0	0	85.2±4.0%	7,1±1.0%	0.2±0.1%	8.2±1.4%	0.2±0.1%
100 scans	0	0	93.1±4.5%	64.2±2.9%	0.2±0.1%	72.9±3.8%	0.1±0.1%
170 scans	0	0	97.9±4.8%	76.8±3.6%	0.1±0.1%	82.1±4.1%	0.2±0.1%

			H_2O_2, OH^-, $ONOO^-$ (by H_2DCFDA)[h]				
	Negative Control[ah]	ROS neutralization[abh]	Positive Control[dh]	amino-N-GQD 11.1	ROS Neutralization[bh]	amino-N-GQD 12.0	ROS Neutralization[bh]
20 scans	76±5	75±2	3222±68	523±30	75±3	609±32	72±2
100 scans	74±4	73±5	3454±76	640±35	74±5	736±39	73±4
170 scans	72±5	73±4	3803±84	927±44	75±4	1188±46	73±4

[a]Negative control: only treat using reagent and laser radiation without using any material (0 μg mL^{-1}).

[b]ROS neutralization: with the treatments of nanomaterial, the laser irradiation and 30 ppm of antioxidant α-Tocopherol/methyl linoleate.

[c]SOSG reagent (Ex/Em: 488/525 nm) has a specific reactivity to generate fluorescence recorded by a PL spectrometer.

[d]Positive control: the treatment of 50 μM TBHP and laser irradiation.

[e]*t*-MVP (Ex/Em: 352/465 nm) can react with $^1O_2$, forming a dioxetane intermediate that generates fluorescence upon decomposition to 1-pyrenecarboxaldehyde, and monitored by a PL spectrometer.

[f]XTT would interact with $O_2^{\cdot-}$ and produce the XTT-formazan generating strong absorption (470 nm in wavelength).

[g]GSH containing a thiol-tripeptide can prevent damages to cellular or bacterial components caused by stress of oxidation. Thiol group from GSH can be oxidized to disulfide bond converting GSH to glutathione disulfide. GSH oxidation was used to determine the generated $O_2^{\cdot -}$. Loss of GSH (%) = (absorbance difference between of sample and negative control / absorbance of negative control) × 100 %.

[h]H_2DCFDA passes through cell membranes and converts itself into DCFH. In the presenceof H_2O_2, OH· and $ONOO^-$, DCFH is oxidized to DCF, which emits green fluorescence (Ex/Em: 498/522 nm). A spectrophotometer was employed for measurements.

Table S5. Amount of ROS generated by conducting TPE (222.7 nJ pixel^{-1} with 20, 100 and 170 scans; Ex: 960 nm) to amino-N-GQD 9.1–Ab_{LPS}- and amino-N-GQD 9.9–Ab_{LPS}-treated-*E. coli* was monitored, respectively [5,8-13]. Delivered dose: OD_{600} = ~0.05 for *E. coli* and 0.75 μg mL^{-1} for material. Data are means±SD (*n*=6).

			$^1O_2$ (by SOSG)[c]				
	Negative control[ac]	ROS neutralization[abc]	Positive control[cd]	amino-N-GQD 9.1	ROS neutralization[bc]	amino-N-GQD 9.9	ROS neutralization[bc]
20 scans	232±7	232±10	1834±71	248±15	233±11	279±22	234±9
100 scans	232±5	232±9	2069±80	826±35	232±10	881±39	233±11
170 scans	235±9	234±11	2201±86	993±48	234±14	1095±56	234±13
			$^1O_2$ (by *t*-MVP)[e]				
	Negative control[ae]	ROS neutralization[abe]	Positive control[de]	amino-N-GQD 9.1	ROS neutralization[be]	amino-N-GQD 9.9	ROS neutralization[be]
20 scans	316±18	317±15	5972±125	365±22	316±14	403±28	315±14
100 scans	316±20	315±19	6145±149	3219±94	316±13	3860±101	315±16
170 scans	317±20	318±21	8030±163	4454±118	316±16	4989±136	317±15-
			$O_2^{\bullet -}$ (by XTT)[f]				
	Negative control[af]	ROS neutralization[abf]	Positive Control[df]	amino-N-GQD 9.1	ROS neutralization[bf]	amino-N-GQD 9.9	ROS neutralization[bf]
20 scans	0	0	1.53±0.08	0.03±0.02	0.02±0.01	0.06.±0.02	0.02±0.01
100 scans	0	0	1.87±0.11	0.48±0.04	0.03±0.01	0.71.±0.04	0.02±0.01
170 scans	0	0	2.27±0.15	0.77±0.05	0.02±0.01	1.02±0.07	0.03±0.01
			$O_2^{\bullet -}$ (by GSH)[g]				
	Negative control[ag]	ROS neutralization[abg]	Positive Control[dg]	amino-N-GQD 9.1	ROS neutralization[bg]	amino-N-GQD 9.9	ROS neutralization[bg]
20 scans	0	0	84.1±3.7%	3.3±0.9%	0.1±0.1%	4.2±1.1%	0.2±0.1%
100 scans	0	0	89.6±4.0%	55.4±1.9%	0.2±0.1%	64.7±3.2%	0.2±0.1%
170 scans	0	0	96.3±4.5%	68.9±3.0%	0.2±0.1%	77.0±3.5%	0.3±0.1%
			H_2O_2, OH^-, $ONOO^-$ (by H_2DCFDA)[h]				
	Negative Control[ah]	ROS neutralization[abh]	Positive Control[dh]	amino-N-GQD 9.1	ROS Neutralization[bh]	amino-N-GQD 9.9	ROS Neutralization[bh]
20 scans	76±3	75±5	3180±63	229±15	73±4	315±20	73±4
100 scans	74±4	74±2	3422±67	385±27	75±3	452±32	75±5
170 scans	75±3	74±5	3773±77	629±32	72±5	708±36	74±5

[a]Negative control: only treat using reagent and laser radiation without using any material (0 μg mL^{-1}).

[b]ROS neutralization: with the treatments of nanomaterial, the laser irradiation and 30 ppm of antioxidant *α*-Tocopherol/methyl linoleate.

[c]SOSG reagent (Ex/Em: 488/525 nm) has a specific reactivity to generate fluorescence recorded by a PL spectrometer.

[d]Positive control: the treatment of 50 μM TBHP and laser irradiation.

[e]*t*-MVP (Ex/Em: 352/465 nm) can react with $^1O_2$, forming a dioxetane intermediate that generates fluorescence upon decomposition to 1-pyrenecarboxaldehyde, and monitored by a PL spectrometer.

[f]XTT would interact with $O_2^{\cdot -}$ and produce the XTT-formazan generating strong absorption (470 nm in wavelength).

[g]GSH containing a thiol-tripeptide can prevent damages to cellular or bacterial components caused by stress of oxidation. Thiol group from GSH can be oxidized to disulfide bond converting GSH to glutathione disulfide. GSH oxidation was used to determine the generated $O_2^{\cdot -}$. Loss of GSH (%) = (absorbance difference between of sample and negative control / absorbance of negative control) × 100 %.

[h]H_2DCFDA passes through cell membranes and converts itself into DCFH. In the presenceof H_2O_2, OH· and $ONOO^-$, DCFH is oxidized to DCF, which emits green fluorescence (Ex/Em: 498/522 nm). A spectrophotometer was employed for measurements.

Table S6. Amount of ROS generated by conducting TPE (222.7 nJ pixel^{-1} with 20, 100 and 170 scans; Ex: 960 nm) to amino-N-GQD 11.1–Ab_{LPS}- and amino-N-GQD 12.0–Ab_{LPS}-treated-*E. coli* was monitored, respectively [5,8-13]. Delivered dose: OD_{600} = ~0.05 for *E. coli* and 0.75 μg mL^{-1} for material. Data are means±SD (*n*=6).

			$^1O_2$ (by SOSG)[c]				
	Negative control[ac]	ROS neutralization[abc]	Positive control[cd]	amino-N-GQD 11.1	ROS neutralization[bc]	amino-N-GQD 12.0	ROS neutralization[bc]
20 scans	234±8	233±11	1872±76	313±26	232±13	358±30	233±12
100 scans	235±7	234±11	1991±83	921±40	234±15	974±42	233±11
170 scans	236±9	236±13	2254±89	1088±53	234±14	1201±61	235±13
			$^1O_2$ (by *t*-MVP)[e]				
	Negative control[ae]	ROS neutralization[abe]	Positive control[de]	amino-N-GQD 11.1	ROS neutralization[be]	amino-N-GQD 12.0	ROS neutralization[be]
20 scans	314±17	315±16	6004±129	455±31	314±15	496±34	316±15
100 scans	314±18	313±18	7007±143	3855±102	314±13	4547±115	315±13
170 scans	315±17	314±18	7985±160	4961±127	316±16	5390±142	314±14

			$O_2 \bullet^-$ (by XTT)[f]				
	Negative control[af]	ROS neutralization[abf]	Positive Control[df]	amino-N-GQD 11.1	ROS neutralization[bf]	amino-N-GQD 12.0	ROS neutralization[bf]
20 scans	0	0	1.48±0.07	0.10±0.03	0.02±0.01	0.15±0.04	0.01±0.01
100 scans	0	0	1.92±0.11	0.55±0.04	0.02±0.02	0.76±0.05	0.02±0.01
170 scans	0	0	2.40±0.16	0.92±0.07	0.03±0.01	1.14±0.09	0.02±0.01
			$O_2 \bullet^-$ (by GSH)[g]				
	Negative control[ag]	ROS neutralization[abg]	Positive Control[dg]	amino-N-GQD 11.1	ROS neutralization[bg]	amino-N-GQD 12.0	ROS neutralization[bg]
20 scans	0	0	85.5±4.1%	6.4±1.2%	0.2±0.1%	7.7±1.3%	0.2±0.1%
100 scans	0	0	92.6±4.3%	63.6±2.9%	0.1±0.1%	71.4±3.1%	0.2±0.1%
170 scans	0	0	97.4±4.6%	75.2±3.3%	0.2±0.1%	80.7±3.6%	0.2±0.1%
			H_2O_2, OH^-, $ONOO^-$ (by H_2DCFDA)[h]				
	Negative Control[ah]	ROS neutralization[abh]	Positive Control[dh]	amino-N-GQD 11.1	ROS Neutralization[bh]	amino-N-GQD 12.0	ROS Neutralization[bh]
20 scans	73±5	74±3	3200±62	275±27	74±4	448±28	74±3
100 scans	75±3	74±4	3426±71	392±31	75±5	581±34	73±4
170 scans	75±4	76±5	3758±80	769±39	73±5	1035±40	76±4

[a]Negative control: only treat using reagent and laser radiation without using any material (0 μg mL^{-1}).

[b]ROS neutralization: with the treatments of nanomaterial, the laser irradiation and 30 ppm of antioxidant *α*-Tocopherol/methyl linoleate.

[c]SOSG reagent (Ex/Em: 488/525 nm) has a specific reactivity to generate fluorescence recorded by a PL spectrometer.

[d]Positive control: the treatment of 50 μM TBHP and laser irradiation.

[e]*t*-MVP (Ex/Em: 352/465 nm) can react with $^1O_2$, forming a dioxetane intermediate that generates fluorescence upon decomposition to 1-pyrenecarboxaldehyde, and monitored by a PL spectrometer.

[f]XTT would interact with $O_2^{\cdot-}$ and produce the XTT-formazan generating strong absorption (470 nm in wavelength).

[g]GSH containing a thiol-tripeptide can prevent damages to cellular or bacterial components caused by stress of oxidation. Thiol group from GSH can be oxidized to disulfide bond converting GSH to glutathione disulfide. GSH oxidation was used to determine the generated $O_2^{\cdot-}$. Loss of GSH (%) = (absorbance difference between of sample and negative control / absorbance of negative control) × 100 %.

[h]H_2DCFDA passes through cell membranes and converts itself into DCFH. In the presenceof H_2O_2, OH· and $ONOO^-$, DCFH is oxidized to DCF, which emits green fluorescence (Ex/Em: 498/522 nm). A spectrophotometer was employed for measurements.

Table S7. Amount of ROS generated by conducting TPE (222.7 nJ pixel^{-1} with 20, 100 and 170 scans; Ex: 960 nm) to amino-N-GQD 9.1- and amino-N-GQD 9.9-treated-*E. coli* was monitored, respectively [5,8-13]. Delivered dose: OD600 = ~0.05 for *E. coli* and 0.75 μg mL^{-1} for material. Data are means±SD (*n*=6).

			$^1O_2$ (by SOSG)[c]				
	Negative control[ac]	ROS neutralization[abc]	Positive control[cd]	amino-N-GQD 9.1	ROS neutralization[bc]	amino-N-GQD 9.9	ROS neutralization[bc]
20 scans	234±6	234±9	1852±69	236±10	235±9	248±18	235±10
100 scans	232±6	233±8	2084±81	440±28	235±10	475±30	234±12
170 scans	235±10	236±9	2225±88	512±35	236±11	582±38	236±14
			$^1O_2$ (by *t*-MVP)[e]				
	Negative control[ae]	ROS neutralization[abe]	Positive control[de]	amino-N-GQD 9.1	ROS neutralization[be]	amino-N-GQD 9.9	ROS neutralization[be]
20 scans	317±15	318±15	5998±127	301±15	317±13	372±25	315±13
100 scans	318±15	318±18	6172±150	1035±62	318±15	1154±66	316±18
170 scans	317±19	317±20	8055±168	1270±75	315±16	1331±78	316±15-
			$O_2\bullet^-$ (by XTT)[f]				
	Negative control[af]	ROS neutralization[abf]	Positive Control[df]	amino-N-GQD 9.1	ROS neutralization[bf]	amino-N-GQD 9.9	ROS neutralization[bf]
20 scans	0	0	1.55±0.08	0.02±0.01	0.02±0.01	0.03.±0.01	0.02±0.01
100 scans	0	0	1.91±0.12	0.24±0.02	0.04±0.01	0.35.±0.05	0.03±0.01
170 scans	0	0	2.30±0.16	0.39±0.04	0.03±0.01	0.47±0.09	0.02±0.01
			$O_2\bullet^-$ (by GSH)[g]				
	Negative control[ag]	ROS neutralization[abg]	Positive Control[dg]	amino-N-GQD 9.1	ROS neutralization[bg]	amino-N-GQD 9.9	ROS neutralization[bg]
20 scans	0	0	84.0±3.3%	1.5±0.3%	0.2±0.1%	2.2±0.6%	0.2±0.1%
100 scans	0	0	90.1±4.0%	26.2±1.1%	0.3±0.1%	30.8±1.3%	0.4±0.1%
170 scans	0	0	95.2±4.2%	35.9±1.5%	0.3±0.1%	41.1±1.7%	0.2±0.1%
			H_2O_2, OH^-, $ONOO^-$ (by H_2DCFDA)[h]				
	Negative Control[ah]	ROS neutralization[abh]	Positive Control[dh]	amino-N-GQD 9.1	ROS Neutralization[bh]	amino-N-GQD 9.9	ROS Neutralization[bh]
20 scans	72±5	74±5	3197±61	126±10	75±4	138±12	74±3
100 scans	76±3	72±5	3405±64	202±18	74±5	230±20	74±6
170 scans	74±3	75±4	3752±73	319±24	75±3	405±29	76±5

[a]Negative control: only treat using reagent and laser radiation without using any material (0 μg mL^{-1}).

[b]ROS neutralization: with the treatments of nanomaterial, the laser irradiation and 30 ppm of antioxidant *α*-Tocopherol/methyl linoleate.

[c]SOSG reagent (Ex/Em: 488/525 nm) has a specific reactivity to generate fluorescence recorded by a PL spectrometer.

[d]Positive control: the treatment of 50 μM TBHP and laser irradiation.

[e]*t*-MVP (Ex/Em: 352/465 nm) can react with $^1O_2$, forming a dioxetane intermediate that generates fluorescence upon decomposition to 1-pyrenecarboxaldehyde, and monitored by a PL spectrometer.

[f]XTT would interact with $O_2^{\cdot-}$ and produce the XTT-formazan generating strong absorption (470 nm in wavelength).

[g]GSH containing a thiol-tripeptide can prevent damages to cellular or bacterial components caused by stress of oxidation. Thiol group from GSH can be oxidized to disulfide bond converting GSH to glutathione disulfide. GSH oxidation was used to determine the generated $O_2^{\cdot -}$. Loss of GSH (%) = (absorbance difference between of sample and negative control / absorbance of negative control) × 100 %.
[h]H_2DCFDA passes through cell membranes and converts itself into DCFH. In the presenceof H_2O_2, OH· and $ONOO^-$, DCFH is oxidized to DCF, which emits green fluorescence (Ex/Em: 498/522 nm). A spectrophotometer was employed for measurements.

Table S8. Amount of ROS generated by conducting TPE (222.7 nJ pixel^{-1} with 20, 100 and 170 scans; Ex: 960 nm) to amino-N-GQD 11.1- and amino-N-GQD 12.0-treated-*E. coli* was monitored, respectively [5,8-13]. Delivered dose: OD600 = ~0.05 for *E. coli* and 0.75 μg mL^{-1} for material. Data are means±SD (*n*=6).

			$^1O_2$ (by SOSG)[c]				
	Negative control[ac]	ROS neutralization[abc]	Positive control[cd]	amino-N-GQD 11.1	ROS neutralization[bc]	amino-N-GQD 12.0	ROS neutralization[bc]
20 scans	233±8	235±10	1889±77	259±16	233±12	297±20	233±13
100 scans	235±8	235±10	2004±82	486±31	235±16	505±32	232±13
170 scans	234±7	237±11	2265±85	551±34	233±13	620±39	234±14
			$^1O_2$ (by *t*-MVP)[e]				
	Negative control[ae]	ROS neutralization[abe]	Positive control[de]	amino-N-GQD 11.1	ROS neutralization[be]	amino-N-GQD 12.0	ROS neutralization[be]
20 scans	315±16	316±16	6025±132	323±22	315±14	390±28	315±16
100 scans	315±17	316±15	7030±146	1089±65	315±16	1182±40	315±15
170 scans	316±15	317±19	8012±165	1305±80	314±14	1365±83	317±15
			$O_2\bullet^-$ (by XTT)[f]				
	Negative control[af]	ROS neutralization[abf]	Positive Control[df]	amino-N-GQD 11.1	ROS neutralization[bf]	amino-N-GQD 12.0	ROS neutralization[bf]
20 scans	0	0	1.49±0.09	0.04±0.01	0.03±0.01	0.08±0.03	0.02±0.01
100 scans	0	0	1.95±0.13	0.27±0.04	0.03±0.01	0.39±0.06	0.03±0.01
170 scans	0	0	2.43±0.18	0.45±0.05	0.02±0.02	0.52±0.10	0.04±0.01
			$O_2\bullet^-$ (by GSH)[g]				
	Negative control[ag]	ROS neutralization[abg]	Positive Control[dg]	amino-N-GQD 11.1	ROS neutralization[bg]	amino-N-GQD 12.0	ROS neutralization[bg]
20 scans	0	0	85.9±4.3%	2.5±0.4%	0.2±0.2%	3.6±0.5%	0.2±0.1%
100 scans	0	0	93.4±4.7%	29.8±1.5%	0.3±0.1%	36.5±1.8%	0.1±0.1%
170 scans	0	0	98.0±5.0%	40.1±2.2%	0.3±0.1%	45.2±2.4%	0.3±0.1%
			H_2O_2, OH^-, $ONOO^-$ (by H_2DCFDA)[h]				
	Negative Control[ah]	ROS neutralization[abh]	Positive Control[dh]	amino-N-GQD 11.1	ROS Neutralization[bh]	amino-N-GQD 12.0	ROS Neutralization[bh]
20 scans	71±4	73±2	3190±60	140±11	74±5	170±15	73±5
100 scans	74±2	75±5	3392±69	222±20	76±5	255±23	74±4
170 scans	73±5	75±6	3728±78	345±26	74±6	419±31	73±2

[a]Negative control: only treat using reagent and laser radiation without using any material (0 μg mL^{-1}).

[b]ROS neutralization: with the treatments of nanomaterial, the laser irradiation and 30 ppm of antioxidant *α*-Tocopherol/methyl linoleate.

[c]SOSG reagent (Ex/Em: 488/525 nm) has a specific reactivity to generate fluorescence recorded by a PL spectrometer.

[d]Positive control: the treatment of 50 μM TBHP and laser irradiation.

[e]*t*-MVP (Ex/Em: 352/465 nm) can react with $^1O_2$, forming a dioxetane intermediate that generates fluorescence upon decomposition to 1-pyrenecarboxaldehyde, and monitored by a PL spectrometer.

[f]XTT would interact with $O_2^{\cdot -}$ and produce the XTT-formazan generating strong absorption (470 nm in wavelength).

[g]GSH containing a thiol-tripeptide can prevent damages to cellular or bacterial components caused by stress of oxidation. Thiol group from GSH can be oxidized to disulfide bond converting GSH to glutathione disulfide. GSH oxidation was used to determine the generated $O_2^{\cdot -}$. Loss of GSH (%) = (absorbance difference between of sample and negative control / absorbance of negative control) × 100 %.

[h]H_2DCFDA passes through cell membranes and converts itself into DCFH. In the presenceof H_2O_2, OH· and $ONOO^-$, DCFH is oxidized to DCF, which emits green fluorescence (Ex/Em: 498/522 nm). A spectrophotometer was employed for measurements.

The experiment could have been compromised had the sorted amino-N-GQD samples been exposed to white light. To prevent the possibility of confounding the PDT process by inadvertently exposing the amino-N-GQD samples to white light, the experiments related to PDT were conducted in darkness. A low dose (0.75 μg mL^{-1}) of sorted amino-N-GQDs–Ab_{LPS} that had been incubated for 3 h with *E. coli* (OD_{600}: ~0.05) at 37 °C in the dark was employed in all subsequent experiments.

In evaluations of the material's antimicrobial potential, this study conducted PDT experiments against *E. coli* by using TPE (222.7 nJ $pixel^{-1}$ with 20 scans). In addition, the effect on the viability of *E. coli* treated with photoexcited material–Ab_{LPS} was determined. No obviously bactericidal effects were observed toward all panels (**Figure S11a,b**). After a low TPE energy and a short photoexcitation time, the viability was relatively high (approximately 96% for the amino-N-GQD 9.1–Ab_{LPS}, approximately 94% for the amino-N-GQD 9.9–Ab_{LPS}, approximately 93% for the amino-N-GQD 11.1–Ab_{LPS}, and 91% for the amino-N-GQD 12.0–Ab_{LPS} (**Figure S11a**). This value corresponded to an approximately 0.022, 0.030, 0.034 and 0.042 log_{10} reduction with size increased (**Figure S11b**), and the viabilities was corresponding to the images in **Figures 3f and 4b**. Bacterial viability was higher for materials without antibody coating (over 98% viability for all sorted dots) than that for those with the coating (**Figure S11a,b** corresponding to the images in **Figures 3j and 4e**. After TPE (222.7 nJ $pixel^{-1}$ with 100 scans), the viability was relatively low (approximately 11% for the amino-N-GQD 9.1–Ab_{LPS}, approximately 7% for the amino-N-GQD 9.9–Ab_{LPS}, approximately 2% for the amino-N-GQD 11.1–Ab_{LPS}, and 0% for the amino-N-GQD 12.0–Ab_{LPS} (**Figure S11c**). This value corresponded to an approximately 0.959-7.754 log_{10} reduction (**Figure S11d**). The viabilities in **Figure S11c,d** were also consistent with the TPL and TEM images in **Figures 3g and 4c**. However, the amino-N-GQD–Ab_{LPS}-treated bacteria were completely eliminated (100% elimination; **Figure S11e,f** corresponding to the images in **Figures 3h and 4d**) with an increase to 170 scans under TPE (222.7 nJ $pixel^{-1}$), although antimicrobial capabilities were still not apparent (approximately 6%,8%, 9%, and 11% elimination for the amino-N-GQD 9.1-, amino-N-GQD 9.9-, amino-N-GQD 11.1-, and amino-N-GQD 12.0-without coating antibody, respectively; **Figure S11e,f** corresponding to the images in **Figures 3k and 4f**). Subsequently, this study investigated whether bacterial viability was affected by the N-bonding composition and amino functional groups. Additionally, results also demonstrated that the antibody was successfully coated with materials, enhancing the specificity and selectivity.

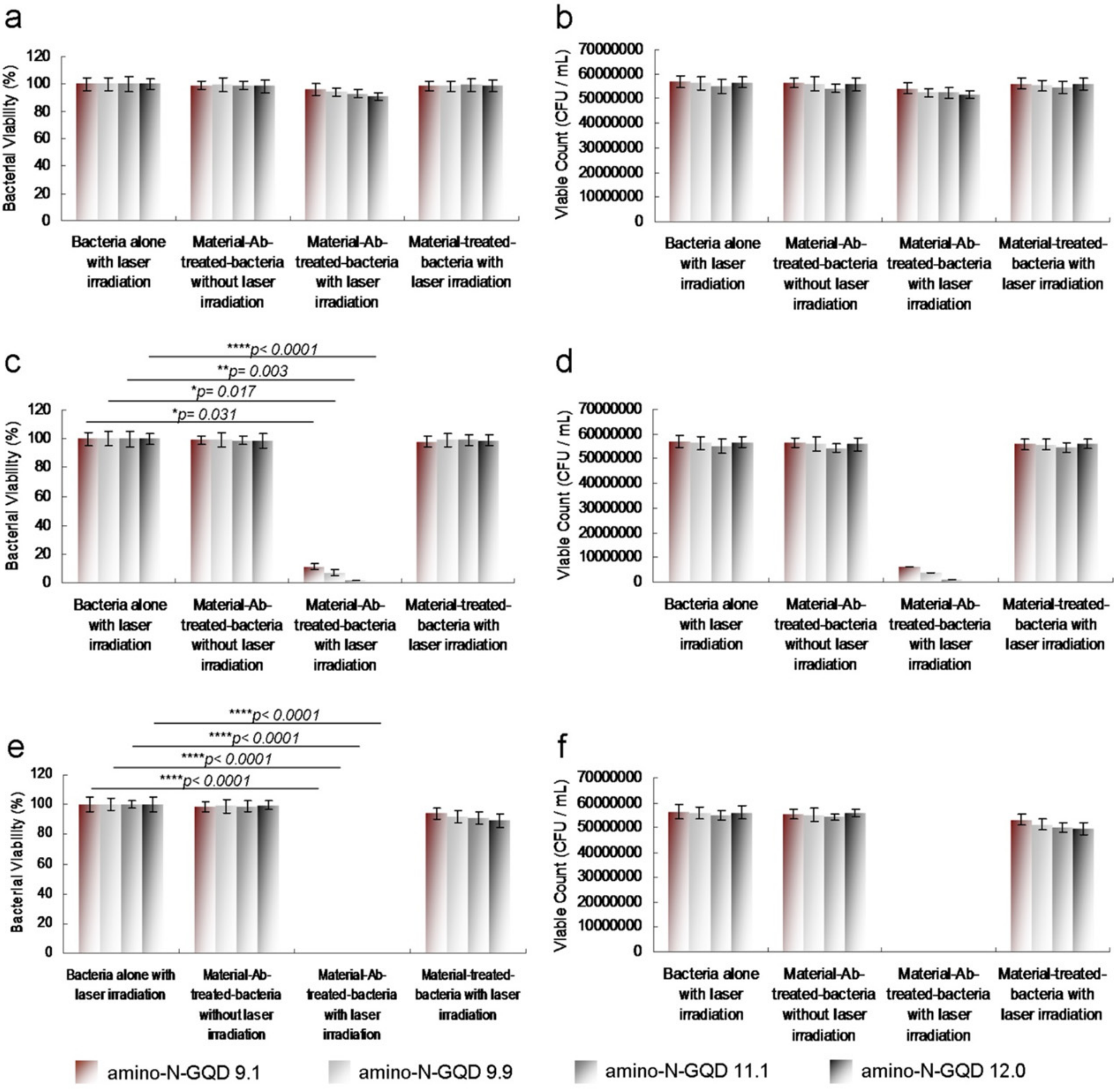

Figure S11. *E. coli* viability (%), determined from the viable count of material–Ab_{LPS}-treated *E. coli* obtained using the CFU assay under a low TPE energy and a short photoexcitation time (222.7 nJ pixel^{-1}; Ex: 960 nm) with (a,b) 20 scans, (c,d) 100 scans, and (e,f) 170 scans. Delivered dose: OD600 = ~0.05 for bacteria and 0.75 μg mL^{-1} for material–Ab_{LPS}. Data are presented as mean±SD (n=6). (c) For amino-N-GQD 9.1–Ab_{LPS}-, amino-N-GQD 9.9–Ab_{LPS}-, amino-N-GQD 11.1–Ab_{LPS}-, and amino-N-GQD 12.0–Ab_{LPS}-treated *E. coli* with photoexcitation, *p= 0.031, *p= 0.017, **p= 0.003, and ****p< 0.0001, respectively. (e) For all material–Ab_{LPS}-treated *E. coli* subjected to TPE, p< 0.0001. *p value calculated using Student's t test (*p < 0.05, **p< 0.01, ***p< 0.001, ****p< 0.0001).

This study employed the experimental template of Gram-positive MRSA The surface protein, namely protein A, on the cell wall of MRSA was considered; thus, the material was coated with an anti-protein A antibody ($Ab_{protein\ A}$) to form a material–$Ab_{protein\ A}$ hybrid, leading to enhance specificity, selectivity, and efficiency. A CFU assay was performed to examine material biocompatibility. The number of bacterial cells was calculated as log_{10} CFU per milliliter (log_{10} CFU mL^{-1}) and is expressed herein as a percentage (**Figure S12**). The executed CFU assay revealed the sorted amino-N-GQDs to be highly biocompatible. Additionally, the toxicity of the material was discovered to contribute to the elimination of bacteria in PDT.

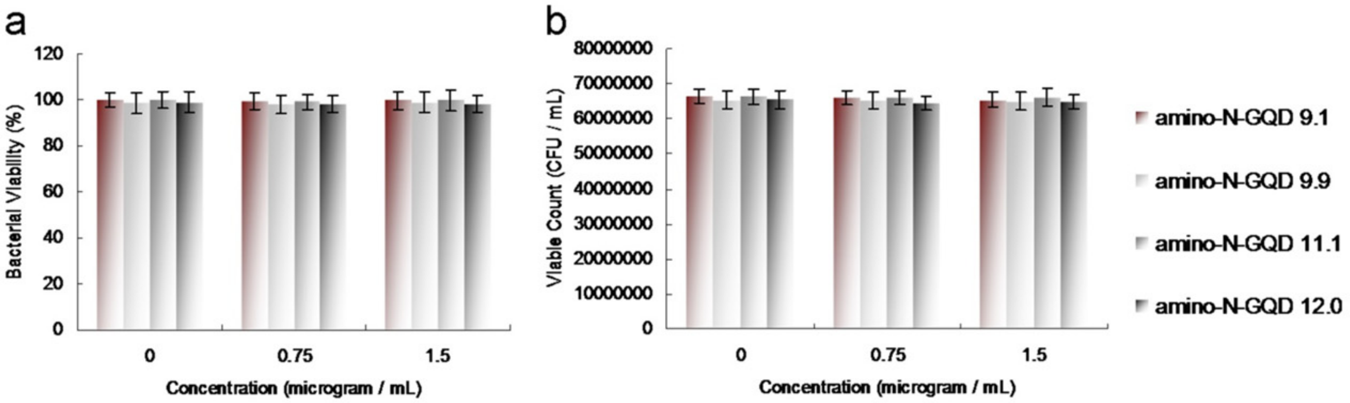

Figure S12. Number of surviving (a,b) the sorted amino-N-GQD–$Ab_{protein\ A}$-treated MRSA cells (unit: CFU mL^{-1}), as determined using a CFU counting assay, and MRSA cells (expressed as a percentage). Delivered dose: OD_{600} = ~0.05 for bacteria and 0–1.5 µg mL^{-1} for material–$Ab_{protein\ A}$. Data are presented as mean±SD (*n*=6).

The experiment could have been compromised had the sorted amino-N-GQD samples been exposed to white light. To prevent the possibility of confounding the PDT process by inadvertently exposing the amino-N-GQD samples to white light, the experiments related to PDT were conducted in darkness. A low dose (0.75 μg mL^{-1}) of sorted amino-N-GQDs–$Ab_{protein\ A}$ that had been incubated for 3 h with MRSA (OD_{600}: ~0.05) at 37 °C in the dark was employed in all subsequent experiments.

In evaluations of the material's antimicrobial potential, this study conducted PDT experiments against MRSA by using TPE (222.7 nJ $pixel^{-1}$ with 20 scans). In addition, the effect on the viability of MRSA treated with photoexcited material–$Ab_{protein\ A}$ was determined. No obviously bactericidal effects were observed toward all panels (**Figure S13a,b**). After a low TPE energy and a short photoexcitation time, the viability was relatively high (approximately 96.5% for the amino-N-GQD 9.1–$Ab_{protein\ A}$, approximately 92% for the amino-N-GQD 9.9–$Ab_{protein\ A}$, approximately 89% for the amino-N-GQD 11.1–$Ab_{protein\ A}$, and 87.5% for the amino-N-GQD 12.0–$Ab_{protein\ A}$ (**Figure S13a**). This value corresponded to an approximately 0.013, 0.026, 0.036 and 0.050 log_{10} reduction with size increased (**Figure S13b**). Bacterial viability was higher for materials without antibody coating (over 98.5% viability for all sorted dots) than that for those with the coating (**Figure S13a,b**). After TPE (222.7 nJ $pixel^{-1}$ with 100 scans), the viability was relatively low (approximately 13% for the amino-N-GQD 9.1–$Ab_{protein\ A}$, approximately 8% for the amino-N-GQD 9.9–$Ab_{protein\ A}$, approximately 1.5% for the amino-N-GQD 11.1–$Ab_{protein\ A}$, and 0% for the amino-N-GQD 12.0–$Ab_{protein\ A}$ (**Figure S13c**). This value corresponded to an approximately 0.895–7.818 log_{10} reduction (**Figure S13d**). However, the amino-N-GQD–$Ab_{protein\ A}$-treated bacteria were completely eliminated (100% elimination; **Figure S13e,f**) with an increase to 170 scans under TPE (222.7 nJ $pixel^{-1}$), although antimicrobial capabilities were still not apparent (approximately 6%, 9%, 10.5%, and 12% elimination for the amino-N-GQD 9.1–, amino-N-GQD 9.9–, amino-N-GQD 11.1–, and amino-N-GQD 12.0–without coating antibody, respectively. Subsequently, this study investigated whether bacterial viability was affected by the N-bonding composition and amino functional groups. Additionally, results also demonstrated that the antibody was successfully coated with materials, enhancing the specificity and selectivity.

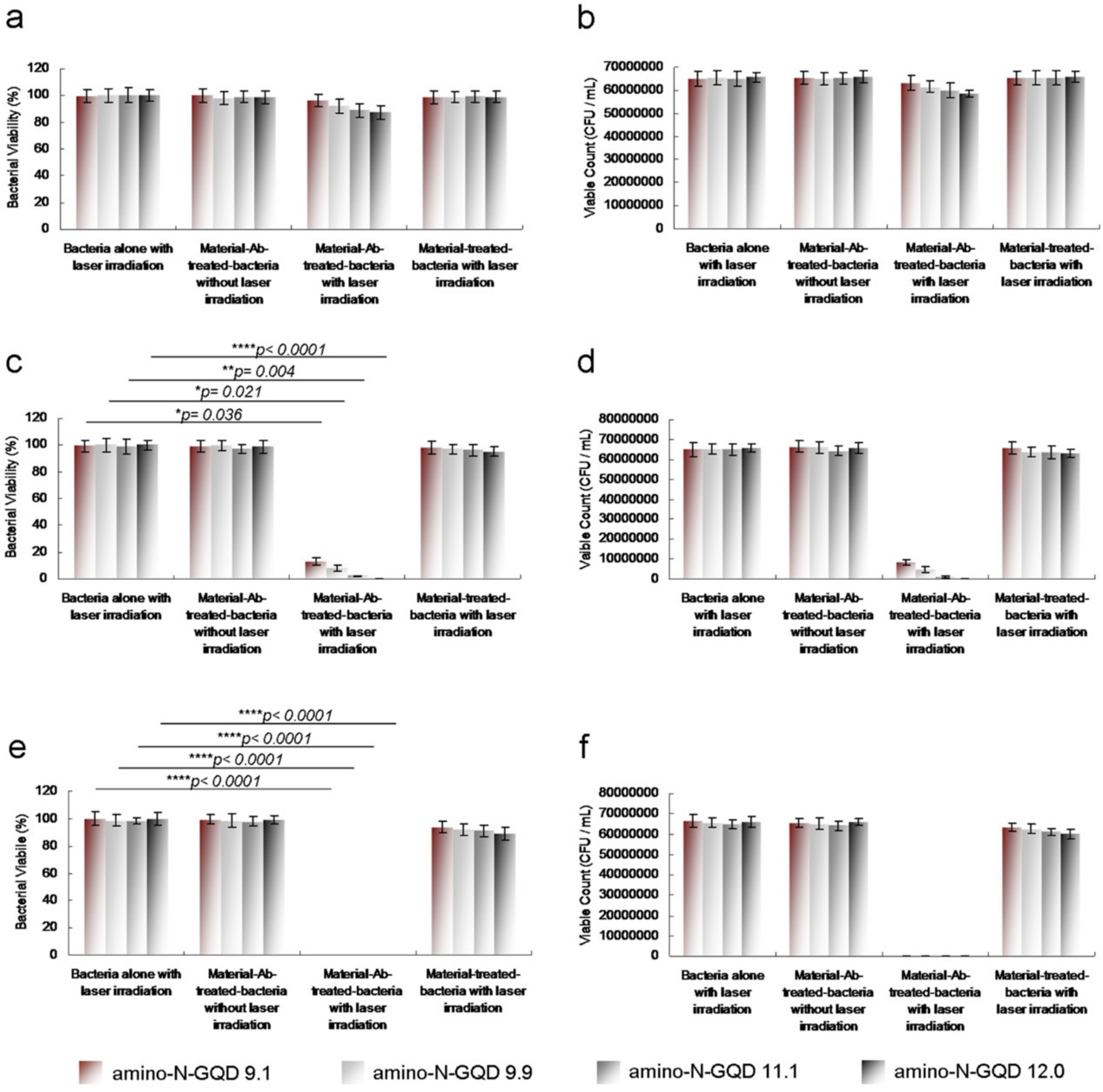

Figure S13. MRSA viability (%), determined from the viable count of material–$Ab_{protein\ A}$-treated MRSA obtained using the CFU assay under a low TPE energy and a short photoexcitation time (222.7 nJ $pixel^{-1}$; Ex: 960 nm) with (a,b) 20 scans, (c,d) 100 scans, and (e,f) 170 scans. Delivered dose: OD600 = ~0.05 for bacteria and 0.75 μg mL^{-1} for material–$Ab_{protein\ A}$. Data are presented as mean±SD (n=6). (c) For amino-N-GQD 9.1–$Ab_{protein\ A}$-, amino-N-GQD 9.9–$Ab_{protein\ A}$-, amino-N-GQD 11.1–$Ab_{protein\ A}$-, and amino-N-GQD 12.0–$Ab_{protein\ A}$-treated MRSA with photoexcitation, *p= 0.036, *p= 0.021, **p= 0.004, and ****p< 0.0001, respectively. (e) For all material–$Ab_{protein\ A}$-treated MRSA subjected to TPE, p< 0.0001. *p value calculated using Student's t test (*p < 0.05, **p< 0.01, ***p< 0.001, ****p< 0.0001).

Table S9. Amount of ROS generated by conducting TPE (222.7 nJ pixel^{-1} with 20, 100 and 170 scans; Ex: 960 nm) to amino-N-GQD 9.1–Ab$_{\text{protein A}}$- and amino-N-GQD 9.9–Ab$_{\text{protein A}}$-treated-MRSA was monitored, respectively [5,8-13]. Delivered dose: OD600 = ~0.05 for MRSA and 0.75 μg mL^{-1} for material. Data are means±SD (*n*=6).

			$^1O_2$ (by SOSG)[c]				
	Negative control[ac]	ROS neutralization[abc]	Positive control[cd]	amino-N-GQD 9.1	ROS neutralization[bc]	amino-N-GQD 9.9	ROS neutralization[bc]
20 scans	236±6	235±6	1894±76	254±16	235±10	288±25	235±10
100 scans	236±5	236±6	2131±89	837±40	236±12	894±42	235±10
170 scans	234±7	235±5	2270±95	1006±52	235±10	1103±59	235±11
			$^1O_2$ (by *t*-MVP)[e]				
	Negative control[ae]	ROS neutralization[abe]	Positive control[de]	amino-N-GQD 9.1	ROS neutralization[be]	amino-N-GQD 9.9	ROS neutralization[be]
20 scans	315±19	316±16	6024±130	379±24	315±14	414±30	316±15
100 scans	315±21	316±18	6196±154	3286±98	315±14	3880±109	315±17
170 scans	316±20	317±20	8092±169	4522±124	316±15	5005±141	316±15-
			$O_2^{\bullet -}$ (by XTT)[f]				
	Negative control[af]	ROS neutralization[abf]	Positive Control[df]	amino-N-GQD 9.1	ROS neutralization[bf]	amino-N-GQD 9.9	ROS neutralization[bf]
20 scans	0	0	1.56±0.09	0.04±0.02	0.03±0.01	0.08.±0.03	0.01±0.01
100 scans	0	0	1.90±0.13	0.50±0.05	0.02±0.01	0.73.±0.05	0.02±0.01
170 scans	0	0	2.32±0.17	0.81±0.07	0.02±0.02	1.05±0.08	0.02±0.01
			$O_2^{\bullet -}$ (by GSH)[g]				
	Negative control[ag]	ROS neutralization[abg]	Positive Control[dg]	amino-N-GQD 9.1	ROS neutralization[bg]	amino-N-GQD 9.9	ROS neutralization[bg]
20 scans	1	0	86.5±3.8%	3.6±0.9%	0.2±0.1%	4.7±1.2%	0.1±0.1%
100 scans	0	0	90.3±4.1%	56.1±2.0%	0.1±0.1%	65.0±3.3%	0.2±0.1%
170 scans	0	0	97.1±4.5%	69.2±3.2%	0.1±0.1%	77.5±3.6%	0.1±0.1%
			H_2O_2, OH^-, $ONOO^-$ (by H_2DCFDA)[h]				
	Negative Control[ah]	ROS neutralization[abh]	Positive Control[dh]	amino-N-GQD 9.1	ROS Neutralization[bh]	amino-N-GQD 9.9	ROS Neutralization[bh]
20 scans	73±5	74±4	3199±66	202±13	74±4	263±17	72±5
100 scans	74±5	76±5	3436±69	361±24	73±6	401±28	73±5
170 scans	73±6	75±4	3784±80	605±31	76±4	656±30	74±4

[a]Negative control: only treat using reagent and laser radiation without using any material (0 μg mL^{-1}).

[b]ROS neutralization: with the treatments of nanomaterial, the laser irradiation and 30 ppm of antioxidant *α*-Tocopherol/methyl linoleate.

[c]SOSG reagent (Ex/Em: 488/525 nm) has a specific reactivity to generate fluorescence recorded by a PL spectrometer.

[d]Positive control: the treatment of 50 μM TBHP and laser irradiation.

[e]*t*-MVP (Ex/Em: 352/465 nm) can react with $^1O_2$, forming a dioxetane intermediate that generates fluorescence upon decomposition to 1-pyrenecarboxaldehyde, and monitored by a PL spectrometer.

[f]XTT would interact with $O_2^{\cdot -}$ and produce the XTT-formazan generating strong absorption (470 nm in wavelength).
[g]GSH containing a thiol-tripeptide can prevent damages to cellular or bacterial components caused by stress of oxidation. Thiol group from GSH can be oxidized to disulfide bond converting GSH to glutathione disulfide. GSH oxidation was used to determine the generated $O_2^{\cdot -}$. Loss of GSH (%) = (absorbance difference between of sample and negative control / absorbance of negative control) × 100 %.
[h]H_2DCFDA passes through cell membranes and converts itself into DCFH. In the presenceof H_2O_2, OH· and $ONOO^-$, DCFH is oxidized to DCF, which emits green fluorescence (Ex/Em: 498/522 nm). A spectrophotometer was employed for measurements.

Table S10. Amount of ROS generated by conducting TPE (222.7 nJ pixel^{-1} with 20, 100 and 170 scans; Ex: 960 nm) to amino-N-GQD 11.1–Ab$_{protein\ A}$- and amino-N-GQD 12.0–Ab$_{protein\ A}$-treated-MRSA was monitored, respectively [5,8-13]. Delivered dose: OD600 = ~0.05 for MRSA and 0.75 μg mL^{-1} for material. Data are means±SD (*n*=6).

			$^1O_2$ (by SOSG)[c]				
	Negative control[ac]	ROS neutralization[abc]	Positive control[cd]	amino-N-GQD 11.1	ROS neutralization[bc]	amino-N-GQD 12.0	ROS neutralization[bc]
20 scans	235±7	234±11	1933±80	328±29	233±14	366±32	232±13
100 scans	235±7	233±12	2051±88	931±45	235±14	982±45	234±12
170 scans	237±8	236±11	2330±94	1096±60	235±15	1213±67	233±12

			$^1O_2$ (by *t*-MVP)[e]				
	Negative control[ae]	ROS neutralization[abe]	Positive control[de]	amino-N-GQD 11.1	ROS neutralization[be]	amino-N-GQD 12.0	ROS neutralization[be]
20 scans	318±16	316±16	6058±132	469±33	315±14	505±36	315±14
100 scans	319±18	315±15	7064±147	3865±105	315±14	4556±117	316±15
170 scans	318±16	315±15	8047±165	4972±132	316±15	5403±145	316±15

			$O_2\bullet^-$ (by XTT)[f]				
	Negative control[af]	ROS neutralization[abf]	Positive Control[df]	amino-N-GQD 11.1	ROS neutralization[bf]	amino-N-GQD 12.0	ROS neutralization[bf]
20 scans	1	0	1.51±0.07	0.12±0.03	0.02±0.01	0.17±0.05	0.01±0.01
100 scans	0	0	1.94±0.12	0.56±0.05	0.01±0.01	0.79±0.07	0.01±0.01
170 scans	0	0	2.43±0.15	0.96±0.06	0.03±0.01	1.16±0.09	0.02±0.01

			$O_2\bullet^-$ (by GSH)[g]				
	Negative control[ag]	ROS neutralization[abg]	Positive Control[dg]	amino-N-GQD 11.1	ROS neutralization[bg]	amino-N-GQD 12.0	ROS neutralization[bg]
20 scans	1	0	86.3±4.2%	6.7±1.2%	0.2±0.1%	8.0±1.4%	0.2±0.1%
100 scans	0		93.0±4.5%	64.0±3.0%	0.2±0.1%	71.7±3.2%	0.1±0.1%
170 scans	0	0	97.9±4.8%	75.7±3.4%	0.2±0.1%	81.1±3.7%	0.1±0.1%

			H_2O_2, OH^-, $ONOO^-$ (by H_2DCFDA)[h]				
	Negative Control[ah]	ROS neutralization[abh]	Positive Control[dh]	amino-N-GQD 11.1	ROS Neutralization[bh]	amino-N-GQD 12.0	ROS Neutralization[bh]
20 scans	74±3	75±3	3229±60	247±25	74±4	422±25	75±5
100 scans	74±5	72±3	3402±68	361±28	73±3	559±32	74±4
170 scans	76±5	75±4	3741±78	748±36	75±6	1003±37	75±6

[a]Negative control: only treat using reagent and laser radiation without using any material (0 μg mL^{-1}).

[b]ROS neutralization: with the treatments of nanomaterial, the laser irradiation and 30 ppm of antioxidant α-Tocopherol/methyl linoleate.

[c]SOSG reagent (Ex/Em: 488/525 nm) has a specific reactivity to generate fluorescence recorded by a PL spectrometer.

[d]Positive control: the treatment of 50 μM TBHP and laser irradiation.

[e]*t*-MVP (Ex/Em: 352/465 nm) can react with $^1O_2$, forming a dioxetane intermediate that generates fluorescence upon decomposition to 1-pyrenecarboxaldehyde, and monitored by a PL spectrometer.

[f]XTT would interact with $O_2^{\cdot -}$ and produce the XTT-formazan generating strong absorption (470 nm in wavelength).

[g]GSH containing a thiol-tripeptide can prevent damages to cellular or bacterial components caused by stress of oxidation. Thiol group from GSH can be oxidized to disulfide bond converting GSH to glutathione disulfide. GSH oxidation was used to determine the generated $O_2^{\cdot -}$. Loss of GSH (%) = (absorbance difference between of sample and negative control / absorbance of negative control) × 100 %.

[h]H_2DCFDA passes through cell membranes and converts itself into DCFH. In the presenceof H_2O_2, OH· and $ONOO^-$, DCFH is oxidized to DCF, which emits green fluorescence (Ex/Em: 498/522 nm). A spectrophotometer was employed for measurements.

Table S11. Amount of ROS generated by conducting TPE (222.7 nJ pixel^{-1} with 20, 100 and 170 scans; Ex: 960 nm) to amino-N-GQD 9.1- and amino-N-GQD 9.9-treated-MRSA was monitored, respectively [5,8-13]. Delivered dose: OD600 = ~0.05 for MRSA and 0.75 μg mL^{-1} for material. Data are means±SD (n=6).

			$^1O_2$ (by SOSG)[c]				
	Negative control[ac]	ROS neutralization[abc]	Positive control[cd]	amino-N-GQD 9.1	ROS neutralization[bc]	amino-N-GQD 9.9	ROS neutralization[bc]
20 scans	235±7	235±9	1843±66	243±11	235±10	258±20	235±9
100 scans	236±9	235±9	2090±82	448±30	236±9	481±29	234±11
170 scans	235±9	236±10	2217±87	521±34	235±11	595±39	235±11
			$^1O_2$ (by *t*-MVP)[e]				
	Negative control[ae]	ROS neutralization[abe]	Positive control[de]	amino-N-GQD 9.1	ROS neutralization[be]	amino-N-GQD 9.9	ROS neutralization[be]
20 scans	319±18	318±14	6006±128	310±16	316±14	381±26	316±16
100 scans	318±14	317±17	6204±152	1043±64	316±16	1162±67	317±17
170 scans	318±16	318±20	8069±171	1279±77	317±16	1340±80	316±14-
			$O_2\bullet^-$ (by XTT)[f]				
	Negative control[af]	ROS neutralization[abf]	Positive Control[df]	amino-N-GQD 9.1	ROS neutralization[bf]	amino-N-GQD 9.9	ROS neutralization[bf]
20 scans	0	0	1.57±0.08	0.03±0.01	0.02±0.01	0.05.±0.02	0.01±0.01
100 scans	0	0	1.95±0.11	0.27±0.03	0.02±0.01	0.38.±0.04	0.01±0.01
170 scans	0	0	2.33±0.17	0.41±0.05	0.01±0.01	0.51± 0.08	0.02±0.01
			$O_2\bullet^-$ (by GSH)[g]				
	Negative control[ag]	ROS neutralization[abg]	Positive Control[dg]	amino-N-GQD 9.1	ROS neutralization[bg]	amino-N-GQD 9.9	ROS neutralization[bg]
20 scans	0	0	84.6±3.5%	1.8±0.4%	0.1±0.1%	3.1±0.8%	0.2±0.1%
100 scans	0	0	90.5±4.2%	26.9±1.3%	0.2±0.1%	31.6±1.5%	0.1±0.1%
170 scans	0	0	95.5±4.3%	36.6±1.6%	0.2±0.1%	41.9±2.0%	0.2±0.1%
			H_2O_2, OH^-, $ONOO^-$ (by H_2DCFDA)[h]				
	Negative Control[ah]	ROS neutralization[abh]	Positive Control[dh]	amino-N-GQD 9.1	ROS Neutralization[bh]	amino-N-GQD 9.9	ROS Neutralization[bh]
20 scans	75±6	73±5	3208±59	105±9	74±5	113±16	75±4
100 scans	75±4	75±6	3391±62	177±16	75±6	209±18	74±5
170 scans	73±2	74±5	3733±70	295±20	73±6	376±28	75±3

[a]Negative control: only treat using reagent and laser radiation without using any material (0 μg mL^{-1}).

[b]ROS neutralization: with the treatments of nanomaterial, the laser irradiation and 30 ppm of antioxidant α-Tocopherol/methyl linoleate.

[c]SOSG reagent (Ex/Em: 488/525 nm) has a specific reactivity to generate fluorescence recorded by a PL spectrometer.

[d]Positive control: the treatment of 50 μM TBHP and laser irradiation.

[e]*t*-MVP (Ex/Em: 352/465 nm) can react with $^1O_2$, forming a dioxetane intermediate that generates fluorescence upon decomposition to 1-pyrenecarboxaldehyde, and monitored by a PL spectrometer.

[f]XTT would interact with $O_2^{\cdot-}$ and produce the XTT-formazan generating strong absorption (470 nm in wavelength).

[g]GSH containing a thiol-tripeptide can prevent damages to cellular or bacterial components caused by stress of oxidation. Thiol group from GSH can be oxidized to disulfide bond converting GSH to glutathione disulfide. GSH oxidation was used to determine the generated $O_2^{\cdot -}$. Loss of GSH (%) = (absorbance difference between of sample and negative control / absorbance of negative control) × 100 %.
[h]H_2DCFDA passes through cell membranes and converts itself into DCFH. In the presenceof H_2O_2, OH· and $ONOO^-$, DCFH is oxidized to DCF, which emits green fluorescence (Ex/Em: 498/522 nm). A spectrophotometer was employed for measurements.

Table S12. Amount of ROS generated by conducting TPE (222.7 nJ pixel^{-1} with 20, 100 and 170 scans; Ex: 960 nm) to amino-N-GQD 11.1- and amino-N-GQD 12.0-treated-MRSA was monitored, respectively [5,8-13]. Delivered dose: OD_{600} = ~0.05 for MRSA and 0.75 μg mL^{-1} for material. Data are means±SD (*n*=6).

			$^1O_2$ (by SOSG)[c]				
	Negative control[ac]	ROS neutralization[abc]	Positive control[cd]	amino-N-GQD 11.1	ROS neutralization[bc]	amino-N-GQD 12.0	ROS neutralization[bc]
20 scans	234±6	236±11	1925±79	268±17	234±12	310±21	233±11
100 scans	235±4	237±10	2028±84	494±34	235±15	514±35	234±12
170 scans	235±7	234±12	2290±91	563±38	235±16	633±43	231±12
			$^1O_2$ (by *t*-MVP)[e]				
	Negative control[ae]	ROS neutralization[abe]	Positive control[de]	amino-N-GQD 11.1	ROS neutralization[be]	amino-N-GQD 12.0	ROS neutralization[be]
20 scans	317±15	316±17	6040±134	334±23	316±17	407±31	315±14
100 scans	316±15	316±17	7042±150	1102±67	316±15	1207±45	316±14
170 scans	316±17	315±15	8033±168	1317±83	314±17	1392±88	317±13
			$O_2\bullet^-$ (by XTT)[f]				
	Negative control[af]	ROS neutralization[abf]	Positive Control[df]	amino-N-GQD 11.1	ROS neutralization[bf]	amino-N-GQD 12.0	ROS neutralization[bf]
20 scans	0	0	1.52±0.08	0.06±0.02	0.01±0.01	0.13±0.05	0.01±0.01
100 scans	0	0	2.03±0.15	0.31±0.05	0.01±0.01	0.45±0.07	0.02±0.01
170 scans	0	0	2.46±0.17	0.49±0.07	0.02±0.01	0.58±0.11	0.01±0.01
			$O_2\bullet^-$ (by GSH)[g]				
	Negative control[ag]	ROS neutralization[abg]	Positive Control[dg]	amino-N-GQD 11.1	ROS neutralization[bg]	amino-N-GQD 12.0	ROS neutralization[bg]
20 scans	0	0	86.6±4.5%	3.0±0.5%	0.2±0.1%	4.5±0.4%	0.1±0.1%
100 scans	0	0	94.1±4.9%	30.6±1.6%	0.1±0.1%	37.1±2.0%	0.2±0.1%
170 scans	0	0	98.7±5.4%	40.8±2.5%	0.1±0.1%	45.8±2.4%	0.2±0.1%
			H_2O_2, OH^-, $ONOO^-$ (by H_2DCFDA)[h]				
	Negative Control[ah]	ROS neutralization[abh]	Positive Control[dh]	amino-N-GQD 11.1	ROS Neutralization[bh]	amino-N-GQD 12.0	ROS Neutralization[bh]
20 scans	75±5	75±4	3211±58	117±9	75±4	148±13	75±6
100 scans	73±5	74±6	3405±66	205±18	75±6	234±20	73±5
170 scans	74±4	75±4	3702±75	320±25	73±5	396±28	75±5

[a]Negative control: only treat using reagent and laser radiation without using any material (0 μg mL^{-1}).

[b]ROS neutralization: with the treatments of nanomaterial, the laser irradiation and 30 ppm of antioxidant *α*-Tocopherol/methyl linoleate.

[c]SOSG reagent (Ex/Em: 488/525 nm) has a specific reactivity to generate fluorescence recorded by a PL spectrometer.

[d]Positive control: the treatment of 50 μM TBHP and laser irradiation.

[e]*t*-MVP (Ex/Em: 352/465 nm) can react with $^1O_2$, forming a dioxetane intermediate that generates fluorescence upon decomposition to 1-pyrenecarboxaldehyde, and monitored by a PL spectrometer.

[f]XTT would interact with $O_2^{\cdot-}$ and produce the XTT-formazan generating strong absorption (470 nm in wavelength).

[g]GSH containing a thiol-tripeptide can prevent damages to cellular or bacterial components caused by stress of oxidation. Thiol group from GSH can be oxidized to disulfide bond converting GSH to glutathione disulfide. GSH oxidation was used to determine the generated $O_2^{\cdot-}$. Loss of GSH (%) = (absorbance difference between of sample and negative control / absorbance of negative control) × 100 %.

[h]H_2DCFDA passes through cell membranes and converts itself into DCFH. In the presenceof H_2O_2, OH· and $ONOO^-$, DCFH is oxidized to DCF, which emits green fluorescence (Ex/Em: 498/522 nm). A spectrophotometer was employed for measurements.

In summary of ROS results,

i) The amount of ROS generated from by a TPE to materials: the signals generated from all of the materials would exhibited the values directly (Tables S3 and S4).

ii) The amount of ROS generated from by a TPE to materials-Ab-treated-bacteria: the materials-Ab would bind with bacteria specifically. However, part of the signals generated from the materials-Ab was absorbed by the bacteria, leading to lower values (Tables S5-S6 and S9-S10) than i).

iii) The amount of ROS generated from by a TPE to materials-treated-bacteria:

without Ab, the amount of materials interacted with bacteria via the electrostatic interaction (Scheme S2b) or endocytosis (Tables S7-S8 and S11-S12) would be less than ii).

As a result, the results were obtained.

Table S13. Antibody conjugation efficiency (%) and zeta potential (mV) of materials [Cell type: DTS1060C; Measurement duration: 30 number of runs, 20 run duration (sec)]. Delivered dose: 7.5 mg mL^{-1} material.

	1XPBS	*E. coli* LB broth	MRSA BHI broth
amino-N-GQD 9.1-Ab_{LPS}	9.9% / 5.4 mV	10.3% / 6.2 mV	
amino-N-GQD 9.9-Ab_{LPS}	10.6% / 6.9 mV	11.0% / 7.9 mV	
amino-N-GQD 11.1-Ab_{LPS}	12.3% / 7.7 mV	12.1% / 9.0 mV	
amino-N-GQD 12.0-Ab_{LPS}	13.8% / 8.5 mV	14.3% / 9.6 mV	
amino-N-GQD 9.1-$Ab_{protein\ A}$	9.0% / 5.9 mV		11.0% / 7.1 mV
amino-N-GQD 9.9-$Ab_{protein\ A}$	10.1% / 7.2 mV		11.9% / 8.4 mV
amino-N-GQD 11.1-$Ab_{protein\ A}$	11.5% / 8.0 mV		13.1% / 9.6 mV
amino-N-GQD 12.0-$Ab_{protein\ A}$	13.0 % / 8.2 mV		15.2% / 10.5 mV

Table S14. Estimation of the molecular weight of sorted amino-N-GQDs.

amino-N-GQD 9.1 (9.1±0.2 nm)

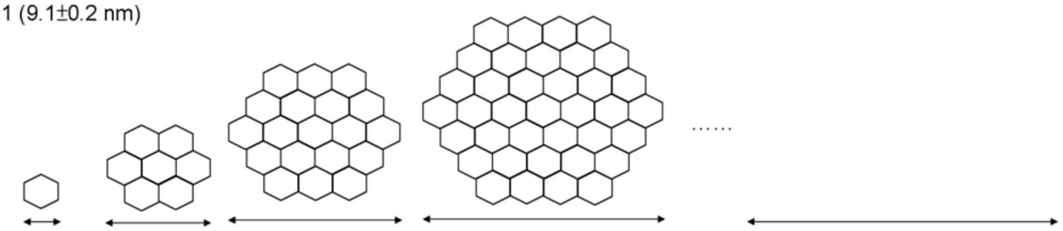

	0.243nm	0.729nm	1.215nm	1.701nm	9.1nm
Benzene number	1	7	19	37	1027
Approximate molecular weight	72	290	653	1162	26217

amino-N-GQD 9.9 (9.9±0.2 nm)

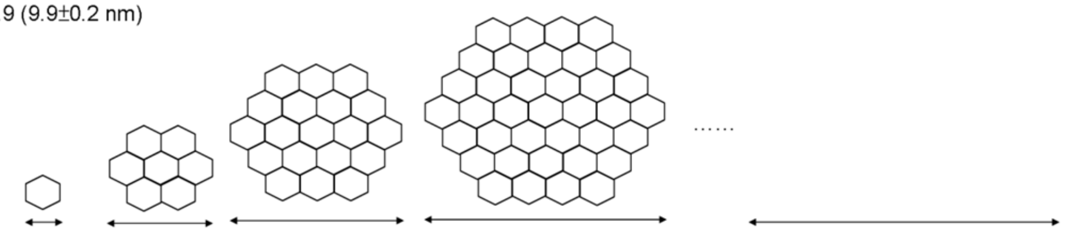

	0.243nm	0.729nm	1.215nm	1.701nm	9.9nm
Benzene number	1	7	19	37	1261
Approximate molecular weight	72	290	653	1162	32027

amino-N-GQD 11.1 (11.1±0.3 nm)

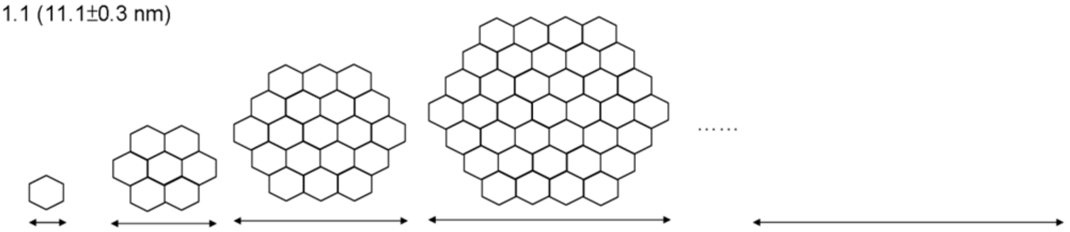

	0.243nm	0.729nm	1.215nm	1.701nm	11.1nm
Benzene number	1	7	19	37	1519
Approximate molecular weight	72	290	653	1162	38418

amino-N-GQD 12.0 (12.0±0.3 nm)

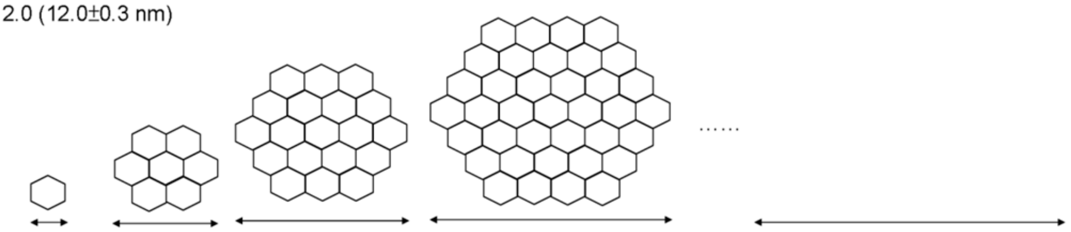

	0.243nm	0.729nm	1.215nm	1.701nm	12.0nm
Benzene number	1	7	19	37	1801
Approximate molecular weight	72	290	653	1162	45390

Table S15. TPE action cross sections of Fluorescein (in ddH_2O, pH 11) and Rhodamine B (in methanol). Delivered dose: 0.75 μg mL^{-1} material.

	Fluorescein (in ddH_2O, pH=11)	Rhodamine B (in methanol)
Excitation wavelength at 960 nm action cross section, $\eta\sigma_2$ (GM, $10^{-50}cm^4s$/photon)	15.2	13.4

Table S16. TPE cross section of materials at excitation wavelength of 960 nm. Delivered dose: 0.75 μg mL^{-1} material.

Reference	Integrated emission intensity (counts)		Action cross-section (ησ)
Fluorescein [a]	60.17		15.2
Analyte	Integrated emission intensity (counts)	Absolute quantum yield (η)	Absolute cross-section (σ)
amino-N-GQD 9.1	87242	0.39	56510
amino-N-GQD 9.9	89531	0.41	57993
amino-N-GQD 11.1	92384	0.45	59841
amino-N-GQD 12.0	94867	0.48	61449

[a]Fluorescein was selected as a reference to determine the TPE cross section. The relevant calculations are shown in the Materials and methods.

References

1. Hummers, W. S.; Offeman, R. E. Preparation of graphitic oxide. *J. Am. Chem. Soc.* **1958**, *80*, 1339.

2. Ning, X.; Guo, J.; Wolfert, M. A;. Boons, G. J. Visualizing metabolically labeled glycoconjugates of living cells by copper-free and fast huisgen cycloadditions. *Angew. Chem. Int. Ed.* **2008**, *47*, 2253−2255.

3. Rizvi, A. H.; Camara, P. G.; Kandror, E. K.; Roberts, T. J.; Schieren, I.; Maniatisa, T.; Rabadan, R. Single-cell topological RNA-seq analysis reveals insights into cellular differentiation and development. *Nat. Biotechnol.* **2017**, *35*, 551−560.

4. Shi, L.; Hernandez, B.; Selke, M. Singlet oxygen generation from water-soluble quantum dot-organic dye nanocomposites. *J. Am. Chem. Soc.* **2006**, *128*, 6278–6279.

5. Kuo, W. S.; Chang, C. Y.; Chen, H. H.; Hsu, C. L. L.; Wang, J. Y.; Kao, H. F.; Chou, L. C. S.; Chen, Y. C.; Chen, S. J.; Chang, W. T.; Tseng, S. W.; Wu, P. C.; Pu, Y. P. Two-photon photoexcited photodynamic therapy and contrast agent with antimicrobial graphene quantum dots. *ACS Appl. Mater. Interfaces* **2016**, *8*, 30467 −30474.

6. Xu, C.; Zipfel, X.; Shear, J. B.; Williams, R. W.; Webb, W. W.Multiphoton fluorescence excitation: new spectral windows for biological nonlinear microscopy. *Proc. Natl. Acad. Sci. USA* **1996**, *93*, 10763−10768.

7. Lakowicz, J.R. *Principles of Fluorescence Spectroscopy*, 3rd ed.; Springer US, Baltimore, MD, USA, 2010.

8. Carmel-Hare, O.; Storz, G. Roles of the glutathione-and yhioredoxin-dependent reduction systems in the *Escherichia coli* and *Saccharomyces cerevisiae* responses to oxidative stress. *Annu. Rev. Microbiol.* 2000, **54**, 439−461.

9. Ellman, G. L. Tissue sulfhydryl groups. *Arch. Biochem. Biophys.***1959**, *82*, 70−77.

10. Kinen, M. M.; Kamal-Eldin, A.; Lampi, A. M.; Hopia, A.Effects of α- and γ-tocopherols on formation of hydroperoxides and tow decomposition products from methyl linoleate. *J. Am. Oil Chem. Soc.* **2000**, *77*, 801−806.

11. Possel, H.; Noack, H.; Augustin, W.; Keilhoff, G.; Wolf, G. 7-Dihydrodichlorofluorescein diacetate as a fluorescent marker for eroxynitrite formation. *FEBS Lett.* **1997**, *416*, 175−178.

12. Sharma, P.; Jha, A. B.; Dubey, R. S.; Pessarakli, M. Reactive oxygen species, oxidative damage, and antioxidative defense mechanism in plants under stressful conditions. *J. Bot.* **2012**, *2012*, 1−26.

13. Thompson, A.; Lever, J. R.; Canella, K. A.; Miura, A.; Posner, G. H.; Seliger, H. H. Chemiluminescence mechanism and quantum yield of synthetic vinylpyrene analogs of benzo[a]pyrene-7,8-dihydrodiol. . *J. Am. Chem. Soc.* **1986**, *108*, 4498−4504.

14. Zheng, X. T.; Ananthanarayanan, A.; Luo, K. Q.; Chen, P. Glowing graphene quantum dots and carbon dots: properties, syntheses, and biological applications. *Small* **2015**, *11*, 1620−1636.

15. Zhu, S.; Song, Y.; Zhao, X.; Shao, J.; Zhang, J.; Yang, B The photoluminescence mechanism in carbon dots (graphene quantum dots, carbon nanodots, and polymer dots): current state and future perspective.

Nano Res. **2015**, *8*, 355−381.
16. Ferrari, A. C.; Basko, D. M. Raman spectroscopy as a versatile tool for studying the properties of Graphen. *Nat. Nanotechnol.* **2013**, *8,* 235–246.
17. Tuinstra, F.; Koenig, J. L. Raman spectrum of graphite. *J. Phys. Chem.* **1970**, *53*, 1126–1130.
18. Ferrari, A. C.; Robertson, J. Interpretation of Raman spectra of disordered and amorphous carbon. *Phys. Rev. B* **2000**, *61*, 14095−14107.
